# Drug‐Coated Balloons Versus Other Percutaneous Coronary Intervention Strategies in De Novo Coronary Artery Disease: A Systematic Review, Meta‐Analysis With Trial Sequential Analysis

**DOI:** 10.1155/cdr/5568664

**Published:** 2026-01-01

**Authors:** Yue Yu, Yue Miao Jiao, Yang Li, Ming Zhang, Guang Yuan Song, Cheng Qian Yin

**Affiliations:** ^1^ Department of Cardiology, Beijing Anzhen Hospital, Capital Medical University, Beijing, China, ccmu.edu.cn

**Keywords:** de novo coronary artery disease, drug-coated balloons, meta-analyses, percutaneous coronary interventions, trial sequential analyses

## Abstract

**Background:**

Drug‐coated balloons (DCBs) present a viable option distinct from drug‐eluting stents (DESs) in addressing coronary artery disease (CAD), particularly when it comes to in‐stent restenosis (ISR) scenarios. The utilization of DCBs marks a significant deviation from traditional DES applications. For CAD patients experiencing ISR, DCBs offer an innovative pathway to treatment. However, their efficacy and safety in de novo CAD lesions remain uncertain. This meta‐analysis evaluates DCB versus other percutaneous coronary intervention (PCI) strategies, including DES, bare‐metal stents (BMSs), and plain old balloon angioplasty (POBA), in de novo CAD.

**Methods:**

A comprehensive search was conducted across PubMed, Embase, Web of Science, and the Cochrane Central Register of Controlled Trials (CENTRAL), spanning from their inception up until November 14, 2024. Studies included were randomized controlled trials (RCTs) and cohort studies, which assessed DCB against alternative PCI strategies in patients with de novo CAD. The key outcome measures focused on major adverse cardiac events (MACEs) as well as late lumen loss (LLL). Trial sequential analysis (TSA) assessed the robustness of findings.

**Results:**

Fifty studies (19 RCTs, 31 cohort studies) involving 28,292 patients were analyzed. DCB showed a lower MACE incidence compared to noncoated devices (RR = 0.52, 95% CI: 0.40–0.69) but no significant difference versus DES (RR = 0.96, 95% CI: 0.84–1.10). DCB significantly reduced LLL compared to all controls (MD = −0.22, 95% CI: −0.29 to −0.14). Subgroup analyses confirmed DCB′s safety across indications, ethnicities, comorbidities, and dual antiplatelet therapy (DAPT) durations, with reduced LLL in small vessel disease and shorter DAPT. TSA supported LLL findings but indicated inconclusive MACE results in RCTs, necessitating further research.

**Conclusion:**

DCB is a safe and effective alternative to DES in de novo CAD, with comparable safety and superior LLL reduction. However, MACE results in RCTs require further validation. Future studies should explore long‐term outcomes and integrate newer‐generation DCB and DES technologies to optimize clinical practice.

## 1. Introduction

Globally, cardiovascular diseases have soared in prevalence, emerging prominently as a principal contributor to mortality. Among these, coronary artery disease (CAD) stands out as a significant factor; a primary subset of cardiovascular diseases, it poses a substantial health and economic burden [1–3].

The implantation of drug‐eluting stents (DESs) is the standard interventional treatment for CAD [4, 5]. Nonetheless, stent implantation continues to encounter considerable challenges, as the presence of metal stents within the vessels can distort and restrict coronary arteries, impede vascular pulsation and adaptive remodeling, and induce chronic inflammation [6, 7]. Despite advancements in second‐generation DES, the occurrence of late‐stage thrombosis remains a persistent issue [8, 9]. Conversely, drug‐coated balloons (DCBs) offer the advantage of delivering uniformly distributed, high concentrations of antirestenosis drugs directly to the target lesions of culprit coronary arteries without the need for durable polymers or stent structures, thereby effectively mitigating the limitations associated with DES [10]. Multiple expert consensuses and guidelines have accorded endorsement to DCBs as an interventional therapy for in‐stent restenosis (ISR), carrying a recommendation grade of IA, as cited in references [5, 10]. DCBs have exhibited both safety and effectiveness across diverse lesion types, encompassing large vessels and bifurcation lesions, as detailed in studies [11–13]. Nonetheless, the exploration into the utilization of DCBs in de novo CAD remains constrained, with the existing research findings displaying discrepancies.

Several studies and reviews have hinted at the comparable safety and effectiveness of DCBs versus DES in managing noncomplex lesions of de novo CAD, as reported in references [14, 15]. Nonetheless, recent data from a large, multicenter randomized controlled trial (RCT) have revealed that DCBs did not meet the criterion for noninferiority when compared to DES, which remains the favored option for such lesions, as detailed in [16]. The objective of this meta‐analysis is to consolidate the existing evidence and assess the DCB approach in contrast to other percutaneous coronary intervention (PCI) techniques for treating de novo coronary artery lesions.

## 2. Method

### 2.1. Search Methodology

An extensive search encompassing PubMed, Embase, Web of Science, and the Cochrane Central Register of Controlled Trials (CENTRAL) was undertaken, spanning from their establishment to November 14, 2024. The primary objective was to uncover RCTs and cohort studies that compared the efficacy of the DCBs approach with alternative PCI techniques for the management of de novo CAD. The search utilized a combination of tailored algorithms and relevant keywords, with no language or filter restrictions. Detailed search strategies for each database are provided in Table S1. This meta‐analysis adhered to the PRISMA guidelines [11].

### 2.2. Study Selection Criteria

Studies were considered eligible based on the following criteria: they had to (1) be designed in the format of RCTs or cohort studies; (2) evaluate the DCB strategy, with the possibility of bailout stenting, against control interventions including plain old balloon angioplasty (POBA), bare‐metal stents (BMSs), or DES for the target lesions in question; (3) enroll patients diagnosed with de novo CAD; and (4) furnish angiographic or clinical outcome data, without any constraints on the duration of follow‐up. For studies reporting multiple follow‐up periods, the longest available outcomes were utilized.

Studies were excluded if they (1) focused on DCB for ISR; (2) investigated DCB for dysfunctional hemodialysis arteriovenous fistulas in peripheral artery disease; (3) were unpublished observational studies, registries, or conference abstracts; or (4) lacked complete baseline or outcome data.

### 2.3. Study Outcomes

The key outcomes under evaluation encompassed major adverse cardiac events (MACEs) alongside late lumen loss (LLL). Additionally, secondary endpoints were target lesion revascularization (TLR), mortality from all causes, death attributed to cardiac issues, myocardial infarction (MI), the percentage of diameter stenosis (DS%), and the minimum lumen diameter (MLD).

MACE was defined as a composite of cardiac death, MI, and TLR. This definition is consistent with those used in key RCTs of DCBs, including REC‐CAGEFREE I [12], DEBUT [13], and PICCOLETO II [14]. Event data were extracted according to this definition whenever available. Some studies did not provide an explicit definition of MACE or used broader endpoint compositions (for example, including stroke or all‐cause mortality). These studies were included in the overall analysis but were further evaluated in a sensitivity analysis to assess the impact of nonstandard or undefined endpoint definitions.

### 2.4. Data Extraction Process

Two independent reviewers (Y.Y. and Y.M.J.) screened all potentially relevant studies. The screening process initially involved excluding studies based solely on their titles and abstracts. Subsequently, a meticulous review of the full texts was conducted for studies that appeared to meet the eligibility criteria. Final inclusion required consensus between the reviewers, with discrepancies resolved through discussion and strict adherence to the predefined criteria.

Data extraction was carried out by two consistent reviewers utilizing a standardized Microsoft Excel database system. The extracted details encompassed study attributes such as geographical region, year of publication, study design, sample size, and the duration of follow‐up. Additionally, patient characteristics like age, gender, and coexisting medical conditions were recorded, as well as clinical presentation and target lesion characteristics. Additionally, details on PCI devices, strategies, baseline angiographic measurements, and follow‐up outcomes were recorded. Any disagreements during data extraction were resolved through consensus.

### 2.5. Evaluation of Research Quality and Potential Bias

The methodological rigor of the included studies was independently evaluated by two researchers, namely, Y.Y. and Y.M.J. For RCTs, the Cochrane Risk of Bias Tool was employed as the assessment instrument, as detailed in reference [17]. In the case of nonrandomized studies, the Newcastle–Ottawa Scale (NOS) served as the prevalent tool for gauging their methodological quality [15], such as cohort and case–control studies. The scale evaluates studies across three domains: selection of study participants (including representativeness of the study population and definition of exposure or control) and comparability of study groups (assessing control of confounding factors). Additionally, it evaluates outcome assessment, including the adequacy of outcome measurement methods and follow‐up duration. Each criterion is scored using a star system, with a maximum score of 9, where higher scores indicate higher study quality. Two reviewers independently performed quality evaluation, and any inconsistencies were addressed through discussion or by seeking input from a third reviewer.

### 2.6. Data Analysis

All meta‐analyses in this study were conducted using R language (Version 4.1.3, R Core Team, 2022) [16]. Data analysis primarily relied on the meta package (Version 8.0‐1) [18], which provides extensive functions for fitting fixed‐effect and random‐effects models, testing heterogeneity, and visualizing results. Summary statistics included risk ratios (RRs) accompanied by 95% confidence intervals (CIs) and mean differences (MDs) with standard deviations, with findings visualized using forest plots. If the data in the original literature were skewed and reported as medians and interquartile ranges, they were first transformed using conversion formulas [17, 19, 20]. If the formula was deemed unsuitable for conversion, a second method that did not consider data distribution [21] was used to convert the data into means and standard deviations for subsequent analysis. Statistical significance was determined by a *p* value less than 0.05 in a two‐tailed test. To assess variability among studies, both Cochran′s *Q* test and the *I* ^2^ statistic were utilized, as referenced in [22]. In the context of this study′s meta‐analysis, the *I* ^2^ statistic was employed to evaluate heterogeneity, where the *I* ^2^ value reflects the proportion of between‐study heterogeneity in the total variation. As per the Cochrane Handbook, an *I* ^2^ < 50*%* suggests low heterogeneity among studies, prompting the use of a fixed‐effect model for pooling effect sizes. Conversely, an *I* ^2^ ≥ 50*%* indicates substantial heterogeneity, leading to the application of a random‐effects model to more accurately estimate the effect size and its CI. Zero events were addressed using continuity correction. Due to the aggregation of data from multiple trials and the limited availability of reported outcomes, there is an increased likelihood of Type 1 and Type 2 errors. To mitigate this issue, trial sequential analysis (TSA) was employed to assess the reliability and conclusiveness of the evidence derived from the combined data [23–25].

The reliability of the evidence is considered sufficient and conclusive when the TSA curve′s z‐line crosses both the traditional significance boundary and the trial sequential monitoring boundary. Conversely, if the z‐line fails to cross either boundary, the findings are deemed inconclusive, indicating a need for additional studies. In the present meta‐analysis, the TSA was performed with an alpha error set at 5%, a power of 80% (beta error), and an assumed relative risk reduction of 20%. This approach ensures a robust assessment of the evidence while minimizing the potential for statistical errors.

The assessment of publication bias encompassed the utilization of funnel plots, alongside Egger′s test and Begg′s test [26], with a *p* value < 0.05 suggesting potential bias. Sensitivity analysis was carried out by sequentially removing individual trials to examine their impact on the overall results.

To confirm the robustness of the findings, a sensitivity analysis was performed by restricting the dataset to studies that explicitly used the standardized MACE definition (cardiac death, MI, and TLR). Studies reporting nonstandard MACE definitions were excluded from this subset. The results were compared with the overall estimates to determine consistency.

Subgroup analyses were performed for both DCB and control groups, stratified by factors such as clinical comorbidities, lesion characteristics, dual antiplatelet therapy duration, use of intravascular imaging guidance, and control group classification.

## 3. Results

### 3.1. Literature Search

**Figure 1:**
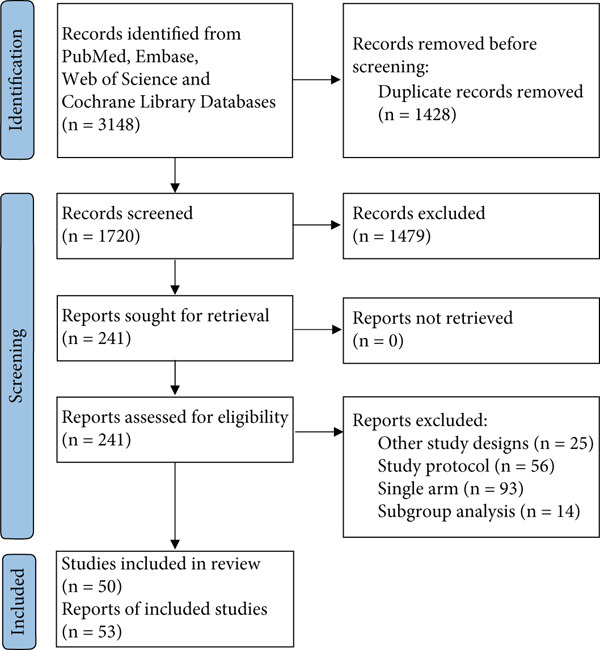
Study selection process flowchart.

A total of 3148 records were retrieved from the respective databases. Upon removing duplicates, the number of records reduced to 1720, which were then screened based on their titles and abstracts. This initial screening led to the identification of 241 articles that qualified for a comprehensive full‐text assessment. Out of these 241 articles, a meticulous selection process resulted in the inclusion of 50 studies—consisting of 19 RCTs and 31 cohort studies—in the meta‐analysis. Additionally, a manual backward citation analysis was conducted, but it did not yield any further eligible articles. The entire search and selection procedure is depicted in Figure 1.

### 3.2. Characteristics of Included Studies

Our meta‐analysis included 50 studies [12–14, 22, 27–75], involving 28,292 patients. Table 1 shows the characteristics of the included studies. Among them, 8 studies targeted ACS (or STEMI, NSTEMI) populations [32, 36, 39, 45, 54, 56, 61, 69], and 8 studies focused on bifurcation lesions [28, 29, 38, 58–60, 62, 75]. Additionally, 11 studies targeted small vessel disease (or very small vessel disease) [14, 22, 27, 31, 33, 41, 44, 48, 49], 2 studies included populations at high bleeding risk [13, 35], and 4 studies targeted severely calcified lesions [52, 55, 64, 70]. The longest clinical follow‐up duration was 5 years [67]. Tables S2 and S3 present the baseline patient characteristics, comorbidities, and target lesion types of the DCB and control groups. Certain studies indicated variations in clinical comorbidities among patients; however, the baseline characteristics remained balanced between the DCB treatment group and the control group.

**Table 1:** The characteristics of the included studies.

**Study/first author**	**Year**	**Region**	**Design**	**Target lesion**	**Number**	**DCB type**	**DCB brand**	**Control**	**DES/POBA brand**	**Clinical follow-up**	**Angiographic follow-up**	**Primary endpoint**	**MACE definition**	**Bailout stenting rate (%)**	**DAPT duration**
PICCOLETO	2011	Italy	RCT	Small vessel disease	60	DCB (paclitaxel)	Dior	1st‐gen DES	Taxus Libertè	9 months	6 months	DS%	Death, MI, TLR	34.5	1/12 months
BELLO	2012	Italy	RCT	Small vessel disease	182	DCB (paclitaxel)	IN.PACT Falcon	1st‐gen DES	Taxus Libertè	6 months	6 months	LLL	Death, MI, TVR	20.2	1/12 months
BABILON	2014	Spain	RCT	Bifurcation lesions	108	DCB (paclitaxel)	SeQuent Please	2nd‐gen DES/BMS	XIENCE V/Coroflex	24 months	9 months	LLL	Death, MI, TLR	7.8	3/12 months
PEPCAD‐BIF	2016	Germany	RCT	Bifurcation lesions	64	DCB (paclitaxel)	SeQuent Please	POBA	NA	9 months	9 months	LLL	NA	0.0	1 month
Naoki Nishiyama	2016	Japan	RCT	De novo coronary lesions	60	DCB (paclitaxel)	SeQuent Please	2nd‐gen DES	Xience Prime/Xpedition	8 months	8 months	TLR	NA	10.0	8 months
Atsushi Funatsu	2017	Japan	RCT	Small vessel disease	133	DCB (paclitaxel)	SeQuent Please	POBA	SeQuent uncoated balloon	6 months	6 months	TVF	Cardiac death, MI, TLR	2.9	3 months
David Gobic	2017	Croatia	RCT	De novo lesions (STEMI)	78	DCB (paclitaxel)	SeQuent Please	2nd‐gen DES	Biomime	6 months	6 months	MACE, LLL	Cardiac death, MI, TLR, thrombosis	7.3	12 months
BASKET‐SMALL 2	2018	Europe	RCT	Small vessel disease	758	DCB (paclitaxel)	SeQuent Please	2nd‐gen DES	Xience/Taxus Element	36 months	NA	MACE	Cardiac death, MI, TVR	5.1	1/6 months
RESTORE SVD China	2018	China	RCT	Small vessel disease	230	DCB (paclitaxel)	Restore DCB	2nd‐gen DES	RESOLUTE DES	24 months	9 months	DS%	Death, MI, revascularization	5.2	6 months
DEBUT	2019	Finland	RCT	High bleeding risk	208	DCB (paclitaxel)	SeQuent Please	BMS	Integrit/Omega	9 months	NA	MACE	Cardiac death, MI, TLR	2.0	1 month
Eun‐Seok Shin	2019	Korea	RCT	High bleeding risk	40	DCB (paclitaxel)	SeQuent Please	BMS	Vision	12 months	9 months	LLL	Death, MI, repeat revascularization, recurrent ischemic symptoms	0.0	1 month
REVELATION	2019	Amsterdam	RCT	De novo lesions (STEMI)	120	DCB (paclitaxel)	Pantera Lux	2nd‐gen DES	Orsiro/Xience	9 months	9 months	FFR	Cardiac death, MI, TLR	18.0	12 months
PICCOLETO II	2020	Europe	RCT	Small vessel disease	232	DCB (paclitaxel)	Elutax SV/Emperor	2nd‐gen DES	Xience	36 months	6 months	LLL	Cardiac death, MI, TLR	6.7	CCS 1/NA; ACS 12 months
BEYOND	2020	China	RCT	Bifurcation lesions	222	DCB (paclitaxel)	Bingo	POBA	Yinyi	9 months	9 months	TLS	Death, MI, stroke, TVR	0.0	12 months
PEPCAD NSTEMI	2020	Germany	RCT	De novo lesions (NSTEMI)	210	DCB (paclitaxel)	SeQuent Please NEO	2nd‐gen DES/BMS	NA	9 months	NA	TLF	Death, MI, stroke, revascularization or PCI at other vessels	14.6	12 months
BIO‐RISE CHINA	2022	China	RCT	Small vessel disease	212	DCB (sirolimus)	Biolimus A9 (BA9)	POBA	NA	12 months	9 months	LLL	Death, MI, revascularization	2.8	NA
Yu	2022	China	RCT	De novo coronary lesions	170	DCB (paclitaxel)	SeQuent Please	2nd/3rd‐gen DES	Resolute/Xience Xpedition/SYNERGY/Firehawk	12 months	9 months	LLL, MACE	Death, MI, TLR, TVR	2.4	CCS 1–3/12 months ACS 6–12/12 months
PEPCAD China SVD	2023	China	RCT	Small vessel disease	268	DCB (paclitaxel)	SeQuent Please	POBA	SeQuent Neo	12 months	9 months	LLL	All‐cause deaths, MI, and revascularization	2.1	12 months
REC‐CAGEFREE I	2024	China	RCT	De novo noncomplex coronary artery disease	2272	DCB (paclitaxel)	Swide DCB	2nd‐gen DES	Firebird2	24 months	NA	DoCE	Cardiac death, MI, TLR	9.4	1 month
F. Nijhoff	2015	Europe	Retrospective cohort study	De novo lesions (STEMI)	140	DCB (paclitaxel)	Dior	1st‐gen DES/BMS	Taxus Liberté/Magic	12 months	6 months	LLL	Death, MI, TVR	10.0	12 months
Ae‐Young Her	2016	Korea	Retrospective cohort study	De novo coronary lesions	72	DCB (paclitaxel)	SeQuent Please	POBA	NA	9 months	9 months	LLL	NA	NA	≥ 6 weeks
Eun‐Seok Shin	2016	Korea	Prospective cohort study	De novo coronary lesions	66	DCB (paclitaxel)	SeQuent Please	2nd‐gen DES/BMS	Resolute/Xience prime/Vision	12 months	9 months	LLL	Cardiac death, MI, TLR, arterial thrombosis	0.0	6 weeks/12 months
DASDO ANTONIUS SINAGA	2016	Singapore	Retrospective cohort study	Small vessel disease	335	DCB (paclitaxel)	SeQuent Please	2nd‐gen DES	NA	12 months	NA	MACE	Death, recurrent MI, repeat revascularization	NA	6/12 months
Francesco Giannini	2017	Italy	Retrospective cohort study	Small vessel disease	181	DCB (paclitaxel)	IN.PACT	2nd‐gen DES	Xience V/Promus	12 months	NA	MACE	Cardiac death, MI, TVR	20.2	1/12 months
Ae‐Young Her	2018	Korea	Prospective cohort study	De novo coronary lesions	151	DCB (paclitaxel)	SeQuent Please	2nd‐gen DES/BMS	Resolute/Xience prime/Vision	12 months	9 months	LLL, MACE	Cardiac death, MI, TLT, or repeat revascularization	0.0	1/12 months
H. W. Sim	2018	Singapore	Retrospective cohort study	Very small disease	287	DCB (paclitaxel)	SeQuent Please/Sequent Neo/In.Pact Falcon	2nd‐gen DES	Xience Xpedition SV/Xience Alpine/Resolute Onyx	12 months	NA	TLF	Cardiac mortality, MI, and TLR	8.0	CCS 1/12 months, ACS 12 months
Katsumi Ueno	2019	Japan	Retrospective cohort study	Severely calcified coronary lesions	123	DCB (paclitaxel)	SeQuent Please	2nd‐gen DES	Xience\Ultimaster\Nobori\Premiere\Synergy\Resolute	732 days	6 months	TLR, TVR	NA	1.9	NA
A. Silverio	2020	Sweden	Retrospective cohort study	Small vessel disease	14,788	DCB (paclitaxel)	SeQuent Please/In.Pact Falcon/Pantera Lux	2nd/3rd‐gen DES	Endeavor Resolute/Resolute Integrity/Resolute Onyx/XIENCE PRIME/XIENCE Xpedition/XIENCE ProX/PROMUS Element Plus/Promus PREMIER/Synergy/Orsiro/BioMatrix/Ultimaster	36 months	NA	Restenosis, TLT	NA	NA	NA
D. Zhang	2020	China	Retrospective cohort study	ACS	380	DCB (paclitaxel)	SeQuent Please	2nd‐gen DES	NA	3 months	NA	MACE	Cardiac death, MI, TVR, ISR	2.8	12 months
Yoshihiro Iwasaki	2021	Japan	Retrospective cohort study	Severely calcified coronary lesions	157	DCB (paclitaxel)	SeQuent Please	2nd/3rd‐gen DES	Xience\Synergy\Nobori\Ultimaster\Resolute Onyx\Biofreedom	12 months	12 months	MACE	Cardiac death, noncardiac death, MI, TLR, and major bleeding (BARC ≥ Type 3)	NA	3/12 months
Qiang Tan	2021	China	Retrospective cohort study	Small vessel disease with AMI	268	DCB (paclitaxel)	Sequent Please	2nd‐gen DES	Endeavor Resolute\Firebird‐2	24 months	9–12 months	MACE	All‐cause death, MI, TLR, and TVR	NA	3/12 months
Chuang Li	2022	China/Singapore	Retrospective cohort study	De novo ostial lesions (LAD)	98	DCB (paclitaxel)	Sequent Please	2nd‐gen DES	Resolute\Xience\Promus\Excel	12 months	NA	MACE	Cardiac death, TLR, TVR, and recurrent MI	2.0	NA
Youmei Li	2022	China	Retrospective cohort study	Bifurcation lesions	219	DCB (paclitaxel)	Sequent Please	POBA	Quantum Maverick	12 months	12 months	MACE	Cardiac death, MI, and unstable angina requiring admission	NA	NA
Hengdao Liu	2022a	China	Retrospective cohort study	Bifurcation lesions	85	DCB (paclitaxel)	Bingo	DES	NA	12 months	12 months	LLL, MACE	Cardiac death, MI, TLR	NA	NA
Hengdao Liu	2022b	China	Retrospective cohort study	Bifurcation lesions	100	DCB (paclitaxel)	Bingo	DES	NA	12 months	6 months	LLL, lumen restenosis	Cardiac death, MI, TLR, angina pectoris	NA	12 months
Yukiko Mizutani	2022	Japan	Retrospective cohort study	ACS	182	DCB (paclitaxel)	Sequent Please	2nd/3rd‐gen DES	Xience Alpine/Ultimaster/Synergy/Xience Sierra/Ultimaster Tansei/Xience Xpedition/Orsiro	671 ± 508 days; 626 ± 543 days	12 months	TLF	Cardiac death, MI, TVR, TLR	6.1	3/6 months
Liang Pan	2022	China	Retrospective cohort study	Bifurcation lesions	597	DCB (paclitaxel)	Sequent Please	DES	NA	24 months	12 months	TLF	Cardiac death, MI, TLR	3.5	NA
Cheng‐Hsuan Tsai	2022	China	Retrospective cohort study	Very small disease	106	DCB (paclitaxel)	SeQuent Please/SeQuent Please Neo/Agent	2nd/3rd‐gen DES	Orsiro/Synergy/Xience Alpine/Resolute Onyx	12 months	NA	TLR, MACCEs	TLR, ACS, stroke, HF‐related admission, and death	NA	NA
Haozhe Dong	2023	China	Retrospective cohort study	Severely calcified coronary lesions	318	DCB (paclitaxel)	Sequent Please	2nd/3rd‐gen DES	Excel/Resolute Integrity/Excrossal/Synergy	15/22 months	NA	TLR, MACCEs	Death, MI, TLR, and stroke	3.5	10/12 months
Naohiro Funayama	2023	Japan	Retrospective cohort study	De novo coronary lesions in dialysis patients	132	DCB (paclitaxel)	Sequent Please	DES	NA	12 months	12 months	TLF	Cardiac death, MI, revascularization	NA	9 months
SPARTAN LMS	2023	United Kingdom	Retrospective cohort study	De novo coronary lesions in LM	148	DCB (paclitaxel)	NA	2nd‐gen DES	NA	33.9 ± 20 months	NA	All‐cause mortality	NA	4.9	NA
Ae‐Young Her	2023	Korea	Retrospective cohort study	De novo coronary lesions	206	DCB (paclitaxel)	Sequent Please	DES	NA	60 months	NA	MACE	Cardiac death, MI, stroke, TLT, TVR	0.0	NA
Ioannis Merinopoulos	2023a	United Kingdom	Retrospective cohort study	De novo coronary lesions	1237	DCB (paclitaxel)	NA	2nd‐gen DES	NA	42 months	NA	All‐cause mortality, MACE	NA	NA	1/12 months
Ioannis Merinopoulos	2023b	United Kingdom	Retrospective cohort study	De novo lesions (STEMI)	1139	DCB (paclitaxel)	NA	2nd‐gen DES	NA	36 months	NA	All‐cause mortality	NA	5.3	12 months
Kentaro Mitsui	2023	Japan	Retrospective cohort study	Severely calcified coronary lesions	135	DCB (paclitaxel)	SeQuent Please	2nd/3rd‐gen DES	Xience/Synergy/Orsiro/Ultimaster	12 months	231/358 days	MACE	Cardiac death, MI, TLR	NA	3/6 months, ACS 3/12 months
Hidehiko Nakamura	2023	Japan	Retrospective cohort study	De novo coronary lesion (large)	154	DCB (paclitaxel)	SeQuent Please	2nd/3rd‐gen DES	Ultimaster/Synergy/Ultimaster/Xience/Promus/Resolute	1536/1343 days	472/399 days	TLF	Cardiac death, MI, TLR, TVR	1.4	NA
Kota Yamada	2023	Japan	Prospective cohort study	De novo coronary lesions	354	DCB (paclitaxel)	SeQuent Please	DES	NA	789/846 days	338/386 days	TLF	Cardiac death, MI, TLR, TVR	NA	NA
X. Cai	2024	China	Retrospective cohort study	De novo coronary lesion (large)	88	DCB (paclitaxel)	SeQuent Please	2nd‐gen DES	Firebird	12 months	9 months	LLL	NA	NA	CCS 1/6 months; ACS 12 months
Jun Goto	2024	Japan	Retrospective cohort study	De novo coronary lesions	337	DCB	NA	DES	NA	12 months	NA	TLF	Cardiac death, MI, TLR, TVR	NA	NA
Ae‐Young Her	2024	Korea	Retrospective cohort study	Bifurcation lesions	82	DCB (paclitaxel)	SeQuent Please	DES	NA	12 months	6 months	*Δ*MLD, *Δ*DS	Cardiac death, MI, stroke, thrombosis, TVR, and major bleeding	0.0	NA

Abbreviations: BMS, bare‐metal stent; DAPT, dual antiplatelet therapy; DCB, drug‐coated balloon; DES, drug‐eluting stent; DoCE, device‐oriented composite endpoint; DS%, diameter stenosis percentage; FFR, fractional flow reserve; LLL, late lumen loss; MACCEs, major adverse cardiac and cerebrovascular events; MACEs, major adverse cardiac events; MI, myocardial infarction; POBA, plain old balloon angioplasty; RCT, randomized controlled trial; TLR, target lesion revascularization; TLS, target lesion stenosis; TLT, target lesion thrombosis; TVF, target vessel failure; TVR, target vessel revascularization.

### 3.3. Primary Endpoints

Regarding the primary safety endpoint, minor variations were observed in the definition of MACE among studies, with detailed descriptions provided in Table 1. When not considering the control group′s intervention, DCB had a lower incidence of MACE compared to other interventional treatments (RR = 0.86, 95% CI: 0.76–0.97). When considering only DES as the control group′s intervention, there was no significant difference in MACE incidence (RR = 0.96, 95% CI: 0.84–1.10). When considering noncoated devices, the DCB group showed a significantly lower incidence of MACE, with statistical significance (RR = 0.52, 95% CI: 0.40–0.69) (Figure 2).

Figure 2Forest plot of MACE and LLL in DCB versus control treatment. Abbreviations: CI, confidence interval; DCB, drug‐coated balloon; DES, drug‐eluting stent; MD, mean difference; RR, risk ratio; SD, standard deviation.

**Figure:**
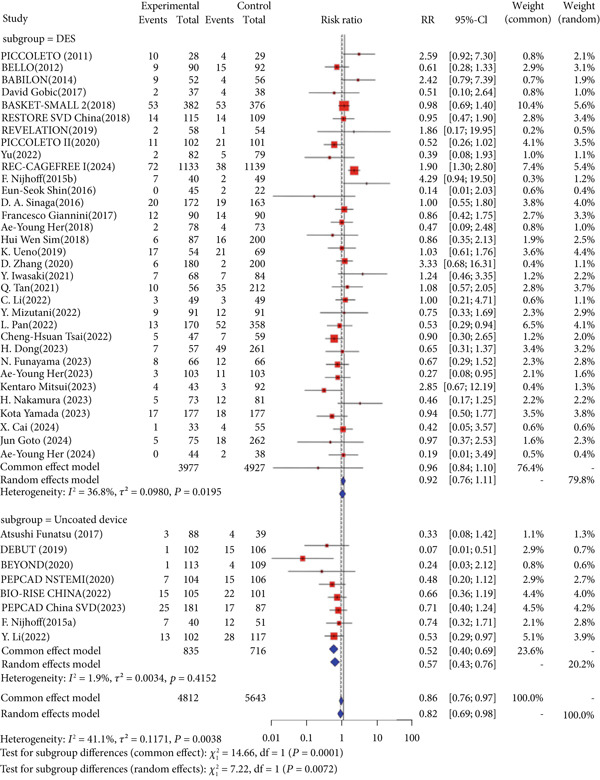
(a) Major adverse cardiac events

**Figure:**
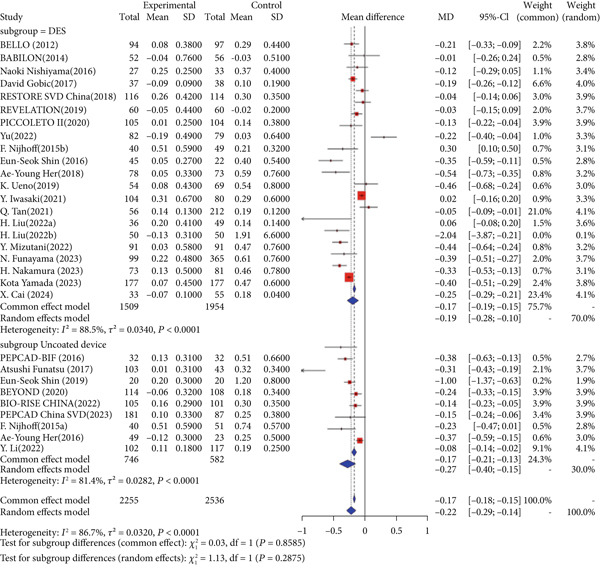
(b) Late lumen loss

For the primary imaging endpoint, a total of 29 studies reported LLL [14, 27–32, 34–36, 38, 40–42, 44–46, 50, 52, 55, 56, 58–61, 65, 71–73]. Regardless of whether the control group was all interventional measures, DES, or noncoated devices, DCB was significantly superior to the control group in terms of LLL. When the control group was all interventional measures (MD = −0.22, 95% CI: −0.29 to −0.14), DES (MD = −0.19, 95% CI: −0.28 to −0.10), or noncoated devices (MD = −0.27, 95% CI: −0.40 to −0.15), DCB showed significant superiority (Figure 2).

Interaction analysis indicated significant heterogeneity between DES and noncoated device subgroups for the primary outcomes. For MACE, the treatment effect differed significantly between subgroups (*p* = 0.0001), while for LLL, no significant interaction was detected (*p* = 0.2875).

### 3.4. TSA

The TSA parameters were set as follows: Type I error 5%, power 80%, and relative risk reduction 20%. For the primary safety outcome MACE, if all studies were included, the required information size was 8699. The curve intersected both the conventional boundary and the TSA boundary, suggesting that the cumulative information size met the required threshold, eliminating the need for additional trials. If only RCTs were included, the required information size was 15,238. The curve failed to intersect both the conventional boundary and the TSA boundary, suggesting that the cumulative information size fell short of the required threshold. This suggests no statistical difference between the intervention and control groups, and approximately 10,000 additional patients would need to be randomized (Figure 3).

Figure 3Trial sequential analysis (TSA) of major adverse cardiac events (MACEs) and late lumen loss (LLL). (a) MACE results from RCTs only; (b) MACE results from RCTs and cohort studies; (c) LLL results from RCTs only; (d) LLL results from RCTs and cohort studies.

**Figure:**
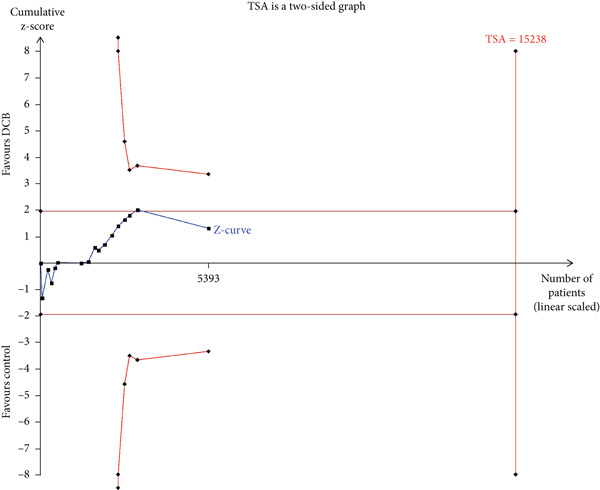
(a)

**Figure:**
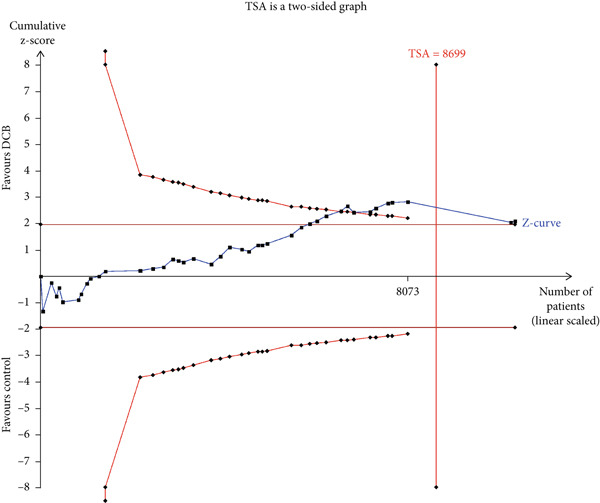
(b)

**Figure:**
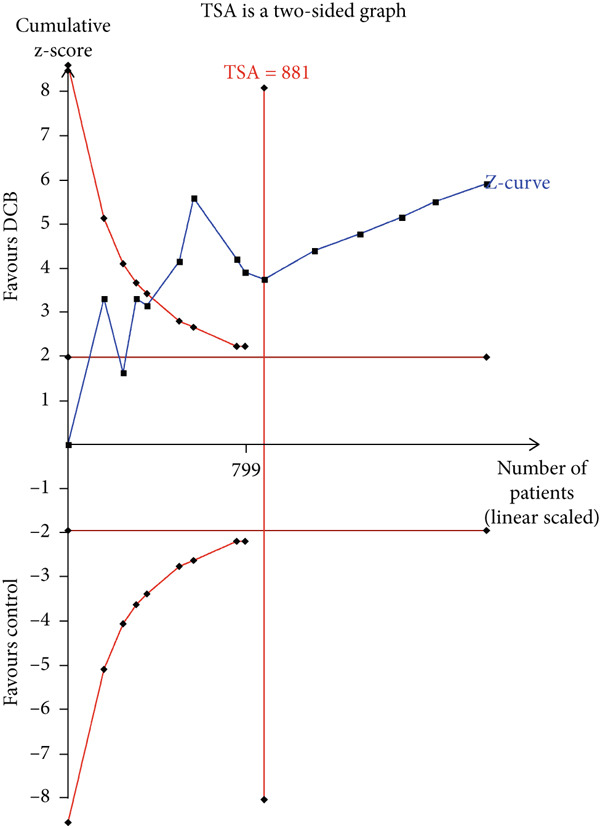
(c)

**Figure:**
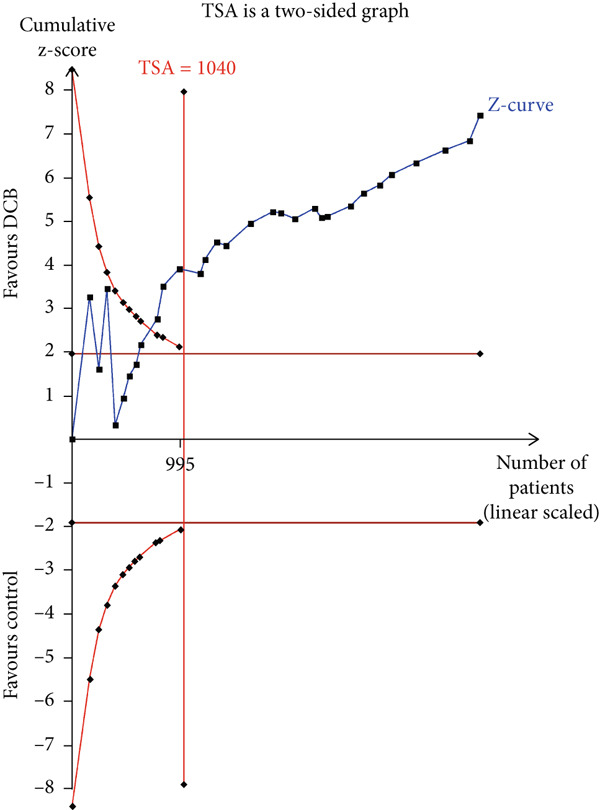
(d)

For the primary imaging outcome LLL, regardless of whether only RCTs or all studies were included, the curve surpassed both the conventional boundary and the TSA boundary. The cumulative information size met the required threshold, suggesting no need for additional trials.

### 3.5. Secondary Endpoints

For secondary safety endpoints, when not screening the control group′s treatment measures, DCB demonstrated no statistically significant differences in TLR (RR = 0.92, 95% CI: 0.76–1.10), MI (RR = 0.98, 95% CI: 0.84–1.14), death (RR = 1.04, 95% CI: 0.92–1.17), cardiac death (RR = 0.89, 95% CI: 0.67–1.18), or TVR (RR = 0.87, 95% CI: 0.60–1.26) (Figure S2).

Compared to DES, the DCB group demonstrated no significant differences in TLR (RR = 1.04, 95% CI: 0.85–1.26), MI (RR = 1.05, 95% CI: 0.89–1.23), death (RR = 1.06, 95% CI: 0.93–1.19), cardiac death (RR = 1.00, 95% CI: 0.74–1.36), or TVR (RR = 0.96, 95% CI: 0.64–1.44) (Figure S2).

Compared to noncoated devices, DCB showed significantly lower risks of TLR (RR = 0.43, 95% CI: 0.26–0.71), MI (RR = 0.32, 95% CI: 0.15–0.67), cardiac death (RR = 0.35, 95% CI: 0.14–0.87), and TVR (RR = 0.51, 95% CI: 0.28–0.95) but no significant difference in death (RR = 0.45, 95% CI: 0.17–1.18) (Figure S2).

For secondary imaging endpoints, when not selecting a control group, DCB showed no statistically significant differences in MLD (MD = −0.08, 95% CI: −0.20 to 0.04) or DS% (MD = −1.31, 95% CI: −5.15 to 2.53). When the control group was DES, DCB demonstrated a notable decrease in MLD (MD = −0.21, 95% CI: −0.34 to −0.09), which was statistically significant. No significant difference was observed in DS% (MD = 2.52, 95% CI: −1.53 to 6.57). When the control group was BMS or POBA, MLD (MD = 0.25, 95% CI: 0.14–0.36) significantly increased, and DS% (MD = −10.73, 95% CI: −13.04 to −8.42) significantly decreased, both with statistical significance (Figure S3).

Interaction analysis showed marked heterogeneity between DES and noncoated device subgroups for several secondary outcomes. Significant differences were observed for MI (*p* = 0.0021), TLR (*p* = 0.0014), cardiac death (*p* = 0.0451), DS% (*p* < 0.0001), and MLD (*p* < 0.0001), whereas no significant interaction was detected for all‐cause death (*p* = 0.0851) or TVR (*p* = 0.1570).

### 3.6. Subgroup Analysis

Given the higher evidence level of RCTs compared to cohort studies, subgroup analyses were performed independently for RCTs and cohort studies, covering both primary and secondary outcomes. Regarding the risk of MACE, RCTs demonstrated no statistically significant difference (RR = 0.80, 95% CI: 0.56–1.12), while cohort studies indicated a reduced risk (RR = 0.80, 95% CI: 0.67–0.94; *p* for interaction = 0.9232). For LLL, both RCTs (MD = −0.18, 95% CI: −0.24 to −0.12) and cohort studies (MD = −0.24, 95% CI: −0.35 to −0.12; *p* for interaction = 0.4207) showed significant reductions (Figure 4).

Figure 4Primary outcomes by study design (RCT vs. cohort). Abbreviations: MACE, major adverse cardiac event; LLL, late lumen loss; RR, risk ratio; MD, mean difference; CI, confidence interval; RCT, randomized controlled trial.

**Figure:**
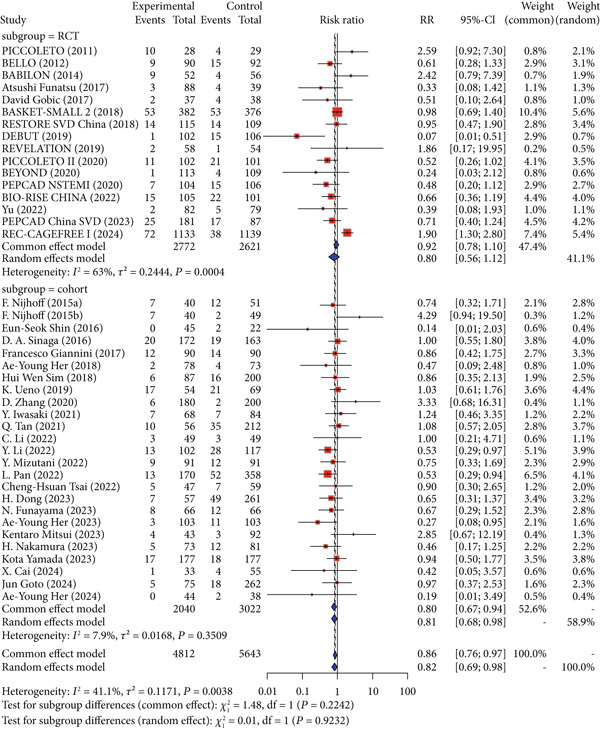
(a) MACE

**Figure:**
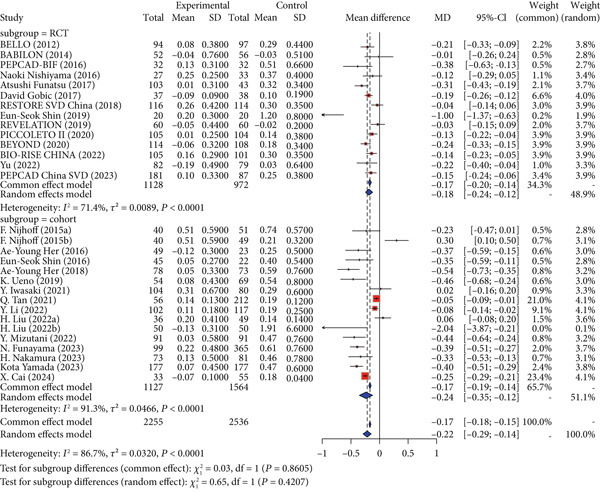
(b) LLL

Regarding secondary safety outcomes, RCTs indicated no significant difference in TLR (RR = 0.84, 95% CI: 0.48–1.50), while the cohort group exhibited a lower risk (RR = 0.77, 95% CI: 0.60–0.98; *p* for interaction = 0.8484). For MI, the RCT group suggested a reduced risk (RR = 0.68, 95% CI: 0.49–0.94), while no significant difference was observed in the cohort group (RR = 1.10, 95% CI: 0.93–1.32; *p* for interaction = 0.0102). For death, the RCT group (RR = 1.11, 95% CI: 0.81–1.53) and the cohort group (RR = 1.03, 95% CI: 0.90–1.17; *p* for interaction = 0.6428) showed no statistically significant differences. For cardiac death, the RCT group (RR = 1.14, 95% CI: 0.76–1.70) and the cohort group (RR = 0.70, 95% CI: 0.46–1.044; *p* for interaction = 0.0912) showed no statistically significant differences. For TVR, no significant difference in incidence was observed in the RCT group (RR = 1.16, 95% CI: 0.63–2.13), while the cohort group showed a reduced incidence (RR = 0.71, 95% CI: 0.52–0.96; *p* for interaction = 0.2025) (Figures S4 and S5).

For secondary imaging outcomes, in terms of MLD, the RCT group (MD = −0.07, 95% CI: −0.24 to 0.10) and the cohort group (MD = −0.09, 95% CI: −0.27 to 0.09) showed no statistically significant differences (*p* for interaction = 0.8879). For DS%, the RCT group (MD = −3.55, 95% CI: −8.10 to 1.01) and the cohort group (MD = 0.47, 95% CI: −5.34 to 6.28) exhibited no significant differences (*p* for interaction = 0.2866) (Figure S6).

All included studies were conducted in Europe or Asia, so the primary outcomes were analyzed by stratifying the European and Asian populations. For the primary safety outcome MACE, no significant differences in incidence were observed in either the Asian population (RR = 0.87, 95% CI: 0.75–1.00) or the European population (RR = 0.87, 95% CI: 0.59–1.29; *p* for interaction = 0.7519). For the primary imaging outcome LLL, the Asian population showed a reduction in LLL (MD = −0.25, 95% CI: −0.34 to −0.17), while no significant difference was observed in the European population (MD = −0.11, 95% CI: −0.24 to 0.02; *p* for interaction = 0.0611) (Figure 5).

Figure 5Primary outcomes by ethnicity. Abbreviations: MACE, major adverse cardiac event; LLL, late lumen loss; RR, risk ratio; MD, mean difference; CI, confidence interval.

**Figure:**
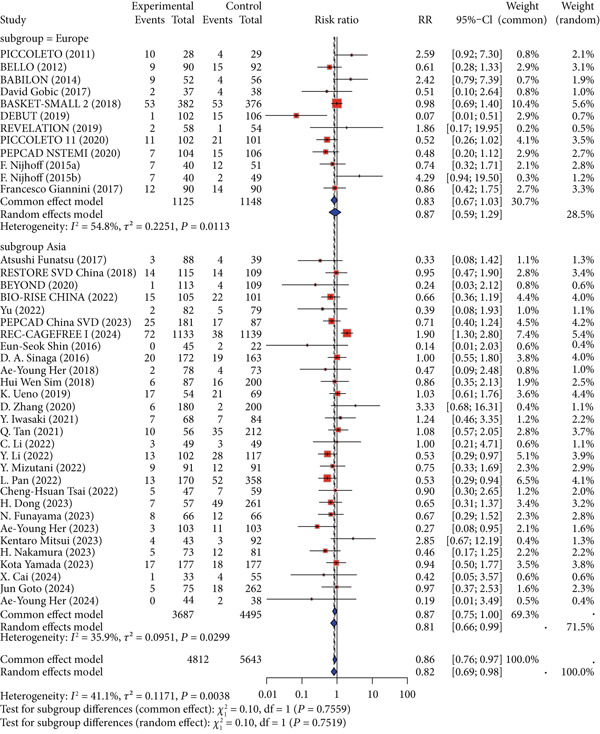
(a) MACE

**Figure:**
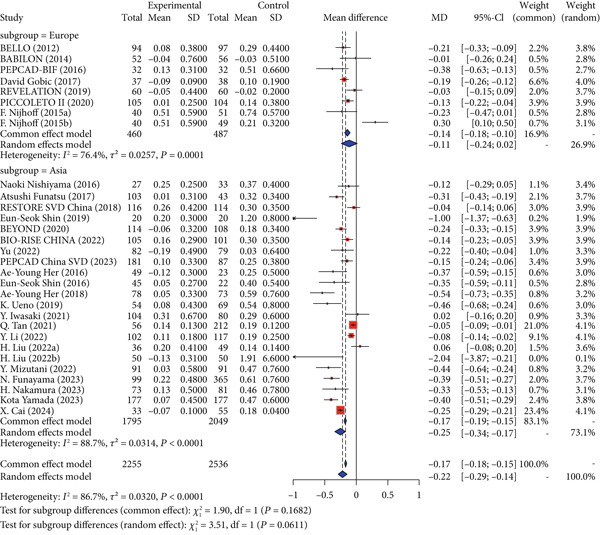
(b) LLL

Using DES as the control group, the primary outcomes of DCB in small vessel disease, ACS lesions, diabetic patients, dual antiplatelet therapy duration, and routine use of intravascular ultrasound (IVUS)/optical coherence tomography (OCT) were discussed. Stratified analysis based on a vessel diameter cutoff of 2.75 mm showed no significant difference in MACE risk between the large vessel group (RR = 0.71, 95% CI: 0.49–1.03) and the small vessel group (RR = 0.92, 95% CI: 0.78–1.10). However, LLL was notably lower in the small vessel group (MD = −0.25, 95% CI: −0.36 to −0.14), whereas no significant difference was observed in the large vessel group (MD = −0.08, 95% CI: −0.24 to 0.07). Interaction analysis showed no significant difference between vessel‐size subgroups for either MACE (*p* = 0.2186) or LLL (*p* = 0.0856).

In ACS lesions, no significant differences were observed in MACE risk (RR = 0.77, 95% CI: 0.44–1.34) or LLL (MD = −0.09, 95% CI: −0.31 to 0.14) with DCB.

Using a 3‐month duration of DAPT in the DCB group CCS patients as the cutoff, the findings revealed no significant difference in MACE risk (RR = 1.09, 95% CI: 0.92–1.30) in the ≤ 3‐month group but a reduction in LLL (MD = −0.21, 95% CI: −0.30 to −0.11). In the > 3‐month group, there were no significant differences in MACE (RR = 0.98, 95% CI: 0.72–1.34) or LLL (MD = −0.10, 95% CI: −0.25 to 0.04). Interaction analysis did not identify significant differences between the two DAPT duration subgroups for either MACE (*p* = 0.5453) or LLL (*p* = 0.2242).

Subgroup analysis based on whether IVUS/OCT was routinely used as guidance during the intervention showed no differences in MACE incidence in either the nonroutine use group (RR = 0.97, 95% CI: 0.83–1.12) or the routine use group (RR = 0.93, 95% CI: 0.68–1.28). For LLL, both the nonroutine use group (MD = −0.15, 95% CI: −0.23 to −0.07) and the routine use group (MD = −0.29, 95% CI: −0.44 to −0.13) showed reductions. Interaction analysis found no significant differences between subgroups for MACE (*p* = 0.8500) or LLL (*p* = 0.1214).

For diabetic patients with de novo CAD, DCB showed no significant difference in MACE risk (RR = 1.21, 95% CI: 0.84–1.75). Due to limitations in the original studies, no studies separately or subgroup‐reported LLL. For bifurcation lesions, no statistically significant differences were observed between DCB and DES in MACE (RR = 0.81, 95% CI: 0.23–2.92) or LLL (MD = −0.02, 95% CI: −0.30 to 0.26) (Figure 6).

Figure 6Effect estimates (with 95% confidence intervals) pooled for DCB compared to DES in subgroup evaluations. Abbreviation: RR, risk ratio; CI, confidence interval; MD, mean difference; RVD, reference vessel diameter; DAPT, dual antiplatelet therapy; IVUS, intravascular ultrasound; OCT, optical coherence tomography.

**Figure:**
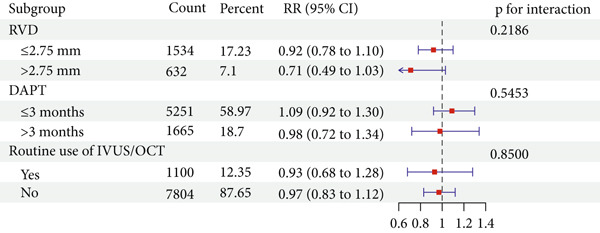
(a) MACE

**Figure:**
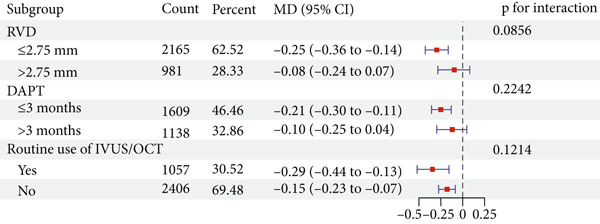
(b) LLL

The subgroup analysis of DCB versus DES is detailed in Figures S7, S8, S9, S10, S11, and S12.

### 3.7. Risk of Bias and Sensitivity Analysis

Figure S1 and Table S3 provide a concise summary of the risk of bias assessment for each study included in the analysis. On the whole, the majority of the studies exhibited a high level of quality, with no notable biases identified. To assess publication bias across all endpoints, funnel plots were utilized. Upon visual examination, it was observed that the funnel plots displayed a general symmetry. Egger′s test (*p* = 0.0745) and Begg′s test (*p* = 0.3454) for the primary safety outcome, and Egger′s test (*p* = 0.1189) and Begg′s test (*p* = 0.1928) for the primary imaging outcome, showed no significant bias (Figure S13).

To evaluate the robustness of the meta‐analysis results, a sensitivity analysis employing the leave‐one‐out method was performed for the primary endpoints. In this process, each study was individually excluded in sequence, followed by a reassessment of the pooled effect size and its corresponding 95% CI. The results showed that the 95% CI did not significantly shift after excluding any study (Figure S14).

Under this fixed MACE definition, the overall pooled RR for MACE was 0.75 (95% CI 0.38–1.48), consistent with the main analysis. Compared with DES, DCBs showed no significant difference in MACE incidence (RR = 1.01, 95% CI 0.52–1.96). In contrast, compared with noncoated devices, DCBs demonstrated a significantly lower MACE risk (RR = 0.17, 95% CI 0.03–0.90; *p* for interaction = 0.0513). When stratified by study design, both RCTs (RR = 0.60, 95% CI 0.20–1.76) and cohort studies (RR = 0.88, 95% CI 0.39–1.99) showed nonsignificant differences, with no significant interaction between study designs (*p* for interaction = 0.5748). Stratification by population region yielded similar results across Asian (RR = 0.98, 95% CI 0.46–2.08) and European studies (RR = 0.40, 95% CI 0.09–1.87), with no significant subgroup differences (*p* = 0.3068) (Figures S15, S16, and S17).

## 4. Discussion

DCBs have exhibited both safety and effectiveness in the management of ISR lesions, earning recommendations as a therapeutic option for ISR in numerous guidelines and expert consensus statements, with a recommendation level of IA [5, 10, 76]. However, there is no unified conclusion regarding their use in de novo CAD and their clinical comorbidities.

In patients with de novo CAD, DCB showed safety outcomes comparable to DES, supporting its potential as an alternative treatment option. In terms of angiographic efficacy, DCB achieved lower LLL but smaller MLD, with no significant difference in DS%, indicating overall comparable angiographic results. When compared with POBA or BMS, DCB demonstrated a clear advantage in the primary clinical endpoint of MACE, with lower incidences of MI, cardiac death, TLR, and TVR, while all‐cause mortality remained similar. DCB also showed superior angiographic outcomes, reinforcing its favorable safety and efficacy relative to conventional balloon angioplasty.

Interaction analyses indicated that treatment effects varied according to the comparator device. The differences were mainly observed in ischemic outcomes such as MACE, MI, TLR, and cardiac death, where the benefit of DCB appeared more pronounced against noncoated devices than against DES. In contrast, all‐cause mortality and TVR were largely consistent across comparator types. For angiographic outcomes, significant interactions were also found for DS% and MLD, whereas LLL showed no meaningful difference between subgroups. Taken together, these results suggest that part of the observed heterogeneity among studies may be explained by differences in comparator devices. Nevertheless, the overall evidence supports the favorable safety and angiographic performance of DCB as a potential alternative for the treatment of de novo CAD, particularly in patients for whom avoiding permanent stent implantation is desirable [77].

In the main analysis, observational cohort studies suggested that DCB use was associated with a lower risk of MACE, whereas RCTs did not show a significant difference compared with stent‐based strategies. This discrepancy may be explained by methodological and clinical factors. Observational cohorts often reflect real‐world practice, where DCBs are selectively applied to less complex or well‐prepared lesions, introducing potential selection bias even after statistical adjustment [66]. In contrast, RCTs employ predefined inclusion criteria, standardized lesion preparation, and protocolized follow‐up, which typically yield more conservative estimates of treatment effect [12, 14].

Variation in the definition of MACE may also have contributed to the observed inconsistency. Several included studies used broader composite endpoints, including target vessel revascularization, unstable angina, or stroke [49, 54, 58, 64], instead of limiting MACE to cardiovascular death, MI, and TLR. Such heterogeneity can increase event rates and obscure genuine differences between treatment groups.

After restricting the analysis to studies adopting a standardized MACE definition, both RCTs and cohort studies yielded similar, nonsignificant results. The pooled estimates were consistent with the main analysis across study design and regional subgroups, showing no material change in direction or statistical significance. DCBs demonstrated comparable MACE incidence to DES and a numerically lower risk compared with noncoated devices, with the interaction between comparator type and treatment effect reaching borderline significance (*p* for interaction = 0.0513). These findings suggest that discrepancies between RCTs and observational studies were largely driven by inconsistent endpoint definitions rather than by fundamental differences in treatment effects. Implementing uniform endpoint criteria can therefore reduce between‐study heterogeneity and improve the comparability and interpretability of DCB outcomes across diverse study populations [76, 78, 79].

TSA results indicated that the current meta‐analysis findings on the primary safety outcome MACE are highly reliable. The Z‐curve trajectory surpassed the efficacy threshold, demonstrating that the current evidence is sufficient to support the conclusion on MACE incidence. However, if only RCTs were considered, the statistical trajectory failed to reach any thresholds, suggesting that further validation studies are required to confirm the observed outcomes. Consequently, these preliminary findings should be interpreted with appropriate scientific prudence and methodological reservation. Regarding the primary imaging endpoint of LLL, the meta‐analysis demonstrated robust reliability. The Z‐curve trajectory surpassed the efficacy threshold, including both the RCT‐only analysis and all‐studies approach, thereby confirming the adequacy of evidentiary support.

Due to differences in risk factors and prognosis for CAD between Asian and European populations [80], we analyzed these populations separately. The results showed that both Asian and European populations had similar safety outcomes, with no significant differences compared to the control group. Although Asian studies showed a trend toward greater improvement in imaging outcomes, this difference did not reach statistical significance when formally tested for interaction (*p* for interaction = 0.9201 for MACE and 0.0581 for LLL), suggesting that the overall treatment effect of DCB was largely consistent across populations despite baseline risk factor disparities.

Approximately 40% of CAD patients have diabetes [81, 82]. Considering the substantial prevalence of diabetes among these patients and its pathophysiological contributions to atherosclerotic progression and adverse cardiovascular outcomes [83], we separately discussed the safety of DCB compared to DES in diabetic patients. For the primary clinical outcome MACE, there were no significant differences between the two treatments. However, since the original studies did not separately report or subgroup‐analyze LLL in diabetic populations, we were unable to assess LLL differences between the groups. Future studies should focus on this specific population.

In ACS patients, DCB showed similar safety to DES. Statistically insignificant variations were noted in the key imaging endpoint, which was LLL. The primary safety outcomes were consistent with the main study, but the imaging outcomes were slightly worse in ACS patients. These findings indicate that DCB may represent a viable therapeutic alternative for ACS patients, though caution is warranted. In the analysis stratified by vessel diameter, both groups demonstrated favorable safety profiles regardless of vessel size. However, the small vessel group exhibited superior efficacy relative to the large vessel group. In the management of bifurcation lesions, no notable intergroup variations were detected between DCBs and DESs with regard to the primary endpoints. Furthermore, the investigation unveiled that the routine application of IVUS or OCT did not exert a significant impact on the safety and effectiveness of DCBs, and DAPT for more than 3 months did not significantly affect DCB safety but reduced its advantage in LLL.

Subgroup analysis based on study design showed inconsistent results between RCTs and cohort studies. Given the higher evidence level of RCTs, we tend to rely on RCT results and interpret them cautiously. The results indicate that DCB has comparable safety to other interventional measures and superior efficacy. Interaction analyses further demonstrated no significant heterogeneity for most endpoints, suggesting broadly consistent effects of DCB across both randomized and observational settings. The only exception was observed for MI, where the treatment effect differed modestly between RCTs and cohort studies (*p* for interaction = 0.0102), possibly reflecting variations in event adjudication and follow‐up intensity.

Previous studies have shown that thicker stents are correlated with a heightened probability of thrombosis [84]. The latest generation of DES has reduced stent thickness from 80–100 *μ*m to less than 70 *μ*m, and a meta‐analysis has shown that newer‐generation DES further improves 1‐year clinical outcomes [85]. Since most control groups in the included studies used second‐generation DES and only a narrow range of studies used newer‐generation DES, future studies should assess the comparative safety and clinical efficacy of DCB with newer‐generation DES as they become more prevalent.

While coronary stents continue to evolve, DCBs are also advancing. Paclitaxel‐coated devices have been criticized for their biotoxicity, and a meta‐analysis by Katsanos showed that paclitaxel‐coated devices increase mortality [86]. First‐generation DESs with paclitaxel coatings have been phased out, and second‐generation DESs now predominantly use rapamycin and its derivatives (e.g., everolimus, zotarolimus, and biolimus). A meta‐analysis showed that rapamycin and its derivatives have similar clinical safety outcomes to paclitaxel‐coated balloons but offer better late imaging outcomes [86]. Sirolimus‐coated balloons have demonstrated encouraging outcomes in preclinical studies using animal subjects [87]. However, within the primary research investigations incorporated in this meta‐analysis, only one used a sirolimus‐coated balloon, and the control group was POBA, preventing comparison with DES or paclitaxel‐coated DCBs [41]. The SELUTION DE NOVO trial, comparing sirolimus‐coated balloons with limus‐eluting stents, is ongoing [88]. Beyond sirolimus or paclitaxel as single coatings, research by Kawai [89] has shown that DCBs coated with a novel dual‐active drug combination (synergistic combination of paclitaxel and sirolimus) exhibit similar antiproliferative effects while reducing total drug exposure, potentially offering better clinical outcomes and safety. Future studies should incorporate these novel DCBs and compare them with first‐line treatments to explore the best interventional approaches.

## 5. Limitations

In the TSA for MACE, if only RCTs were included, the sample size did not attain the anticipated threshold, suggesting that the safety profile of DCB necessitates meticulous assessment, and caution is warranted. In the studies encompassed by this meta‐analysis, the PICCOLETO study reported bailout stenting in 34.5% of the DCB group [22], the BELLO study reported 20.2% [27], and some studies did not report bailout stenting rates. Elevated rates of bailout stenting could potentially compromise the accurate assessment of DCB′s safety and efficacy profiles.

Moreover, this meta‐analysis included both RCTs and cohort studies, which introduce limitations. First, there are significant methodological differences between RCTs and cohort studies. RCTs generally have higher internal validity, while cohort studies, even with propensity score matching (PSM), may still be influenced by confounding factors, potentially affecting the interpretation of results. The risk of bias varies between RCTs and cohort studies. While the Cochrane Risk of Bias Tool is used to evaluate RCTs and the NOS is applied to cohort studies, it is recognized that cohort studies inherently possess a higher potential for bias in comparison to RCTs. Therefore, the conclusions of this study should be interpreted cautiously.

In addition, heterogeneity in MACE definitions across studies may have affected the pooled estimates. Although the sensitivity analysis using a standardized MACE definition produced results largely consistent with the main findings, minor variations in endpoint composition across studies could not be completely eliminated and may have introduced residual bias.

## 6. Conclusion

In de novo CAD, DCB shows comparable safety to DES, with no significant differences in all safety outcomes (MACE, death, cardiac death, MI, TLR, and TVR). In imaging outcomes, DCB is largely similar to DES (superior in LLL, inferior in MLD, and no significant difference in DS%). Therefore, DCB is a reliable alternative to DES, offering good safety and efficacy. DCB also demonstrates reliable safety and efficacy in special populations, such as individuals with diabetes, ACS, or small vessel disease, as well as across different ethnicities and in bifurcation lesions. Notably, the therapeutic benefits were more pronounced in small vessels. The routine use of IVUS/OCT showed no significant impact, and prolonged dual antiplatelet therapy may reduce DCB′s advantage in LLL. Compared to noncoated devices (BMS/POBA), DCB is superior in all aspects except mortality, making it a viable alternative in clinical practice.

However, the TSA for MACE in RCTs indicated that the sample size was insufficient, and the cumulative curve did not cross the TSA boundary, highlighting the need for additional large‐scale, long‐term studies incorporating new‐generation balloons and stents to confirm these findings. Additionally, since this study included both RCTs and cohort studies, the effectiveness and safety of DCB should not be overestimated, and findings should be interpreted with caution.

## Conflicts of Interest

The authors declare no conflicts of interest.

## Funding

This study received funding from Noncommunicable Chronic Diseases—National Science and Technology Major Project (2023ZD0504100).

## Supporting information


**Supporting Information** Additional supporting information can be found online in the Supporting Information section. Table S1: Full search strategy and search terms. Table S2: Patient and disease characteristics in eligible studies. Table S3: Target lesion characteristics. Figure S1: Bias assessment of the included RCTs according to the Cochrane Collaboration. Figure S2: Forest plot of risk ratios for secondary clinical outcomes. Figure S3: Forest plot of mean differences (MDs) for secondary imaging outcomes. Figure S4: Forest plot of secondary clinical outcomes in RCTs and cohort studies. Figure S5: Forest plot of secondary clinical outcomes in RCTs and cohort studies. Figure S6: Forest plot of secondary imaging outcomes in RCTs and cohort studies. Figure S7: Forest plot of primary outcomes stratified by vessel diameter. Figure S8: Forest plot of primary outcomes stratified by DAPT. Figure S9: Forest plot of primary outcomes according to the routine use of intravascular imaging techniques. Figure S10: Forest plot of primary outcomes in patients with ACS. Figure S11: Forest plot of MACE in patients with diabetes mellitus. Figure S12: Forest plot of primary outcomes in bifurcation lesions. Figure S13: Funnel plot for publication bias of studies included in the meta‐analysis. Figure S14: Sensitivity analysis. Figure S15: Forest plot for the sensitivity analysis using standardized MACE stratified by comparator category (DCB vs. uncoated device and DCB vs. DES). Figure S16: Forest plot for the sensitivity analysis using standardized MACE stratified by study design (RCT vs. cohort). Figure S17: Forest plot for the sensitivity analysis using standardized MACE stratified by region (Asia vs. Europe).

## Data Availability

The data that support the findings of this study are available from the corresponding author upon reasonable request.

## References

[1] Zhao D. , Liu J. , Wang M. , Zhang X. , and Zhou M. , Epidemiology of Cardiovascular Disease in China: Current Features and Implications, Nature Reviews. Cardiology. (2019) 16, no. 4, 203–212, 10.1038/s41569-018-0119-4, 2-s2.0-85057117993, 30467329. 30467329

[2] Roth G. A. , Mensah G. A. , Johnson C. O. , Addolorato G. , Ammirati E. , Baddour L. M. , Barengo N. C. , Beaton A. Z. , Benjamin E. J. , Benziger C. P. , Bonny A. , Brauer M. , Brodmann M. , Cahill T. J. , Carapetis J. , Catapano A. L. , Chugh S. S. , Cooper L. T. , Coresh J. , Criqui M. , DeCleene N. , Eagle K. A. , Emmons-Bell S. , Feigin V. L. , Fernandez-Sola J. , Fowkes G. , Gakidou E. , Grundy S. M. , He F. J. , Howard G. , Hu F. , Inker L. , Karthikeyan G. , Kassebaum N. , Koroshetz W. , Lavie C. , Lloyd-Jones D. , Lu H. S. , Mirijello A. , Temesgen A. M. , Mokdad A. , Moran A. E. , Muntner P. , Narula J. , Neal B. , Ntsekhe M. , Moraes de Oliveira G. , Otto C. , Owolabi M. , Pratt M. , Rajagopalan S. , Reitsma M. , Ribeiro A. L. P. , Rigotti N. , Rodgers A. , Sable C. , Shakil S. , Sliwa-Hahnle K. , Stark B. , Sundstrom J. , Timpel P. , Tleyjeh I. M. , Valgimigli M. , Vos T. , Whelton P. K. , Yacoub M. , Zuhlke L. , Murray C. , Fuster V. , and Group G-N-JGBoCDW , Global Burden of Cardiovascular Diseases and Risk Factors, 1990–2019, Journal of the American College of Cardiology. (2020) 76, no. 25, 2982–3021, 10.1016/j.jacc.2020.11.010. 33309175 PMC7755038

[3] Martin S. S. , Aday A. W. , Almarzooq Z. I. , Anderson C. A. M. , Arora P. , Avery C. L. , Baker-Smith C. M. , Barone Gibbs B. , Beaton A. Z. , Boehme A. K. , Commodore-Mensah Y. , Currie M. E. , Elkind M. S. V. , Evenson K. R. , Generoso G. , Heard D. G. , Hiremath S. , Johansen M. C. , Kalani R. , Kazi D. S. , Ko D. , Liu J. , Magnani J. W. , Michos E. D. , Mussolino M. E. , Navaneethan S. D. , Parikh N. I. , Perman S. M. , Poudel R. , Rezk-Hanna M. , Roth G. A. , Shah N. S. , St-Onge M. P. , Thacker E. L. , Tsao C. W. , Urbut S. M. , Van Spall H. G. C. , Voeks J. H. , Wang N. Y. , Wong N. D. , Wong S. S. , Yaffe K. , Palaniappan L. P. , American Heart Association Council on E , Prevention Statistics C , and Stroke Statistics S , 2024 Heart Disease and Stroke Statistics: A Report of US and Global Data From the American Heart Association, Circulation. (2024) 149, no. 8, e347–e913, 10.1161/CIR.0000000000001209. 38264914 PMC12146881

[4] Ludman P. F. , Percutaneous Coronary Intervention, Medicine. (2018) 46, no. 9, 547–554, 10.1016/j.mpmed.2018.06.007, 2-s2.0-85050379482.

[5] Neumann F. J. , Sousa-Uva M. , Ahlsson A. , Alfonso F. , Banning A. P. , Benedetto U. , Byrne R. A. , Collet J. P. , Falk V. , Head S. J. , Juni P. , Kastrati A. , Koller A. , Kristensen S. D. , Niebauer J. , Richter D. J. , Seferovic P. M. , Sibbing D. , Stefanini G. G. , Windecker S. , Yadav R. , Zembala M. O. , and Group ESCSD , 2018 ESC/EACTS Guidelines on Myocardial Revascularization, European Heart Journal. (2019) 40, no. 2, 87–165, 10.1093/eurheartj/ehy394, 2-s2.0-85058314412, 30165437. 30165437

[6] Inoue T. , Croce K. , Morooka T. , Sakuma M. , Node K. , and Simon D. I. , Vascular Inflammation and Repair, JACC. Cardiovascular Interventions. (2011) 4, no. 10, 1057–1066, 10.1016/j.jcin.2011.05.025, 2-s2.0-80054993002. 22017929 PMC3341937

[7] Ochijewicz D. , Tomaniak M. , Opolski G. , and Kochman J. , Inflammation as a Determinant of Healing Response After Coronary Stent Implantation, The International Journal of Cardiovascular Imaging. (2021) 37, no. 3, 791–801, 10.1007/s10554-020-02073-3, 33479786. 33479786 PMC7969567

[8] Kuramitsu S. , Sonoda S. , Ando K. , Otake H. , Natsuaki M. , Anai R. , Honda Y. , Kadota K. , Kobayashi Y. , and Kimura T. , Drug-Eluting Stent Thrombosis: Current and Future Perspectives, Cardiovascular Intervention and Therapeutics. (2021) 36, no. 2, 158–168, 10.1007/s12928-021-00754-x, 33439454. 33439454

[9] Joner M. , Finn A. V. , Farb A. , Mont E. K. , Kolodgie F. D. , Ladich E. , Kutys R. , Skorija K. , Gold H. K. , and Virmani R. , Pathology of Drug-Eluting Stents in Humans, Journal of the American College of Cardiology. (2006) 48, no. 1, 193–202, 10.1016/j.jacc.2006.03.042, 2-s2.0-33745233024. 16814667

[10] Yerasi C. , Case B. C. , Forrestal B. J. , Torguson R. , Weintraub W. S. , Garcia-Garcia H. M. , and Waksman R. , Drug-Coated Balloon for De Novo Coronary Artery Disease: JACC State-of-the-Art Review, Journal of the American College of Cardiology. (2020) 75, no. 9, 1061–1073, 10.1016/j.jacc.2019.12.046, 32138967. 32138967

[11] Moher D. , Liberati A. , Tetzlaff J. , Altman D. G. , and Group P , Preferred Reporting Items for Systematic Reviews and Meta-Analyses: The PRISMA Statement, Journal of Clinical Epidemiology. (2009) 62, no. 10, 1006–1012, 10.1016/j.jclinepi.2009.06.005, 2-s2.0-84925548115. 19631508

[12] Gao C. , He X. , Ouyang F. , Zhang Z. , Shen G. , Wu M. , Yang P. , Ma L. , Yang F. , Ji Z. , Wang H. , Wu Y. , Fang Z. , Jiang H. , Wen S. , Liu Y. , Li F. , Zhou J. , Zhu B. , Liu Y. , Zhang R. , Zhang T. , Wang P. , Liu J. , Jiang Z. , Xia J. , van Geuns R. J. , Capodanno D. , Garg S. , Onuma Y. , Wang D. , Serruys P. W. , Tao L. , and Investigators R.-C. I. , Drug-Coated Balloon Angioplasty With Rescue Stenting Versus Intended Stenting for the Treatment of Patients With De Novo Coronary Artery Lesions (REC-CAGEFREE I): An Open-Label, Randomised, Non-Inferiority Trial, Lancet. (2024) 404, no. 10457, 1040–1050, 10.1016/S0140-6736(24)01594-0, 39236727. 39236727

[13] Rissanen T. T. , Uskela S. , Eranen J. , Mantyla P. , Olli A. , Romppanen H. , Siljander A. , Pietila M. , Minkkinen M. J. , Tervo J. , Karkkainen J. M. , Vatanen A. , Perala A. , Muller M. , Porela P. , Palojoki E. , and Investigators D. T. , Drug-Coated Balloon for Treatment of De-Novo Coronary Artery Lesions in Patients With High Bleeding Risk (DEBUT): A Single-Blind, Randomised, Non-Inferiority Trial, Lancet. (2019) 394, no. 10194, 230–239, 10.1016/S0140-6736(19)31126-2, 2-s2.0-85068567978. 31204115

[14] Cortese B. , Di Palma G. , Guimaraes M. G. , Piraino D. , Orrego P. S. , Buccheri D. , Rivero F. , Perotto A. , Zambelli G. , and Alfonso F. , Drug-Coated Balloon Versus Drug-Eluting Stent for Small Coronary Vessel Disease, JACC: Cardiovascular Interventions. (2020) 13, no. 24, 2840–2849, 10.1016/j.jcin.2020.08.035. 33248978

[15] Herzog R. , Alvarez-Pasquin M. J. , Diaz C. , Del Barrio J. L. , Estrada J. M. , and Gil A. , Are Healthcare Workers′ Intentions to Vaccinate Related to Their Knowledge, Beliefs and Attitudes? A Systematic Review, BMC Public Health. (2013) 13, no. 1, 10.1186/1471-2458-13-154, 2-s2.0-84873975790, 23421987. PMC3602084 23421987

[16] Team RC , R: A Language and Environment for Statistical Computing, 2022, R Foundation for Statistical Computing.

[17] Wan X. , Wang W. Q. , Liu J. M. , and Tong T. J. , Estimating the Sample Mean and Standard Deviation From the Sample Size, Median, Range and/or Interquartile Range, Methodology. (2014) 14, no. 1, 10.1186/1471-2288-14-135, 2-s2.0-84926434654. PMC4383202 25524443

[18] Schwarzer G. , Carpenter J. , and Rücker G. , Meta-Analysis With R, 2015, 10.1007/978-3-319-21416-0.

[19] Luo D. H. , Wan X. , Liu J. M. , and Tong T. J. , Optimally Estimating the Sample Mean From the Sample Size, Median, Mid-Range, and/or Mid-Quartile Range, Statistical Methods in Medical Research. (2018) 27, no. 6, 1785–1805, 10.1177/0962280216669183, 2-s2.0-85042003325, 27683581. 27683581

[20] Shi J. D. , Luo D. H. , Wan X. , Liu Y. , Liu J. M. , Bian Z. X. , and Tong T. J. , Detecting the Skewness of Data From the Five-Number Summary and Its Application in Meta-Analysis, Statistical Methods in Medical Research. (2023) 32, no. 7, 1338–1360, 10.1177/09622802231172043, 37161735. 37161735

[21] McGrath S. , Zhao X. F. , Steele R. , Thombs B. D. , Benedetti A. , Levis B. , Riehm K. E. , Saadat N. , Levis A. W. , Azar M. , Rice D. B. , Sun Y. , Krishnan A. , He C. , Wu Y. , Bhandari P. M. , Neupane D. , Imran M. , Boruff J. , Cuijpers P. , Gilbody S. , Ioannidis J. P. A. , Kloda L. A. , McMillan D. , Patten S. B. , Shrier I. , Ziegelstein R. C. , Akena D. H. , Arroll B. , Ayalon L. , Baradaran H. R. , Baron M. , Beraldi A. , Bombardier C. H. , Butterworth P. , Carter G. , Chagas M. H. , Chan J. C. N. , Cholera R. , Chowdhary N. , Clover K. , Conwell Y. , de Man-van Ginkel J. M. , Delgadillo J. , Fann J. R. , Fischer F. H. , Fischler B. , Fung D. , Gelaye B. , Goodyear-Smith F. , Greeno C. G. , Hall B. J. , Harrison P. A. , Harter M. , Hegerl U. , Hides L. , Hobfoll S. E. , Hudson M. , Hyphantis T. , Inagaki M. , Ismail K. , Jetté N. , Khamseh M. E. , Kiely K. M. , Kwan Y. , Lamers F. , Liu S. I. , Lotrakul M. , Loureiro S. R. , Löwe B. , Marsh L. , McGuire A. , Sidik S. M. , Munhoz T. N. , Muramatsu K. , Osório F. L. , Patel V. , Pence B. W. , Persoons P. , Picardi A. , Reuter K. , Rooney A. G. , Santos I. S. , Shaaban J. , Sidebottom A. , Simning A. , Stafford L. , Sung S. C. , Tan P. L. L. , Turner A. , van der Feltz-Cornelis C. M. , van Weert H. C. , Vöhringer P. A. , White J. , Whooley M. A. , Winkley K. , Yamada M. , Zhang Y. Y. , and DEPRESSD DESD , Estimating the Sample Mean and Standard Deviation From Commonly Reported Quantiles in Meta-Analysis, Statistical Methods in Medical Research. (2020) 29, no. 9, 2520–2537, 10.1177/0962280219889080, 32292115. 32292115 PMC7390706

[22] Cortese B. , The PICCOLETO Study and Beyond, Euro Intervention. (2011) 7, no. Supplement K, K53–K56, 10.4244/EIJV7SKA9, 2-s2.0-80355128285. 22027728

[23] Brok J. , Thorlund K. , Gluud C. , and Wetterslev J. , Trial Sequential Analysis Reveals Insufficient Information Size and Potentially False Positive Results in Many Meta-Analyses, Journal of Clinical Epidemiology. (2008) 61, no. 8, 763–769, 10.1016/j.jclinepi.2007.10.007, 2-s2.0-45949100256, 18411040. 18411040

[24] Wetterslev J. , Thorlund K. , Brok J. , and Gluud C. , Trial Sequential Analysis May Establish When Firm Evidence Is Reached in Cumulative Meta-Analysis, Journal of Clinical Epidemiology. (2008) 61, no. 1, 64–75, 10.1016/j.jclinepi.2007.03.013, 2-s2.0-37049035730, 18083463. 18083463

[25] Wetterslev J. , Jakobsen J. C. , and Gluud C. , Trial Sequential Analysis in Systematic Reviews With Meta-Analysis, BMC Medical Research Methodology. (2017) 17, no. 1, 10.1186/s12874-017-0315-7, 2-s2.0-85014688174. PMC5397700 28264661

[26] Higgins J. , Thompson S. , Deeks J. , and Altman D. , Statistical Heterogeneity in Systematic Reviews of Clinical Trials: A Critical Appraisal of Guidelines and Practice, Journal of Health Services Research & Policy. (2002) 7, no. 1, 51–61, 10.1258/1355819021927674, 2-s2.0-0036139581, 11822262. 11822262

[27] Latib A. , Colombo A. , Castriota F. , Micari A. , Cremonesi A. , De Felice F. , Marchese A. , Tespili M. , Presbitero P. , Sgueglia G. A. , Buffoli F. , Tamburino C. , Varbella F. , and Menozzi A. , A Randomized Multicenter Study Comparing a Paclitaxel Drug-Eluting Balloon With a Paclitaxel-Eluting Stent in Small Coronary Vessels: The BELLO (Balloon Elution and Late Loss Optimization) Study, Journal of the American College of Cardiology. (2012) 60, no. 24, 2473–2480, 10.1016/j.jacc.2012.09.020, 2-s2.0-84871342561, 23158530. 23158530

[28] Lopez Minguez J. R. , Nogales Asensio J. M. , Doncel Vecino L. J. , Sandoval J. , Romany S. , Martinez Romero P. , Fernandez Diaz J. A. , Fernandez Portales J. , Gonzalez Fernandez R. , Martinez Caceres G. , Merchan Herrera A. , Alfonso Manterola F. , and Investigators B. , A Prospective Randomised Study of the Paclitaxel-Coated Balloon Catheter in Bifurcated Coronary Lesions (BABILON Trial): 24-Month Clinical and Angiographic Results, EuroIntervention. (2014) 10, no. 1, 50–57, 10.4244/EIJV10I1A10, 2-s2.0-84902305799, 24832638. 24832638

[29] Kleber F. X. , Rittger H. , Ludwig J. , Schulz A. , Mathey D. G. , Boxberger M. , Degenhardt R. , Scheller B. , and Strasser R. H. , Drug Eluting Balloons as Stand Alone Procedure for Coronary Bifurcational Lesions: Results of the Randomized Multicenter PEPCAD-BIF Trial, Clinical Research in Cardiology. (2016) 105, no. 7, 613–621, 10.1007/s00392-015-0957-6, 2-s2.0-84954325567, 26768146. 26768146

[30] Nishiyama N. , Komatsu T. , Kuroyanagi T. , Fujikake A. , Komatsu S. , Nakamura H. , Yamada K. , Nakahara S. , Kobayashi S. , and Taguchi I. , Clinical Value of Drug-Coated Balloon Angioplasty for De Novo Lesions in Patients With Coronary Artery Disease, International Journal of Cardiology. (2016) 222, 113–118, 10.1016/j.ijcard.2016.07.156, 2-s2.0-84979955504. 27494722

[31] Funatsu A. , Nakamura S. , Inoue N. , Nanto S. , Nakamura M. , Iwabuchi M. , Ando K. , Asano R. , Habara S. , Saito S. , Kozuma K. , and Mitsudo K. , A Multicenter Randomized Comparison of Paclitaxel-Coated Balloon With Plain Balloon Angioplasty in Patients With Small Vessel Disease, Clinical Research in Cardiology. (2017) 106, no. 10, 824–832, 10.1007/s00392-017-1126-x, 2-s2.0-85020299205, 28589231. 28589231

[32] Gobić D. , Tomulić V. , Lulić D. , Židan D. , Brusich S. , Jakljević T. , and Zaputović L. , Drug-Coated Balloon Versus Drug-Eluting Stent in Primary Percutaneous Coronary Intervention: A Feasibility Study, American Journal of the Medical Sciences. (2017) 354, no. 6, 553–560, 10.1016/j.amjms.2017.07.005, 2-s2.0-85038130008. 29208251

[33] Jeger R. V. , Farah A. , Ohlow M.-A. , Mangner N. , Moebius-Winkler S. , Leibundgut G. , Weilenmann D. , Woehrle J. , Richter S. , Schreiber M. , Mahfoud F. , Linke A. , Stephan F.-P. , Mueller C. , Rickenbacher P. , Coslovsky M. , Gilgen N. , Osswald S. , Kaiser C. , Scheller B. , and Investigators B.-S. , Drug-Coated Balloons for Small Coronary Artery Disease (BASKET-SMALL 2): An Open-Label Randomised Non-Inferiority Trial, Lancet. (2018) 392, no. 10150, 849–856, 10.1016/S0140-6736(18)31719-7, 2-s2.0-85053595566, 30170854. 30170854

[34] Tang Y. , Qiao S. , Su X. , Chen Y. , Jin Z. , Chen H. , Xu B. , Kong X. , Pang W. , Liu Y. , Yu Z. , Li X. , Li H. , Zhao Y. , Wang Y. , Li W. , Tian J. , Guan C. , Xu B. , Gao R. , and Investigators R. S. C. , Drug-Coated Balloon Versus Drug-Eluting Stent for Small-Vessel Disease, JACC. Cardiovascular Interventions. (2018) 11, no. 23, 2381–2392, 10.1016/j.jcin.2018.09.009, 2-s2.0-85057235371. 30522667

[35] Shin E.-S. , Lee J. M. , Her A.-Y. , Chung J.-H. , Lee K. E. , Garg S. , Nam C.-W. , Doh J.-H. , and Koo B.-K. , Prospective Randomized Trial of Paclitaxel-Coated Balloon Versus Bare-Metal Stent in High Bleeding Risk Patients With De Novo Coronary Artery Lesions, Coronary Artery Disease. (2019) 30, no. 6, 425–431, 10.1097/MCA.0000000000000755, 2-s2.0-85071064196. 31009399

[36] Vos N. S. , Fagel N. D. , Amoroso G. , Herrman J.-P. R. , Patterson M. S. , Piers L. H. , van der Schaaf R. J. , Slagboom T. , and Vink M. A. , Paclitaxel-Coated Balloon Angioplasty Versus Drug-Eluting Stent in Acute Myocardial Infarction the REVELATION Randomized Trial, Jacc-Cardiovascular Interventions. (2019) 12, no. 17, 1691–1699, 10.1016/j.jcin.2019.04.016, 2-s2.0-85070524485, 31126887. 31126887

[37] Jeger R. V. , Farah A. , Ohlow M.-A. , Mangner N. , Mobius-Winkler S. , Weilenmann D. , Woehrle J. , Stachel G. , Markovic S. , Leibundgut G. , Rickenbacher P. , Osswald S. , Cattaneo M. , Gilgen N. , Kaiser C. , Scheller B. , and Investigators B.-S. , Long-Term Efficacy and Safety of Drug-Coated Balloons Versus Drug-Eluting Stents for Small Coronary Artery Disease (BASKET-SMALL 2): 3-Year Follow-Up of a Randomised, Non-Inferiority Trial, Lancet. (2020) 396, no. 10261, 1504–1510, 10.1016/S0140-6736(20)32173-5, 33091360. 33091360

[38] Jing Q.-M. , Zhao X. , Han Y.-L. , Gao L.-L. , Zheng Y. , Li Z.-Q. , Yang P. , Cong H.-L. , Gao C.-Y. , Jiang T.-M. , Li H. , Li J.-X. , Wang D.-M. , Wang G. , Cong Z.-C. , and Zhang Z. , A Drug-Eluting Balloon for the trEatment of coronarY bifurcatiON Lesions in the Side Branch: A Prospective Multicenter ranDomized (BEYOND) Clinical Trial in China, Chinese Medical Journal. (2020) 133, no. 8, 899–908, 10.1097/CM9.0000000000000743. 32265425 PMC7176447

[39] Scheller B. , Ohlow M.-A. , Ewen S. , Kische S. , Rudolph T. K. , Clever Y. P. , Wagner A. , Richter S. , El-Garhy M. , Boehm M. , Degenhardt R. , Mahfoud F. , and Lauer B. , Bare Metal or Drug-Eluting Stent Versus Drug-Coated Balloon in Non-ST-Elevation Myocardial Infarction: The Randomised PEPCAD NSTEMI Trial, EuroIntervention. (2020) 15, no. 17, 10.4244/EIJ-D-19-00723. 31659986

[40] Tian J. , Tang Y.-d. , Qiao S. , Su X. , Chen Y. , Jin Z. , Chen H. , Xu B. , Kong X. , Pang W. , Liu Y. , Yu Z. , Li X. , Li H. , Zhao Y. , Wang Y. , Li W. , Guan C. , Gao R. , Xu B. , and Investigators R. S. C. , Two-Year Follow-Up of a Randomized Multicenter Study Comparing a Drug-Coated Balloon With a Drug-Eluting Stent in Native Small Coronary Vessels: The RESTORE Small Vessel Disease China Trial, Catheterization and Cardiovascular Interventions. (2020) 95, no. S1, 587–597, 10.1002/ccd.28705. 31943693

[41] Xu K. , Fu G. , Tong Q. , Liu B. , Han X. , Zhang J. , Ma G. , Yang Q. , Li H. , Zhou Y. , Jing Q. , Li Y. , and Han Y. , Biolimus-Coated Balloon in Small-Vessel Coronary Artery Disease: The BIO-RISE CHINA Study, JACC. Cardiovascular Interventions. (2022) 15, no. 12, 1219–1226, 10.1016/j.jcin.2022.03.024, 35738744. 35738744

[42] Yu X. , Wang X. , Ji F. , Zhang W. , Yang C. , Xu F. , and Wang F. , Correction to: A Non-Inferiority, Randomized Clinical Trial Comparing Paclitaxel-Coated Balloon Versus New-Generation Drug-Eluting Stents on Angiographic Outcomes for Coronary De Novo Lesions, Cardiovascular Drugs and Therapy. (2022) 36, no. 6, 1261–1262, 10.1007/s10557-021-07183-1, 33842993. 33842993 PMC9828854

[43] Cortese B. , Testa G. , Rivero F. , Erriques A. , and Alfonso F. , Long-Term Outcome of Drug-Coated Balloon vs Drug-Eluting Stent for Small Coronary Vessels PICCOLETO-II 3-Year Follow-Up, Jacc-Cardiovascular Interventions. (2023) 16, no. 9, 1054–1061, 10.1016/j.jcin.2023.02.011, 37164603. 37164603

[44] Qian J. , Wu Y. , Li C. , Yin J. , Fu G. , Ja W. , He Y. , Ma G. , Chen Y. , Xia Y. , Li L. , Ji F. , Zeng H. , Wei M. , Nie S. , Jin H. , He B. , Chen Y. , Liu F. , Wang H. , Sun Y. , Xu B. , Ge J. , and Study PCS , Drug-Coated Balloon for the Treatment of Small Vessel Disease: 9 Months of Angiographic Results and 12 Months of Clinical Outcomes of the PEPCAD China SVD Study, Catheterization and Cardiovascular Interventions. (2023) 101, no. 1, 33–43, 10.1002/ccd.30472. 36480798

[45] Nijhoff F. , Agostoni P. , Belkacemi A. , Nathoe H. M. , Voskuil M. , Samim M. , Doevendans P. A. , and Stella P. R. , Primary Percutaneous Coronary Intervention by Drug-Eluting Balloon Angioplasty: The Nonrandomized Fourth Arm of the DEB‐AMI (Drug‐Eluting Balloon in ST‐Segment Elevation Myocardial Infarction) Trial, Catheterization and Cardiovascular Interventions. (2015) 86, no. Supplement 1, S34–S44, 10.1002/ccd.26060, 2-s2.0-84940957987. 26119971

[46] Her A.-Y. , Ann S. H. , Singh G. B. , Kim Y. H. , Yoo S.-Y. , Garg S. , Koo B.-K. , and Shin E.-S. , Comparison of Paclitaxel-Coated Balloon Treatment and Plain Old Balloon Angioplasty for De Novo Coronary Lesions, Yonsei Medical Journal. (2016) 57, no. 2, 337–341, 10.3349/ymj.2016.57.2.337, 2-s2.0-84956948763, 26847284. 26847284 PMC4740524

[47] Shin E.-S. , Ann S. H. , Singh G. B. , Lim K. H. , Kleber F. X. , and Koo B.-K. , Fractional Flow Reserve-Guided Paclitaxel-Coated Balloon Treatment for De Novo Coronary Lesions, Catheterization and Cardiovascular Interventions. (2016) 88, no. 2, 193–200, 10.1002/ccd.26257, 2-s2.0-84982307675, 26423017. 26423017

[48] Sinaga D. A. , Hwa H. , Watson T. J. , Sim A. , Nyein T. T. , Jafary F. H. , Loh J. K. K. , Ooi Y. W. , Tan J. K. B. , and Ong P. J. L. , Drug-Coated Balloons: A Safe and Effective Alternative to Drug-Eluting Stents in Small Vessel Coronary Artery Disease, Journal of Interventional Cardiology. (2016) 29, no. 5, 454–460, 10.1111/joic.12333, 2-s2.0-84989338128, 27578540. 27578540

[49] Giannini F. , Latib A. , Ancona M. B. , Costopoulos C. , Ruparelia N. , Menozzi A. , Castriota F. , Micari A. , Cremonesi A. , De Felice F. , Marchese A. , Tespili M. , Presbitero P. , Sgueglia G. A. , Buffoli F. , Tamburino C. , Varbella F. , and Colombo A. , A Propensity Score Matched Comparative Study Between Paclitaxel-Coated Balloon and Everolimus-Eluting Stents for the Treatment of Small Coronary Vessels, Catheterization and Cardiovascular Interventions. (2017) 90, no. 3, 380–386, 10.1002/ccd.26929, 2-s2.0-85010204562. 28109036

[50] Her A.-Y. , Shin E.-S. , Lee J. M. , Garg S. , Doh J.-H. , Nam C.-W. , and Koo B.-K. , Paclitaxel-Coated Balloon Treatment for Functionally Nonsignificant Residual Coronary Lesions After Balloon Angioplasty, International Journal of Cardiovascular Imaging. (2018) 34, no. 9, 1339–1347, 10.1007/s10554-018-1351-z, 2-s2.0-85046032830. 29696453

[51] Sim H. W. , Ananthakrishna R. , Chan S. P. , Low A. F. , Lee C.-H. , Chan M. Y. , Tay E. L. , Loh P. H. , Chan K. H. , Tan H. C. , and Loh J. P. , Treatment of Very Small *De Novo* Coronary Artery Disease With 2.0 mm Drug-Coated Balloons Showed 1-Year Clinical Outcome Comparable With 2.0 mm Drug-Eluting Stents, Journal of Invasive Cardiology. (2018) 30, no. 7, 256–261, 29656281. 29656281

[52] Ueno K. , Morita N. , Kojima Y. , Takahashi H. , Kawasaki M. , Ito R. , Kondo H. , Minatoguchi S. , Yoshida T. , Hashimoto Y. , Tatsumi T. , and Kitamura T. , Safety and Long-Term Efficacy of Drug-Coated Balloon Angioplasty Following Rotational Atherectomy for Severely Calcified Coronary Lesions Compared With New Generation Drug-Eluting Stents, Journal of Interventional Cardiology. (2019) 2019, 9094178, 10.1155/2019/9094178, 2-s2.0-85064394165. 31772551 PMC6739772

[53] Silverio A. , Buccheri S. , Venetsanos D. , Alfredsson J. , Lagerqvist B. , Persson J. , Witt N. , James S. , and Sarno G. , Percutaneous Treatment and Outcomes of Small Coronary Vessels, JACC. Cardiovascular Interventions. (2020) 13, no. 7, 793–804, 10.1016/j.jcin.2019.10.062. 32061601

[54] Zhang D. , Wang L. , Liu Y. , Li K. , Xu L. , Li W. , Ni Z. , Xia K. , Zhang Z. , and Yang X. , Efficacy Comparison of Primary Percutaneous Coronary Intervention by Drug-Coated Balloon Angioplasty or Drug-Eluting Stenting in Acute Myocardial Infarction Patients With De Novo Coronary Lesions, Chinese Journal of Cardiology. (2020) 48, no. 7, 600–607, 10.3760/cma.j.cn112148-20200327-00254, 32842271. 32842271

[55] Iwasaki Y. , Koike J. , Ko T. , Funatsu A. , Kobayashi T. , Ikeda T. , and Nakamura S. , Comparison of Drug-Eluting Stents vs. Drug-Coated Balloon After Rotational Atherectomy for Severely Calcified Lesions of Nonsmall Vessels, Heart and Vessels. (2021) 36, no. 2, 189–199, 10.1007/s00380-020-01684-z, 32857188. 32857188

[56] Tan Q. , Wang Q. , Yang H. , Jing Z. , and Ming C. , Clinical Outcomes of Drug-Eluting Balloon for Treatment of Small Coronary Artery in Patients With Acute Myocardial Infarction, Internal and Emergency Medicine. (2021) 16, no. 4, 913–918, 10.1007/s11739-020-02530-w. 33386602

[57] Li C. , Ding X. , Wang L. , Li K. , Yang X. , Liu L. , and Xu L. , Feasibility and Safety of Drug-Coated Balloon-Only Angioplasty for De Novo Ostial Lesions of the Left Anterior Descending Artery: Two-Center Retrospective Study, Frontiers in Cardiovascular Medicine. (2022) 9, 874394, 10.3389/fcvm.2022.874394. 35548415 PMC9084228

[58] Li Y. , Mao Q. , Liu H. , Zhou D. , and Zhao J. , Effect of Paclitaxel-Coated Balloon Angioplasty on Side Branch Lesion and Cardiovascular Outcomes in Patients With De Novo True Coronary Bifurcation Lesions Undergoing Percutaneous Coronary Intervention, Cardiovascular Drugs and Therapy. (2022) 36, no. 5, 859–866, 10.1007/s10557-021-07225-8, 34241730. 34241730

[59] Liu H. , Tao H. , Han X. , Lu Y. , Xue X. , Feng R. , Lv F. , Liu Y. , Jin H. , Li L. , and Gu H. , Improved Outcomes of Combined Main Branch Stenting and Side Branch Drug-Coated Balloon Versus Two-Stent Strategy in Patients With Left Main Bifurcation Lesions, Journal of Interventional Cardiology. (2022) 2022, 8250057, 10.1155/2022/8250057. 35095348 PMC8767379

[60] Liu H. , Zhao Y. , Lu Y. , Zhou S. , Zhang Y. , Zhao J. , Yang H. , Xing J. , Feng R. , Xue X. , Tao H. , Song R. , and Gu H. , The Drug Coated Balloon-Only Strategy for Treatment of De Novo Left Main Coronary Artery Bifurcation Lesion: Stentless Strategy, Clinical and Applied Thrombosis/Hemostasis. (2022) 28, 10760296221118489, 10.1177/10760296221118489. 35945818 PMC9373168

[61] Mizutani Y. , Ishikawa T. , Nakamura H. , Yamada K. , Shimura M. , Kondo Y. , Ukaji T. , Aoki H. , Hisauchi I. , Itabashi Y. , Nakahara S. , and Taguchi I. , A Propensity Score-Matched Comparison of Midterm Outcomes Between Drug-Coated Balloons and Drug-Eluting Stents for Patients With Acute Coronary Syndrome, International Heart Journal. (2022) 63, no. 2, 217–225, 10.1536/ihj.21-576, 35185090. 35185090

[62] Pan L. , Lu W. , Han Z. , Pan S. , Wang X. , Shan Y. , Peng M. , Qin X. , Sun G. , Zhang P. , Dong J. , and Qiu C. , Drug-Coated Balloon in the Treatment of Coronary Left Main True Bifurcation Lesion: A Patient-Level Propensity-Matched Analysis, Frontiers in Cardiovascular Medicine. (2022) 9, 1028007, 10.3389/fcvm.2022.1028007, 36407423. 36407423 PMC9669294

[63] Tsai C.-H. , Yeh C.-F. , Meng S.-W. , Hung C.-S. , Lin M.-S. , Huang C.-C. , Chen C.-K. , Huang K.-P. , Chen Y.-H. , and Kao H.-L. , Comparison Between Drug-Coated Balloons and Drug-Eluting Stents in Very Small Coronary Artery Interventions, Scientific Reports. (2022) 12, no. 1, 10679, 10.1038/s41598-022-14047-7, 35739138. 35739138 PMC9226175

[64] Dong H. , Shan Y. , Gong S. , Li R. , Li Y. , Lu X. , and Sun G. , Clinical Research of Drug-Coated Balloon After Rotational Atherectomy for Severe Coronary Artery Calcification, BMC Cardiovascular Disorders. (2023) 23, no. 1, 10.1186/s12872-023-03071-8, 36681814. PMC9867860 36681814

[65] Funayama N. , Muratsubaki S. , Ito R. , Tobisawa T. , and Konishi T. , Drug-Coated Balloons Versus Drug-Eluting Stents for Coronary De Novo Lesions in Dialysis Patients, Heart and Vessels. (2023) 38, no. 3, 300–308, 10.1007/s00380-022-02169-x, 36045267. 36045267 PMC9898424

[66] Gunawardena T. D. , Corballis N. , Merinopoulos I. , Wickramarachchi U. , Reinhold J. , Maart C. , Sreekumar S. , Sawh C. , Wistow T. , Sarev T. , Ryding A. , Gilbert T. J. , Clark A. , Vassiliou V. S. , and Eccleshall S. , Drug-Coated Balloon vs. Drug-Eluting Stents for De Novo Unprotected Left Main Stem Disease: The SPARTAN-LMS Study, Journal of Cardiovascular Development and Disease. (2023) 10, no. 2, 10.3390/jcdd10020084. PMC9963161 36826580

[67] Her A.-Y. , Kim B. , Ahn S. H. , Park Y. , Cho J. R. , Jeong Y.-H. , and Shin E.-S. , Long-Term Clinical Outcomes of Drug-Coated Balloon Treatment for De Novo Coronary Lesions, Yonsei Medical Journal. (2023) 64, no. 6, 359–365, 10.3349/ymj.2022.0633, 37226562. 37226562 PMC10232998

[68] Merinopoulos I. , Gunawardena T. , Corballis N. , Bhalraam U. , Gilbert T. , Maart C. , Richardson P. , Ryding A. , Sarev T. , Sawh C. , Sulfi S. , Wickramarachchi U. , Wistow T. , Mohamed M. O. , Mamas M. A. , Vassiliou V. S. , and Eccleshall S. C. , Paclitaxel Drug-Coated Balloon-Only Angioplasty for De Novo Coronary Artery Disease in Elective Clinical Practice, Clinical Research in Cardiology. (2023) 112, no. 9, 1186–1193, 10.1007/s00392-022-02106-y, 36104455. 36104455 PMC10449668

[69] Merinopoulos I. , Gunawardena T. , Corballis N. , Bhalraam U. , Reinhold J. , Wickramarachchi U. , Maart C. , Gilbert T. , Richardson P. , Sulfi S. , Sarev T. , Sawh C. , Wistow T. , Ryding A. , Mohamed M. O. , Perperoglou A. , Mamas M. A. , Vassiliou V. S. , and Eccleshall S. C. , Assessment of Paclitaxel Drug-Coated Balloon Only Angioplasty in STEMI, JACC. Cardiovascular Interventions. (2023) 16, no. 7, 771–779, 10.1016/j.jcin.2023.01.380. 37045498

[70] Mitsui K. , Lee T. , Miyazaki R. , Hara N. , Nagamine S. , Nakamura T. , Terui M. , Okata S. , Nagase M. , Nitta G. , Watanabe K. , Kaneko M. , Nagata Y. , Nozato T. , and Ashikaga T. , Drug-Coated Balloon Strategy Following Orbital Atherectomy for Calcified Coronary Artery Compared With Drug-Eluting Stent: One-Year Outcomes and Optical Coherence Tomography Assessment, Catheterization and Cardiovascular Interventions. (2023) 102, no. 1, 11–17, 10.1002/ccd.30689, 37210618. 37210618

[71] Nakamura H. , Ishikawa T. , Mizutani Y. , Yamada K. , Ukaji T. , Kondo Y. , Shimura M. , Aoki H. , Hisauchi I. , Itabashi Y. , Nakahara S. , Kobayashi S. , and Taguchi I. , Clinical and Angiographic Outcomes of Elective Paclitaxel-Coated Balloon Angioplasty in Comparison With Drug-Eluting Stents for De Novo Coronary Lesions in Large Vessels, International Heart Journal. (2023) 64, no. 2, 145–153, 10.1536/ihj.22-498, 37005310. 37005310

[72] Yamada K. , Ishikawa T. , Nakamura H. , Mizutani Y. , Ukaji T. , Shimura M. , Kondo Y. , Aoki H. , Hisauchi I. , Itabashi Y. , Nakahara S. , Kobayashi S. , and Taguchi I. , Midterm Safety and Efficacy of Elective Drug-Coated Balloon Angioplasty in Comparison to Drug-Eluting Stents for Unrestrictive De Novo Coronary Lesions: A Single Center Retrospective Study, Journal of Cardiology. (2023) 81, no. 6, 537–543, 10.1016/j.jjcc.2022.11.014, 36481299. 36481299

[73] Cai X. , Hong X. , Wang Y. , Li Y. , and Xu G. , Drug-Coated Balloon in the Treatment of Coronary Artery De-Novo Large Lesions Angiography, Cellular and Molecular Biology. (2024) 70, no. 4, 196–201, 10.14715/cmb/2024.70.4.31. 38678606

[74] Goto J. , Niizeki T. , Iwayama T. , Sasaki T. , and Watanabe M. , One-Year Outcome of Drug-Coated Balloon vs. Drug-Eluting Stent in Patients Undergoing Initial Percutaneous Coronary Intervention (PCI) for De Novo Lesion, Cureus. (2024) 16, no. 3, e56346, 10.7759/cureus.56346. 38633944 PMC11021378

[75] Her A. Y. , Kim B. , Kim S. , Kim Y. H. , Scheller B. , and Shin E. S. , Comparison of Angiographic Change in Side-Branch Ostium After Drug-Coated Balloon vs. Drug-Eluting Stent vs. Medication for the Treatment of De Novo Coronary Bifurcation Lesions, European Journal of Medical Research. (2024) 29, no. 1, 10.1186/s40001-024-01877-6. PMC11089776 38735968

[76] Jeger R. V. , Eccleshall S. , Ahmad W. A. W. , Ge J. , Poerner T. C. , Shin E.-S. , Alfonso F. , Latib A. , Ong P. J. , Rissanen T. T. , Saucedo J. , Scheller B. , Kleber F. X. , and Int D. C. B. C. G. , Drug-Coated Balloons for Coronary Artery Disease, Jacc-Cardiovascular Interventions. (2020) 13, no. 12, 1391–1402, 10.1016/j.jcin.2020.02.043. 32473887

[77] Zhao H. , Miao R. , Lin F. , Zhao G. , and Presbitero P. , Drug-Coated Balloon in Primary Percutaneous Coronary Intervention, Journal of Interventional Cardiology. (2023) 2023, 10.1155/2023/5210808, 5210808. 37404481 PMC10317576

[78] Bosco E. , Hsueh L. , McConeghy K. W. , Gravenstein S. , and Saade E. , Major Adverse Cardiovascular Event Definitions Used in Observational Analysis of Administrative Databases: A Systematic Review, BMC Medical Research Methodology. (2021) 21, no. 1, 10.1186/s12874-021-01440-5, 34742250. PMC8571870 34742250

[79] Garcia-Garcia H. M. , McFadden E. P. , Farb A. , Mehran R. , Stone G. W. , Spertus J. , Onuma Y. , Morel M.-a. , van Es G.-A. , Zuckerman B. , Fearon W. F. , Taggart D. , Kappetein A.-P. , Krucoff M. W. , Vranckx P. , Windecker S. , Cutlip D. , Serruys P. W. , and Academic Research Consortium , Standardized End Point Definitions for Coronary Intervention Trials: The Academic Research Consortium-2 Consensus Document, Circulation. (2018) 137, no. 24, 2635–2650, 10.1161/CIRCULATIONAHA.117.029289, 2-s2.0-85053903425, 29891620. 29891620

[80] Zhao D. , Epidemiological Features of Cardiovascular Disease in Asia, JACC: Asia. (2021) 1, no. 1, 1–13, 10.1016/j.jacasi.2021.04.007, 36338365. 36338365 PMC9627928

[81] Bartnik M. , Ryden L. , Ferrari R. , Malmberg K. , Pyorala K. , Simoons M. , Standl E. , Soler-Soler J. , Ohrvik J. , and Euro Heart Survey I , The Prevalence of Abnormal Glucose Regulation in Patients With Coronary Artery Disease Across Europe. The Euro Heart Survey on Diabetes and the Heart, European Heart Journal. (2004) 25, no. 21, 1880–1890, 10.1016/j.ehj.2004.07.027, 2-s2.0-7544224325, 15522466. 15522466

[82] Zhou M. , Liu J. , Hao Y. , Liu J. , Huo Y. , Smith S. C.Jr., Ge J. , Ma C. , Han Y. , Fonarow G. C. , Taubert K. A. , Morgan L. , Yang N. , Xing Y. , Zhao D. , and Investigators C.-A. , Prevalence and In-Hospital Outcomes of Diabetes Among Patients With Acute Coronary Syndrome in China: Findings From the Improving Care for Cardiovascular Disease in China-Acute Coronary Syndrome Project, Cardiovascular Diabetology. (2018) 17, no. 1, 10.1186/s12933-018-0793-x, 2-s2.0-85057251168. PMC6258152 30482187

[83] Low Wang C. C. , Hess C. N. , Hiatt W. R. , and Goldfine A. B. , Clinical Update: Cardiovascular Disease in Diabetes Mellitus, Circulation. (2016) 133, no. 24, 2459–2502, 10.1161/CIRCULATIONAHA.116.022194, 2-s2.0-84974829707. 27297342 PMC4910510

[84] Kolandaivelu K. , Swaminathan R. , Gibson W. J. , Kolachalama V. B. , Nguyen-Ehrenreich K. L. , Giddings V. L. , Coleman L. , Wong G. K. , and Edelman E. R. , Stent Thrombogenicity Early in High-Risk Interventional Settings Is Driven by Stent Design and Deployment and Protected by Polymer-Drug Coatings, Circulation. (2011) 123, no. 13, 1400–1409, 10.1161/CIRCULATIONAHA.110.003210, 2-s2.0-79954633893, 21422389. 21422389 PMC3131199

[85] Bangalore S. , Toklu B. , Patel N. , Feit F. , and Stone G. W. , Newer-Generation Ultrathin Strut Drug-Eluting Stents Versus Older Second-Generation Thicker Strut Drug-Eluting Stents for Coronary Artery Disease, Circulation. (2018) 138, no. 20, 2216–2226, 10.1161/CIRCULATIONAHA.118.034456, 2-s2.0-85058037527. 29945934

[86] Sedhom R. , Hamed M. , Elbadawi A. , Mohsen A. , Swamy P. , Athar A. , Bharadwaj A. S. , Prasad V. , Elgendy I. Y. , and Alfonso F. , Outcomes With Limus- vs Paclitaxel-Coated Balloons for Percutaneous Coronary Intervention: Meta-Analysis of Randomized Controlled Trials, JACC. Cardiovascular Interventions. (2024) 17, no. 13, 1533–1543, 10.1016/j.jcin.2024.04.042, 38986653. 38986653

[87] Clever Y. P. , Peters D. , Calisse J. , Bettink S. , Berg M. C. , Sperling C. , Stoever M. , Cremers B. , Kelsch B. , Bohm M. , Speck U. , and Scheller B. , Novel Sirolimus-Coated Balloon Catheter: In Vivo Evaluation in a Porcine Coronary Model, Circulation. Cardiovascular Interventions. (2016) 9, no. 4, e003543, 10.1161/CIRCINTERVENTIONS.115.003543, 2-s2.0-84964553508, 27069105. 27069105

[88] Spaulding C. , Krackhardt F. , Bogaerts K. , Urban P. , Meis S. , Morice M. C. , and Eccleshall S. , Comparing a Strategy of Sirolimus-Eluting Balloon Treatment to Drug-Eluting Stent Implantation in De Novo Coronary Lesions in All-Comers: Design and Rationale of the SELUTION DeNovo Trial, American Heart Journal. (2023) 258, 77–84, 10.1016/j.ahj.2023.01.007, 36642225. 36642225

[89] Kawai K. , Rahman M. T. , Nowicki R. , Kolodgie F. D. , Sakamoto A. , Kawakami R. , Konishi T. , Virmani R. , Labhasetwar V. , and Finn A. V. , Efficacy and Safety of Dual Paclitaxel and Sirolimus Nanoparticle-Coated Balloon, JACC: Basic to Translational Science. (2024) 9, no. 6, 774–789, 10.1016/j.jacbts.2024.02.002, 39070273. 39070273 PMC11282887

