# Supplementary material for: Drug‐Coated Balloons Versus Other Percutaneous Coronary Intervention Strategies in De Novo Coronary Artery Disease: A Systematic Review, Meta‐Analysis With Trial Sequential Analysis
Source: Cardiovasc Ther. 2026 Jan 1;2026:5568664. doi: 10.1155/cdr/5568664 (PMC12757438; doi:10.1155/cdr/5568664)

**SUPPLEMENTARY MATERIAL**

**Drug-Coated Balloons versus Other Percutaneous Coronary Intervention Strategies in De Novo Coronary Artery Disease: A Systematic Review, Meta-Analysis with Trial Sequential Analysis**

**Online-only Data Supplement**

| **Table S1** | Full search strategy and search terms | 3-4 |
| --- | --- | --- |
| **Table S2** | Patient and disease characteristics in eligible studies | 5-8 |
| **Table S3** | Target lesion characteristics | 9-11 |
| **Figure S1** | Bias assessment of the included RCTs according to the Cochrane Collaboration | 12 |
| **Figure S2** | Forest plot of risk ratios for secondary clinical outcomes | 13 |
| **Figure S3** | Forest plot of mean differences (MD) for secondary imaging outcomes | 14 |
| **Figure S4** | Forest plot of secondary clinical outcomes in RCTs and cohort studies | 14 |
| **Figure S5** | Forest plot of secondary clinical outcomes in RCTs and cohort studies | 15 |
| **Figure S6** | Forest plot of secondary imaging outcomes in RCTs and cohort studies | 16 |
| **Figure S7** | Forest plot of primary outcomes stratified by vessel diameter | 17 |
| **Figure S8** | Forest plot of primary outcomes stratified by DAPT | 18 |
| **Figure S9** | Forest plot of primary outcomes according to the routine use of intravascular imaging techniques | 19 |
| **Figure S10** | Forest plot of primary outcomes in patients with ACS | 20 |
| **Figure S11** | Forest plot of MACE in patients with diabetes mellitus | 20 |
| **Figure S12** | Forest plot of primary outcomes in bifurcation lesions | 20 |
| **Figure S13** | Funnel plot for publication bias of studies included in the meta-analysis | 21 |
| **Figure S14** | Sensitivity analysis | 22 |
| **Figure S15** | Forest plot for the sensitivity analysis using standardized MACE stratified by comparator category (DCB vs uncoated device and DCB vs DES). | 23 |
| **Figure S16** | Forest plot for the sensitivity analysis using standardized MACE stratified by study design (RCT vs cohort). | 23 |
| **Figure S17** | Forest plot for the sensitivity analysis using standardized MACE stratified by region (Asia vs Europe). | 24 |

**Supplementary Table 1. Full search strategy and search terms**

| **Database** | **Search strategy** | **Results** |
| --- | --- | --- |
| Pubmed | (((drug coated balloon OR drug eluting balloon OR paclitaxel eluting balloon OR paclitaxel coated balloon OR sirolimus coated balloon OR sirolimus eluting balloon OR DCB OR PCB OR SCB))) [All Fields] AND ((*de novo* vessel OR *de novo* lesion OR *de novo* narrowing OR *de novo* coronary lesion OR *de novo* coronary artery lesion OR *de novo* coronary stenosis OR *de novo* coronary vessel OR native vessel OR native lesion OR native narrowing OR native coronary lesion OR native coronary artery lesion OR native coronary stenosis OR native coronary vessel OR *de novo* coronary artery disease OR *de novo* native coronary artery lesion OR *de novo* native coronary stenosis OR native coronary artery *de novo* lesion)) [All Fields] | 1,068 |
| Embase | 1.'drug coated balloon':ta,ab,kw OR 'drug eluting balloon':ta,ab,kw OR 'paclitaxel eluting balloon':ta,ab,kw OR 'paclitaxel coated balloon':ta,ab,kw OR 'sirolimus coated balloon':ta,ab,kw OR 'sirolimus eluting balloon':ta,ab,kw OR 'dcb':ta,ab,kw OR 'pcb':ta,ab,kw OR 'scb':ta,ab,kw OR 'limus-coated balloon':ta,ab,kw OR 'biolimus-coated balloon'  2.'de novo vessel':ta,ab,kw OR 'de novo lesion':ta,ab,kw OR 'de novo narrowing':ta,ab,kw OR 'native vessel':ta,ab,kw OR 'native lesion':ta,ab,kw OR 'native narrowing':ta,ab,kw OR 'onary lesion':ta,ab,kw OR 'onary vessel':ta,ab,kw OR 'de novo c':ta,ab,kw OR 'onary artery disease':ta,ab,kw OR 'onary artery lesion':ta,ab,kw OR 'de novo native c':ta,ab,kw OR 'onary stenosis':ta,ab,kw OR 'native c':ta,ab,kw OR 'onary artery de novo lesion'  3. 1 AND 2 | 85 |
| wos | 1. TS=(drug coated balloon or drug eluting balloon or paclitaxel eluting balloon or paclitaxel coated balloon or sirolimus coated balloon or sirolimus eluting balloon or DCB or PCB or SCB or Limus-coated balloon or Biolimus-Coated Balloon) 2. TS=(de novo vessel OR de novo lesion OR de novo narrowing OR de novo coronary lesion OR de novo coronary artery lesion OR de novo coronary stenosis OR de novo coronary vessel OR native vessel OR native lesion OR native narrowing OR native coronary lesion OR native coronary artery lesion OR native coronary stenosis OR native coronary vessel OR de novo coronary artery disease OR de novo native coronary artery lesion OR de novo native coronary stenosis OR native coronary artery de novo lesion) 3. 1 AND 2 | 1503 |
| Cochrane Library | 1. (drug coated balloon OR drug eluting balloon OR paclitaxel eluting balloon OR paclitaxel coated balloon OR sirolimus coated balloon OR sirolimus eluting balloon OR DCB OR PCB OR SCB OR limus-coated balloon OR biolimus-coated balloon):ti,ab,kw 2. (de novo vessel OR de novo lesion OR de novo narrowing OR de novo coronary lesion OR de novo coronary artery lesion OR de novo coronary stenosis OR de novo coronary vessel OR native vessel OR native lesion OR native narrowing OR native coronary lesion OR native coronary artery lesion OR native coronary stenosis OR native coronary vessel OR de novo coronary artery disease OR de novo native coronary artery lesion OR de novo native coronary stenosis OR native coronary artery de novo lesion):ti,ab,kw 3. 1 AND 2 | 492 |

Search was conducted to identify articles up to November 14, 2024

**Supplementary Table 2. Patient and disease characteristics in eligible studies**

| First Author/Study | Year | Device | Age(y) | Male (%) | BMI | Clinical | | | | | | | | | | |
| --- | --- | --- | --- | --- | --- | --- | --- | --- | --- | --- | --- | --- | --- | --- | --- | --- |
|  |  |  |  |  |  | Hypertension (%) | DM(%) | Hyperlipemia (%) | Smoking (%) | Prior MI (%) | Prior PCI (%) | Prior CABG(%) | Family History(%) | CKD（%） | Stable angina（%） | ACS(%) |
| PICCOLETO | 2011 | DCB | 68±9 | 78.6 | - | 75.0 | 37.9 | 60.7 | - | 17.9 | 10.7 | 10.7 | - | - | 46.4 | 53.6 |
|  |  | DES | 67±10 | 75.9 | - | 70.8 | 46.4 | 54.2 | - | 20.7 | 13.8 | 13.8 | - | - | 44.8 | 55.2 |
| BELLO | 2012 | DCB | 64.8±8.5 | 80 | - | 80.0 | 43.3 | 78.9 | 16.7 | 51.1 | 57.8 | 10.0 | 26.7 | - | - | 24.4 |
|  |  | DES | 66.4±9.0 | 77.2 | - | 81.5 | 38.0 | 79.3 | 10.9 | 35.9 | 42.4 | 13.0 | 25.0 | - | - | 21.7 |
| BABILON | 2014 | DCB | 63.9±11.3 | 63.5 | - | 61.5 | 26.9 | 69.2 | 48.1 | 28.8 | 23.1 | - | - | - | - | 44.2 |
|  |  | DES | 65.6±11.1 | 66.1 | - | 62.5 | 35.7 | 58.9 | 51.8 | 21.4 | 12.5 | - | - | - | - | 42.9 |
| PEPCAD-BIF | 2016 | DCB | 66 ± 12 | 75 | - | - | 34.4 | - | 46.9 | 12.5 | - | - | - | - | 71.9 | 28.1 |
|  |  | POBA | 69 ± 10 | 71.9 | - | - | 37.5 | - | 65.6 | 25.0 | - | - | - | - | 81.2 | 18.8 |
| Naoki Nishiyama | 2016 | DCB | 67.30 ± 11.12 | 67 | - | 76.7 | 40.0 | 80.0 | 56.7 | - | - | - | 13.3 | 6.7 | - | - |
|  |  | POBA | 70.63 ± 8.97 | 80 | - | 90.0 | 43.3 | 76.7 | 63.3 | - | - | - | 10.0 | 13.3 | - | - |
| Atsushi Funatsu | 2017 | DCB | 68 ± 10 | 78 | - | 84.0 | 48.0 | 74.0 | - | - | - | - | - | - | - | - |
|  |  | POBA | 69 ± 11 | 68 | - | 73.0 | 32.0 | 88.0 | - | - | - | - | - | - | - | - |
| David Gobic | 2017 | DCB | 57.2 ± 13.1 | 70.7 | 29.1 ± 4.1 | 31.7 | 4.9 | 9.5 | 43.9 | - | - | - | 39.0 | 4.9 | - | - |
|  |  | DES | 54.3 ± 10.6 | 73 | 28.2 ± 3.7 | 35.1 | 10.8 | 18.9 | 56.8 | - | - | - | 37.8 | 0.0 | - | - |
| BASKET-SMALL 2 | 2018 | DCB | 67.2 ±10.3 | 77 | 28.4 ±4.5 | 85.0 | 32.0 | 69.0 | 60.0 | 42.0 | 62.0 | 10.0 | 43.0 | 14.0 | 70.0 | 30.0 |
|  |  | DES | 68.4 ±10.3 | 70 | 28.2 ±4.6 | 89.0 | 35.0 | 70.0 | 53.0 | 35.0 | 64.0 | 9.0 | 38.0 | 16.0 | 73.0 | 27.0 |
| RESTORE SVD China | 2018 | DCB | 60.1 ± 10.5 | 66.4 | 25.6 ± 3.2 | 67.2 | 39.7 | 52.6 | 29.3 | 22.4 | 38.8 | 0.0 | 24.1 | - | 31.0 | 69.0 |
|  |  | DES | 60.5 ± 10.8 | 77.2 | 25.4 ± 3.1 | 75.4 | 42.1 | 48.2 | 31.6 | 24.6 | 33.3 | 0.9 | 11.4 | - | 28.9 | 71.1 |
| DEBUT | 2019 | DCB | 77.6 ±8.4 | 62 | - | 87.0 | 26.0 | 78.0 | 34.0 | 23.0 | - | - | - | - | 54.0 | 46.0 |
|  |  | BMS | 76.2 ±8.5 | 64 | - | 91.0 | 49.0 | 84.0 | 33.0 | 19.0 | - | - | - | - | 54.0 | 46.0 |
| Eun-Seok Shin | 2019 | DCB | 57.5 ± 9.2 | 70 | 24.4 ± 2.5 | 40.0 | 35.0 | 50.0 | 40.0 | 5.0 | 10.0 | - | 5.0 | - | 70.0 | 30.0 |
|  |  | BMS | 61.6 ± 9.5 | 75 | 23.5 ± 3.8 | 45.0 | 25.0 | 50.0 | 25.0 | 0.0 | 0.0 | - | 10.0 | - | 60.0 | 40.0 |
| REVELATION | 2019 | DCB | 57.4±9.2 | 87 | 26.7±3.5 | 30.0 | 13.0 | 17.0 | 60.0 | - | 3.0 | 0.0 | 32.0 | - | - | - |
|  |  | DES | 57.3±8.3 | 87 | 27.4±4.4 | 32.0 | 7.0 | 13.0 | 50.0 | - | 0.0 | 0.0 | 42.0 | - | - | - |
| PICCOLETO II | 2020 | DCB | 64 (48-80) | 70.3 | - | 65.2 | 38.0 | 61.0 | 19.5 | 38.0 | 50.0 | 3.3 | - | 3.3 | 54.2 | 45.8 |
|  |  | DES | 66 (50-82) | 76.9 | - | 67.2 | 35.4 | 55.0 | 16.7 | 30.0 | 53.0 | 3.5 | - | 10.6 | 55.7 | 44.3 |
| BEYOND | 2020 | DCB | 59.9 ± 10.1 | 79.7 | - | 61.1 | 30.1 | 21.2 | 55.8 | - | - | - | - | - | 4.4 | 92.0 |
|  |  | POBA | 61.8 ± 9.4 | 65.1 | - | 59.6 | 34.9 | 25.7 | 51.4 | - | - | - | - | - | 3.7 | 91.7 |
| PEPCAD NSTEMI | 2020 | DCB | 66.0±11.4 | 66.3 | 28.7±5.2 | 78.7 | 26.9 | 50.0 | 57.7 | 19.2 | - | - | 26.0 | - | - | - |
|  |  | BMS/DES | 67.0±13.1 | 67.9 | 28.4±4.9 | 87.7 | 35.8 | 45.3 | 64.2 | 16.0 | - | - | 29.2 | - | - | - |
| BIO-RISE CHINA | 2022 | DCB | 61.3±8.8 | 72.4 | 25.8±2.9 | 68.6 | 34.3 | 17.1 | 44.8 | 20.0 | - | - | 10.5 | - | 40.0 | 60.0 |
|  |  | POBA | 61.6±8.1 | 66.3 | 26.2±3.8 | 67.3 | 34.7 | 17.8 | 42.6 | 26.7 | - | - | 6.3 | - | 38.6 | 61.4 |
| Yu | 2022 | DCB | 62.6±8.8 | 73.8 | 26.0±3.0 | 59.5 | 19.0 | 61.9 | 54.8 | - | 13.1 | 1.2 | - | - | 9.5 | 90.5 |
|  |  | DES | 64.0±10.5 | 70.9 | 25.4±3.0 | 68.4 | 29.1 | 49.4 | 53.2 | - | 17.7 | 3.8 | - | - | 12.7 | 87.3 |
| PEPCAD China SVD | 2023 | DCB | 63.8 ± 8.9 | 74 | 25.2 ± 3.2 | 70.7 | 32.0 | 24.3 | 26.1 | 17.1 | 43.6 | 0.6 | - | - | 26.0 | 64.1 |
|  |  | POBA | 63.3 ± 8.4 | 71.3 | 25.2 ± 3.0 | 70.1 | 39.1 | 19.5 | 24.1 | 10.3 | 42.5 | 0.0 | - | - | 27.6 | 67.8 |
| REC-CAGEFREE I | 2024 | DCB | 62 (55–69) | 67.9 | 24.6 (22.5–26.8) | 58.4 | 24.9 | 79.7 | 42.7 | 7.1 | 12.1 | - | - | 8.4 | 44.4 | 55.6 |
|  |  | DES | 62 (54–69) | 70.7 | 24.4 (22.5–27.0) | 61.8 | 29.7 | 79.7 | 46.5 | 9.2 | 12.5 | - | - | 9.5 | 45.0 | 55.0 |
| F. Nijhoff | 2015 | DCB | 57.9.10.0 | 65 | - | 35.0 | 12.5 | 17.5 | 52.5 | 7.5 | 2.5 | 0.0 | - | - | - | - |
|  |  | BMS | 59.9.10.9 | 82.4 | - | 35.3 | 11.8 | 21.6 | 56.9 | 0.0 | 2.0 | 0.0 | - | - | - | - |
|  |  | DES | 55.9.9.7 | 83.7 | - | 30.6 | 4.1 | 32.7 | 57.1 | 2.0 | 2.0 | 0.0 | - | - | - | - |
| Ae-Young Her | 2016 | DCB | 61.8±9.1 | 71.4 | - | 65.3 | 44.9 | 67.3 | 36.7 | - | - | - | 6.1 | - | 51.0 | 49.0 |
|  |  | POBA | 65.7±8.7 | 60.9 | - | 56.5 | 13.0 | 43.5 | 13.0 | - | - | - | 17.4 | - | 34.8 | 65.2 |
| Eun-Seok Shin | 2016 | DCB | 60.6±7.4 | 77.3 | 23.8±2.6 | 54.5 | 29.5 | 43.2 | 45.5 | 6.8 | 15.9 | - | 15.9 | - | 70.5 | 29.5 |
|  |  | DES | 58.7±10.2 | 72.7 | 24.6±3.1 | 36.4 | 22.7 | 36.4 | 22.7 | 0.0 | 4.5 | - | 27.3 | - | 54.5 | 31.8 |
| DASDO ANTONIUS SINAGA | 2016 | DCB | 61.0±11.8 | 76.7 | - | 72.7 | 51.2 | 69.8 | 30.2 | 28.5 | - | - | - | - | 22.1 | 77.9 |
|  |  | DES | 61.2±10.7 | 71.8 | - | 69.3 | 49.1 | 72.4 | 44.8 | 27.6 | - | - | - | - | 34.4 | 62.2 |
| Francesco Giannini | 2017 | DCB | 65±9 | 80 | - | 80.0 | 43.3 | 78.9 | 43.3 | 51.1 | 57.8 | 10.0 | 26.7 | - | 75.6 | 24.4 |
|  |  | DES | 66±10 | 78 | - | 81.3 | 40.0 | 78.0 | 33.0 | 58.2 | 57.1 | 16.5 | 35.2 | - | 81.1 | 18.9 |
| Ae‑Young Her | 2018 | DCB | 58.6 ± 8.6 | 71.8 | 24.3 ± 2.5 | 51.3 | 32.1 | 57.7 | 30.8 | 7.7 | - | - | 12.8 | - | 48.7 | 51.3 |
|  |  | DES/BMS | 59.0 ± 8.7 | 80.8 | 24.4 ± 3.0 | 42.5 | 21.9 | 42.5 | 27.4 | 5.5 | - | - | 13.7 | - | 39.7 | 60.3 |
| H. W. Sim | 2018 | DCB | 58.1 ± 11.9 | 80.5 | - | 79.3 | 63.2 | 80.5 | 48.3 | - | 36.8 | 9.2 | - | 5.7 | - | - |
|  |  | DES | 61.3 ± 11.2 | 77 | - | 68.0 | 54.0 | 76.5 | 29.5 | - | 24.0 | 7.5 | - | 15.5 | - | - |
| Katsumi Ueno | 2019 | DCB | 71±9 | 70.4 | - | 70.4 | 61.1 | 70.4 | 18.5 | 31.5 | - | 13.0 | - | 35.2 | - | - |
|  |  | DES | 71±9 | 65.2 | - | 76.8 | 56.5 | 62.3 | 29.0 | 29.0 | - | 8.7 | - | 40.6 | - | - |
| A. Silverio | 2020 | DCB | 68.0 (61.0–76.0) | 70.1 | 26.8 (24.5–29.7) | 68.5 | 26.9 | 58.9 | 55.2 | 31.6 | 34.1 | 11.5 | - | - | 32.3 | 67.7 |
|  |  | DES | 69.0 (61.0–76.0) | 66.4 | 26.6 (24.3–29.7) | 64.4 | 23.0 | 50.6 | 57.1 | 24.8 | 24.0 | 9.3 | - | - | 23.5 | 76.5 |
| D. Zhang | 2020 | DCB | 66.4±12.3 | 67.8 | - | 42.2 | 13.9 | - | 55.6 | - | - | - | 12.2 | - | - | 100.0 |
|  |  | DES | 63.1±18.2 | 76 | - | 35.0 | 20.0 | - | 54.0 | - | - | - | 19.0 | - | - | 100.0 |
| Yoshihiro Iwasaki | 2021 | DCB | 76 ± 7.2 | 64 | - | 79.0 | 54.0 | 50.0 | 60.0 | - | - | 3.0 | - | 9.0 | 98.0 | 2.0 |
|  |  | DES | 74 ± 8.4 | 67 | - | 68.0 | 47.0 | 59.0 | 64.0 | - | - | 5.0 | - | 19.0 | 98.0 | 2.0 |
| Qiang Tan | 2021 | DCB | 64.96 ± 8.82 | 61 | - | 37.5 | 32.1 | - | 51.8 | 8.9 | - | - | 30.4 | - | - | 100.0 |
|  |  | DES | 62.39 ± 9.91 | 66 | - | 34.7 | 26.9 | - | 43.5 | 7.5 | - | - | 32.9 | - | - | 100.0 |
| Chuang Li | 2022 | DCB | 62 (54, 68) | 76 | - | 47.0 | 24.0 | 31.0 | 45.0 | 2.0 | 16.0 | - | - | - | - | - |
|  |  | DES | 61(55, 75) | 71 | - | 43.0 | 24.0 | 35.0 | 61.0 | 6.0 | 12.0 | - | - | - | - | - |
| Youmei Li | 2022 | DCB | 64 ± 11 | 80.4 | - | 51.0 | 32.4 | 23.5 | 56.9 | - | - | - | - | - | - | - |
|  |  | POBA | 63 ± 10 | 81.2 | - | 55.6 | 27.4 | 23.9 | 53.9 | - | - | - | - | - | - | - |
| Hengdao Liu | 2022a | DCB | 57.86±11.39 | 80.56 | - | 50.0 | 27.8 | 11.1 | 30.6 | 19.4 | 25.0 | - | - | - | 22.2 | 77.8 |
|  |  | DES | 62.20 ± 10.41 | 77.55 | - | 51.0 | 24.5 | 2.0 | 22.5 | 12.2 | 22.5 | - | - | - | 26.5 | 73.5 |
| Hengdao Liu | 2022b | DCB | 61.76 ± 11.17 | 76 | - | 52.0 | 20.0 | 4.0 | 50.0 | 6.0 | 20.0 | - | - | - | 34.0 | 66.0 |
|  |  | DES | 64.74 ± 9.20 | 72 | - | 36.0 | 34.0 | 10.0 | 40.0 | 10.0 | 14.0 | - | - | - | 24.0 | 76.0 |
| Yukiko Mizutani | 2022 | DCB | 67.5 ± 12.3 | 81.3 | - | 81.3 | 36.3 | 95.6 | - | 3.3 | - | - | - | - | - | 100.0 |
|  |  | DES | 67.9 ± 12.3 | 90.2 | - | 72.5 | 36.3 | 90.1 | - | 5.5 | - | - | - | - | - | 100.0 |
| Liang Pan | 2022 | DCB | 63.75 ± 8.13 | 72.86 | - | 53.8 | 38.7 | 27.1 | 36.2 | 5.5 | 15.6 | 1.5 | 21.6 | 3.5 | 37.7 | 62.3 |
|  |  | DES | 63.75 ± 8.13 | 75.13 | - | 51.5 | 41.5 | 29.9 | 34.7 | 6.5 | 18.6 | 1.8 | 22.6 | 6.0 | 40.7 | 59.3 |
| Cheng‑Hsuan Tsai | 2022 | DCB | 65.1 ± 10.6 | 83 | 26.8 ± 3.9 | 93.6 | 48.9 | 85.0 | 44.7 | 10.6 | - | 6.4 | - | 12.8 | 87.2 | 12.8 |
|  |  | DES | 65.1 ± 10.0 | 81.4 | 25.4 ± 3.9 | 83.0 | 54.2 | 71.2 | 35.6 | 11.9 | - | 5.1 | - | 10.2 | 81.4 | 18.6 |
| Haozhe Dong | 2023 | DCB | 66.68 ± 9.13 | 52.63 | - | 66.7 | 42.1 | 12.3 | 24.6 | 19.3 | 21.1 | 0.0 | - | 7.0 | - | - |
|  |  | DES | 65.64 ± 7.99 | 59 | - | 65.1 | 36.8 | 23.4 | 29.9 | 16.1 | 11.1 | 1.2 | - | 11.1 | - | - |
| Naohiro Funayama | 2023 | DCB | 68.0 ± 10.3 | 80.3 | - | 83.3 | 68.2 | 48.4 | 21.2 | 9.1 | 51.5 | 18.2 | - | 100.0 | 95.5 | 4.5 |
|  |  | DES | 69.6 ± 9.7 | 84.8 | - | 89.4 | 72.7 | 47.0 | 33.3 | 6.1 | 37.9 | 10.6 | - | 100.0 | 83.3 | 16.7 |
| SPARTAN LMS | 2023 | DCB | 73.8 ± 11.7 | 85.4 | - | 56.1 | 31.7 | 26.8 | 64.9 | 22.0 | 19.5 | 2.4 | 9.8 | - | 24.4 | 68.3 |
|  |  | DES | 69.8 ± 11.1 | 76.6 | - | 52.3 | 22.4 | 24.3 | 56.3 | 24.3 | 29.0 | 1.9 | 9.3 | - | 40.2 | 53.2 |
| Ae-Young Her | 2023 | DCB | 58.3±9.9 | 72.8 | 24.2±2.9 | 57.3 | 35.0 | - | 58.3 | 12.6 | 27.2 | - | - | 4.9 | 38.8 | 61.2 |
|  |  | DES | 58.2±10.4 | 69.9 | 24.1±2.8 | 49.5 | 35.0 | - | 54.4 | 11.7 | 26.2 | - | - | 2.9 | 50.5 | 49.5 |
| Ioannis Merinopoulos | 2023a | DCB | 69 (61–75) | 79 | - | 56.0 | 23.0 | 34.0 | 62.0 | 17.0 | 15.0 | 8.6 | 27.0 | - | - | - |
|  |  | DES | 69 (61–75) | 78 | - | 57.0 | 22.0 | 32.0 | 66.0 | 18.0 | 12.0 | 8.1 | 25.0 | - | - | - |
| Ioannis Merinopoulos | 2023b | DCB | 66±13 | 74 | - | 40.0 | 14.0 | 17.0 | 58.0 | 6.6 | 5.5 | 1.1 | 9.7 | - | 0.0 | 100.0 |
|  |  | DES | 66±11 | 73 | - | 35.0 | 12.0 | 15.0 | 67.0 | 5.1 | 4.2 | 0.9 | 10.0 | - | 0.0 | 100.0 |
| Kentaro Mitsui | 2023 | DCB | 75.0 (70.0, 79.0) | 86 | - | 95.3 | 48.8 | 74.4 | 30.2 | 30.2 | 53.5 | 2.3 | - | - | 90.7 | 9.3 |
|  |  | DES | 74.5 (68.0, 80.0) | 80.4 | - | 81.5 | 52.2 | 70.7 | 34.8 | 27.2 | 48.9 | 5.4 | - | - | 92.4 | 7.6 |
| Hidehiko Nakamura | 2023 | DCB | 67.1 ± 11.4 | 84.9 | 24.8 ± 3.9 | 91.8 | 49.3 | 76.7 | 50.7 | 24.7 | 39.7 | 5.5 | - | 15.1 | - | - |
|  |  | DES | 69.0 ± 10.4 | 79.3 | 23.5 ± 3.6 | 81.7 | 49.4 | 87.7 | 49.4 | 18.5 | 43.2 | 7.4 | - | 16.0 | - | - |
| Kota Yamada | 2023 | DCB | 69.1 ± 10.9 | 79.1 | - | - | 58.8 | - | 50.3 | 33.3 | 43.5 | 2.8 | - | 16.4 | 62.7 | 37.3 |
|  |  | DES | 67.9 ± 10.9 | 82.5 | - | - | 50.3 | - | 48.6 | 31.1 | 40.7 | 4.5 | - | 23.7 | 63.3 | 36.7 |
| X. Cai | 2024 | DCB | 63.33±14.14 | 66.7 | 25.87±3.83 | 84.4 | 42.4 | - | 57.6 | - | - | - | - | - | 9.1 | 90.9 |
|  |  | DES | 61.95±13.18 | 70.9 | 24.56±3.21 | 72.7 | 34.5 | - | 49.1 | - | - | - | - | - | 20.0 | 80.0 |
| Jun Goto | 2024 | DCB | 72.5 ± 11.3 | 76 | 23.1 ± 5.1 | 80.0 | 38.7 | 72.0 | 14.7 | - | - | - | - | - | 69.3 | 30.7 |
|  |  | DES | 72.6 ± 11.4 | 74.4 | 24.0 ± 3.3 | 81.2 | 39.1 | 67.1 | 20.3 | - | - | - | - | - | 45.4 | 54.6 |
| Ae‑Young Her | 2024 | DCB | 61.6 ± 9.0 | 72.7 | - | 68.2 | 36.4 | 61.4 | 20.5 | - | - | - | 18.2 | - | 34.1 | 65.9 |
|  |  | DES | 63.0 ± 9.7 | 71.1 | - | 57.9 | 36.8 | 31.6 | 27.0 | - | - | - | 10.5 | - | 21.0 | 79.0 |

Abbreviations: BMI, Body Mass Index; DM, Diabetes Mellitus; MI, Myocardial Infarction; PCI, Percutaneous Coronary Intervention; CABG, Coronary Artery Bypass Grafting; CKD, Chronic Kidney Disease; ACS, Acute Coronary Syndrome; DCB, Drug-Coated Balloon; DES, Drug-Eluting Stent; BMS, Bare Metal Stent; POBA, Plain Old Balloon Angioplasty.

**Supplementary Table 3. Target lesion characteristics.**

| First Author/Study | Year | Device | Target vessel | | | | | | | | |
| --- | --- | --- | --- | --- | --- | --- | --- | --- | --- | --- | --- |
|  |  |  | LM(%) | LAD(%) | RCA(%) | LCx(%) | Bifurcation(%) | Multivessel disease(%) | Calcified(%) | CTO(%) | RVD(mm) |
| PICCOLETO | 2011 | DCB | - | - | - | - | 21.4 | - | - | - | 2.45±0.28 |
|  |  | DES | - | - | - | - | 24.1 | - | - | - | 2.36±0.25 |
| BELLO | 2012 | DCB | - | 27.6 | 35.1 | 37.2 | - | 62.2 | - | - | 2.41± 0.34 |
|  |  | DES | - | 20.6 | 30.9 | 48.5 | - | 60.9 | - | - | 2.41± 0.40 |
| BABILON | 2014 | DCB | - | 61.5 | 9.6 | 28.8 | 100.0 | - | - | - | 2.29±0.46 |
|  |  | DES | - | 66.1 | 10.7 | 23.2 | 100.0 | - | - | - | 2.35±0.26 |
| PEPCAD-BIF | 2016 | DCB | - | 43.8 | 6.3 | 50.0 | - | - | - | - | 2.38 ± 0.38 |
|  |  | POBA | - | 53.1 | 6.3 | 40.6 | - | - | - | - | 2.41 ± 0.39 |
| Naoki Nishiyama | 2016 | DCB | - | 44.4 | 40.7 | 14.8 | - | - | - | - | 2.88 ± 0.57 |
|  |  | POBA | - | 51.5 | 30.3 | 18.2 | - | - | - | - | 2.72 ± 0.64 |
| Atsushi Funatsu | 2017 | DCB | - | 33.0 | 27.0 | 40.0 | - | - | - | - | 2.04 ± 0.39 |
|  |  | POBA | - | 35.0 | 14.0 | 51.0 | - | - | - | - | 1.99 ± 0.28 |
| David Gobic | 2017 | DCB | - | - | - | - | - | - | - | - | 2.61 ± 0.49 |
|  |  | DES | - | - | - | - | - | - | - | - | 3.04 ± 0.46 |
| BASKET-SMALL 2 | 2018 | DCB | - | 34.0 | 20.0 | 47.0 | 6.0 | 82.0 | - | - | - |
|  |  | DES | - | 31.0 | 20.0 | 49.0 | 8.0 | 76.0 | - | - | - |
| RESTORE SVD China | 2018 | DCB | - | 20.7 | 25.0 | 54.3 | - | 41.4 | - | - | 2.11±0.27 |
|  |  | DES | - | 22.0 | 31.6 | 46.5 | - | 39.5 | - | - | 2.21±0.29 |
| DEBUT | 2019 | DCB | - | 45.0 | 27.0 | 26.0 | 17.0 | - | 10.0 | - | - |
|  |  | BMS | - | 46.0 | 27.0 | 25.0 | 13.0 | - | 11.0 | - | - |
| Eun-Seok Shin | 2019 | DCB | - | 30.0 | 45.0 | 25.0 | - | - | - | - | 3.0 ± 0.4 |
|  |  | BMS | - | 40.0 | 50.0 | 10.0 | - | - | - | - | 3.2 ± 0.3 |
| REVELATION | 2019 | DCB | - | 32.0 | 48.0 | 20.0 | - | 26.7 | - | - | 3.28 ± 0.52 |
|  |  | DES | - | 40.0 | 47.0 | 13.0 | - | 29.7 | - | - | 3.20 ±0.48 |
| PICCOLETO II | 2020 | DCB | - | 40.0 | 22.8 | 37.2 | 12.7 | 72.8 | - | - | 2.23 ± 0.4 |
|  |  | DES | - | 39.0 | 30.2 | 31.0 | 12.3 | 76.0 | - | - | 2.18 ± 0.4 |
| BEYOND | 2020 | DCB | - | 75.2 | 8.0 | 13.3 | 100.0 | - | - | - | 2.15 ± 0.33 |
|  |  | POBA | - | 78.0 | 3.7 | 15.6 | 100.0 | - | - | - | 2.10 ± 0.29 |
| PEPCAD NSTEMI | 2020 | DCB | - | - | - | - | - | - | - | - | - |
|  |  | BMS/DES | - | - | - | - | - | - | - | - | - |
| BIO-RISE CHINA | 2022 | DCB | - | - | - | - | - | 2.9 | - | - | 1.99±0.31 |
|  |  | POBA | - | - | - | - | - | 4.9 | - | - | 2.05±0.31 |
| Yu | 2022 | DCB | - | 57.1 | 14.3 | 28.6 | - | 83.3 | 13.1 | - | 2.77 (2.50-3.25) |
|  |  | DES | - | 44.3 | 35.4 | 20.3 | - | 84.8 | 15.2 | - | 3.01 (2.65-3.39) |
| PEPCAD China SVD | 2023 | DCB | - | - | - | - | - | 3.9 | - | - | 2.03 ± 0.33 |
|  |  | POBA | - | - | - | - | - | 1.0 | - | - | 2.09 ± 0.36 |
| REC-CAGEFREE I | 2024 | DCB | - | 51.8 | 25.1 | 23.1 | 32.5 | - | - | - | - |
|  |  | DES | - | 49.6 | 25.4 | 25.0 | 31.1 | - | - | - | - |
| F. Nijhoff | 2015 | DCB | - | 45.0 | 32.5 | 22.5 | - | - | - | - | 2.83±0.51 |
|  |  | BMS | - | 41.2 | 43.1 | 15.7 | - | - | - | - | 2.84±0.58 |
|  |  | DES | - | 36.7 | 40.1 | 22.4 | - | - | - | - | 2.78±0.53 |
| Ae-Young Her | 2016 | DCB | - | 61.2 | 14.3 | 24.5 | - | - | - | - | 2.3±0.5 |
|  |  | POBA | - | 21.7 | 43.5 | 34.8 | - | - | - | - | 2.1±0.5 |
| Eun-Seok Shin | 2016 | DCB | - | - | - | - | - | - | - | - | 2.55±0.41 |
|  |  | DES | - | - | - | - | - | - | - | - | 2.70±0.42 |
| DASDO ANTONIUS SINAGA | 2016 | DCB | - | 41.3 | 26.2 | 32.0 | - | 12.2 | - | 1.2 | 2.22±0.30 |
|  |  | DES | - | 41.1 | 23.3 | 34.4 | - | 19.0 | - | 1.2 | 2.44±0.19 |
| Francesco Giannini | 2017 | DCB | - | 27.6 | 35.1 | 37.2 | - | 61.1 | - | - | - |
|  |  | DES | - | 33.0 | 30.8 | 30.9 | - | 71.4 | - | - | - |
| Ae‑Young Her | 2018 | DCB | - | 44.9 | 23.0 | 32.0 | - | - | - | - | 2.4 ± 0.5 |
|  |  | DES/BMS | - | 57.5 | 28.8 | 13.7 | - | - | - | - | 2.6 ± 0.5 |
| H. W. Sim | 2018 | DCB | - | - | - | - | - | 72.4 | - | - | 1.88 ± 0.38 |
|  |  | DES | - | - | - | - | - | 78.0 | - | - | 1.95 ± 0.21 |
| Katsumi Ueno | 2019 | DCB | 2.9 | 55.9 | 13.2 | 27.9 | 67.6 | - | - | 4.4 | 2.28±0.58 |
|  |  | DES | 5.1 | 48.0 | 35.7 | 11.2 | 47.9 | - | - | 11.2 | 2.49±0.55 |
| A. Silverio | 2020 | DCB | 0.4 | 49.2 | 15.2 | 35.2 | 19.8 | 39.0 | - | 3.5 | - |
|  |  | DES | 0.5 | 44.8 | 22.3 | 32.4 | 12.0 | 51.0 | - | 4.2 | - |
| D. Zhang | 2020 | DCB | 1.1 | 57.8 | 15.6 | 12.2 | 53.3 | - | 11.1 | - | - |
|  |  | DES | 4.5 | 61.5 | 16.0 | 14.0 | 41.0 | - | 12.5 | - | - |
| Yoshihiro Iwasaki | 2021 | DCB | 2.0 | 59.0 | 23.0 | 16.0 | - | 38.0 | 100.0 | - | 2.97 ± 0.45 |
|  |  | DES | 3.0 | 60.0 | 15.0 | 22.0 | - | 50.0 | 100.0 | - | 3.03 ± 0.36 |
| Qiang Tan | 2021 | DCB | - | 53.6 | 19.6 | 26.8 | 37.5 | 67.9 | - | - | 2.64 ± 0.17 |
|  |  | DES | - | 55.2 | 28.8 | 16.0 | 28.8 | 75.0 | - | - | 2.65 ± 0.14 |
| Chuang Li | 2022 | DCB | - | - | - | - | 92.0 | 41.0 | 2.0 | - | 3.44 (3.04, 3.63) |
|  |  | DES | - | - | - | - | 82.0 | 61.0 | 10.0 | - | 3.52 (3.22, 3.63) |
| Youmei Li | 2022 | DCB | 58.8 | 36.3 | - | 4.9 | - | - | - | - | 2.52 ± 0.35 |
|  |  | POBA | 39.3 | 52.1 | - | 8.6 | - | - | - | - | 2.47 ± 0.38 |
| Hengdao Liu | 2022a | DCB | - | - | - | - | - | - | - | - | 3.62 （3.55,4.35） |
|  |  | DES | - | - | - | - | - | - | - | - | 3.81 （3.57,4.11） |
| Hengdao Liu | 2022b | DCB | - | - | - | - | - | 100.0 | - | - | 2.78 (2.60, 3.02) |
|  |  | DES | - | - | - | - | - | 100.0 | - | - | 2.98 (2.55, 3.37) |
| Yukiko Mizutani | 2022 | DCB | 1.1 | 53.8 | 25.3 | 19.8 | 22.0 | 45.1 | 4.4 | 1.1 | 2.44 ± 0.560 |
|  |  | DES | 2.2 | 50.5 | 19.8 | 27.5 | 18.7 | 42.9 | 2.2 | 0.0 | 2.83 ± 0.617 |
| Liang Pan | 2022 | DCB | - | - | - | - | - | 87.9 | 10.1 | - | 3.00 ± 0.49 |
|  |  | DES | - | - | - | - | - | 87.4 | 11.3 | - | 2.98 ± 0.32 |
| Cheng‑Hsuan Tsai | 2022 | DCB | - | 42.0 | 22.0 | 36.0 | - | 91.5 | - | 14.0 | - |
|  |  | DES | - | 44.3 | 13.1 | 42.6 | - | 94.9 | - | 19.7 | - |
| Haozhe Dong | 2023 | DCB | 1.7 | 72.4 | 13.8 | 12.1 | 3.5 | - | - | 10.3 | - |
|  |  | DES | 24.5 | 55.5 | 15.3 | 4.7 | 18.6 | - | - | 9.1 | - |
| Naohiro Funayama | 2023 | DCB | 1.5 | 30.3 | 30.3 | 33.3 | 36.4 | - | 48.5 | 1.5 | 2.56 ± 0.57 |
|  |  | DES | 4.5 | 47.0 | 27.3 | 21.2 | 24.2 | - | 53.0 | 4.5 | 2.53 ± 0.52 |
| SPARTAN LMS | 2023 | DCB | - | - | - | - | 73.2 | - | - | - | 3.84 ± 0.26 |
|  |  | DES | - | - | - | - | 48.6 | - | - | - | 4.33 ± 0.60 |
| Ae-Young Her | 2023 | DCB | 1.9 | 60.2 | 33.0 | 48.5 | - | 34.0 | - | 5.8 | - |
|  |  | DES | 0.0 | 50.5 | 25.2 | 25.2 | - | 32.0 | - | 7.8 | - |
| Ioannis Merinopoulos | 2023a | DCB | 2.8 | 57.0 | 20.0 | 19.0 | 12.0 | 9.4 | - | - | 3.00 (2.75–3.50) |
|  |  | DES | 3.9 | 54.0 | 25.0 | 14.0 | 8.1 | 12.0 | - | - | 3.50 (3.00–3.75) |
| Ioannis Merinopoulos | 2023b | DCB | 0.4 | 43.0 | 39.0 | 17.0 | 42.0 | 3.8 | 15.0 | - | 3.50 (3.00-3.50) |
|  |  | DES | 0.7 | 37.0 | 47.0 | 15.0 | 29.0 | 3.8 | 13.0 | - | 3.50 (3.00-4.00 |
| Kentaro Mitsui | 2023 | DCB | - | - | 37.2 | 11.6 | - | - | - | - | - |
|  |  | DES | - | - | 20.7 | 10.9 | - | - | - | - | - |
| Hidehiko Nakamura | 2023 | DCB | 1.4 | 34.2 | 39.7 | 23.3 | 15.1 | 49.3 | 6.8 | 0.0 | 3.23 ± 0.42 |
|  |  | DES | 6.2 | 37.0 | 44.4 | 12.3 | 14.8 | 38.3 | 30.9 | 4.9 | 3.59 ± 0.59 |
| Kota Yamada | 2023 | DCB | 4.5 | 33.9 | 31.1 | 35.0 | - | - | 11.9 | 2.3 | 2.39 ± 0.59 |
|  |  | DES | 2.3 | 36.2 | 30.5 | 32.2 | - | - | 8.5 | 4.0 | 2.74 ± 0.57 |
| X. Cai | 2024 | DCB | - | 39.4 | 27.3 | 33.3 | - | - | - | - | 3.8±0.74 |
|  |  | DES | - | 63.6 | 21.8 | 14.5 | - | - | - | - | 3.82±0.67 |
| Jun Goto | 2024 | DCB | 5.3 | 53.3 | 17.3 | 24.0 | 38.7 | 20.0 | 25.3 | - | - |
|  |  | DES | 3.8 | 43.1 | 34.7 | 18.3 | 35.3 | 39.7 | 31.7 | - | - |
| Ae‑Young Her | 2024 | DCB | 18.2 | 47.7 | 13.6 | 20.5 | 100.0 | - | - | - | - |
|  |  | DES | 26.3 | 52.6 | 5.3 | 15.8 | 100.0 | - | - | - | - |

Abbreviations: LM, Left Main Coronary Artery; LAD, Left Anterior Descending Artery; RCA, Right Coronary Artery; LCx, Left Circumflex Artery; Bifurcation, Bifurcation Lesion; Multivessel disease, Multivessel Coronary Artery Disease; Calcified, Calcified Lesion; CTO, Chronic Total Occlusion; RVD, Reference Vessel Diameter; DCB, Drug-Coated Balloon; DES, Drug-Eluting Stent; BMS, Bare Metal Stent; POBA, Plain Old Balloon Angioplasty.**Supplementary Figure 1. Bias assessment of the included RCTs according to the Cochrane Collaboration**

**
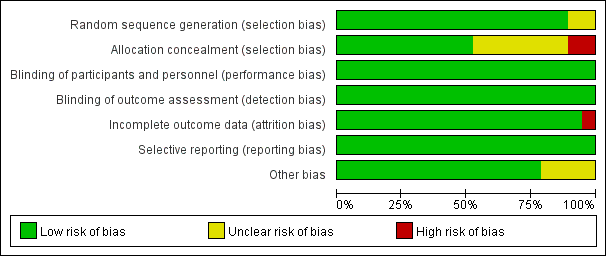

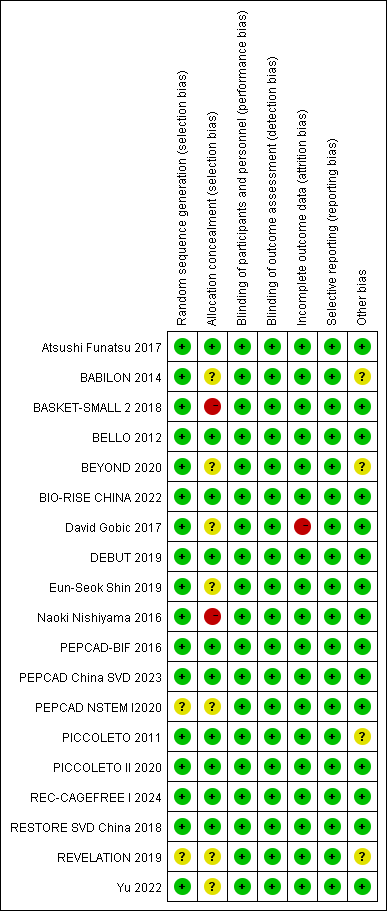
**

**Supplementary Figure 2. Forest plot of risk ratios for secondary clinical outcomes.**

**
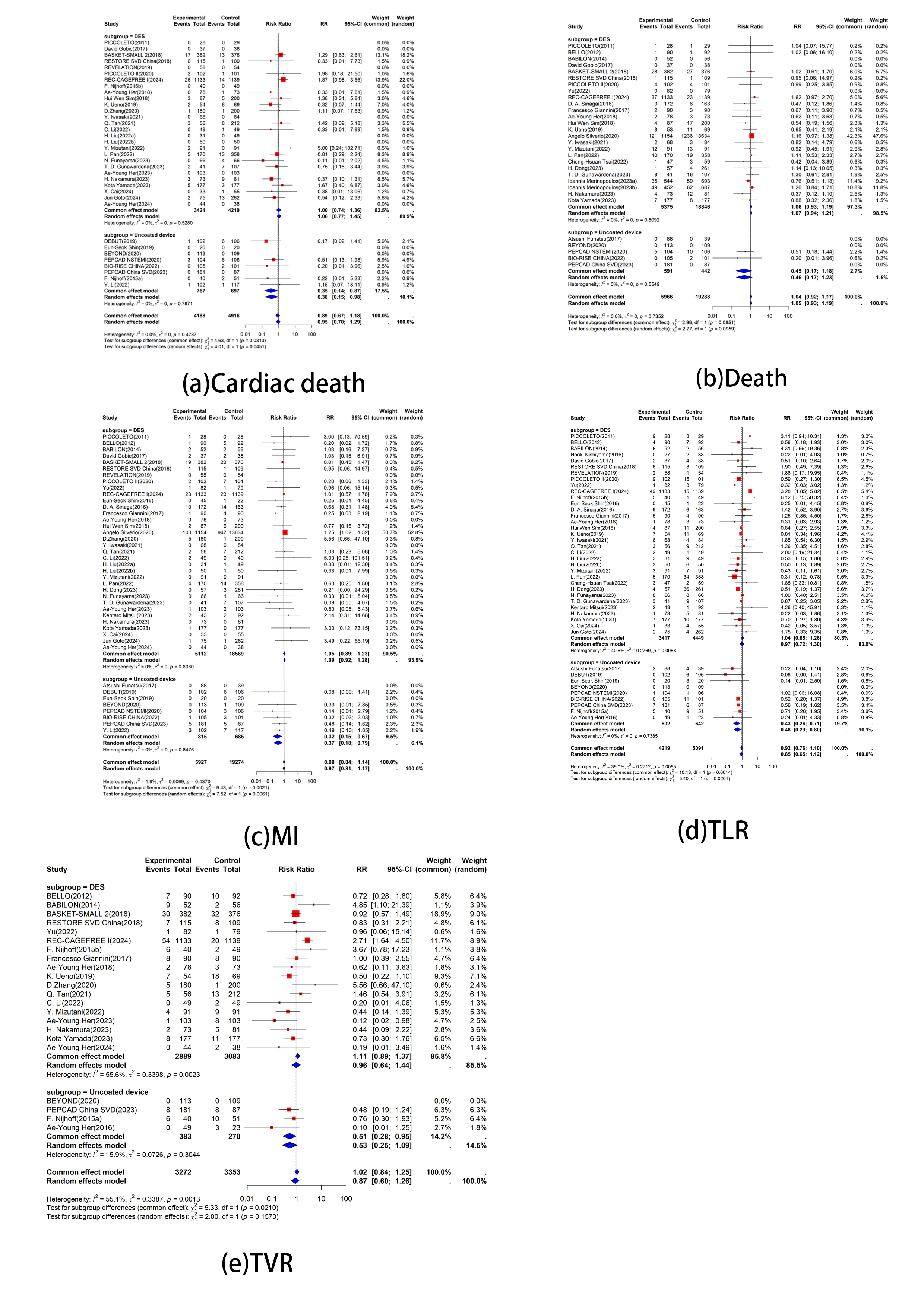
**

Abbreviations: MI, myocardial infarction; TLR, target lesion revascularization; TVR, target vessel revascularization; CI, confidence interval; DCB, drug-coated balloon; DES, drug-eluting stent; RR, risk ratio.

**Supplementary Figure 3. Forest plot of mean differences (MD) for secondary imaging outcomes**

**
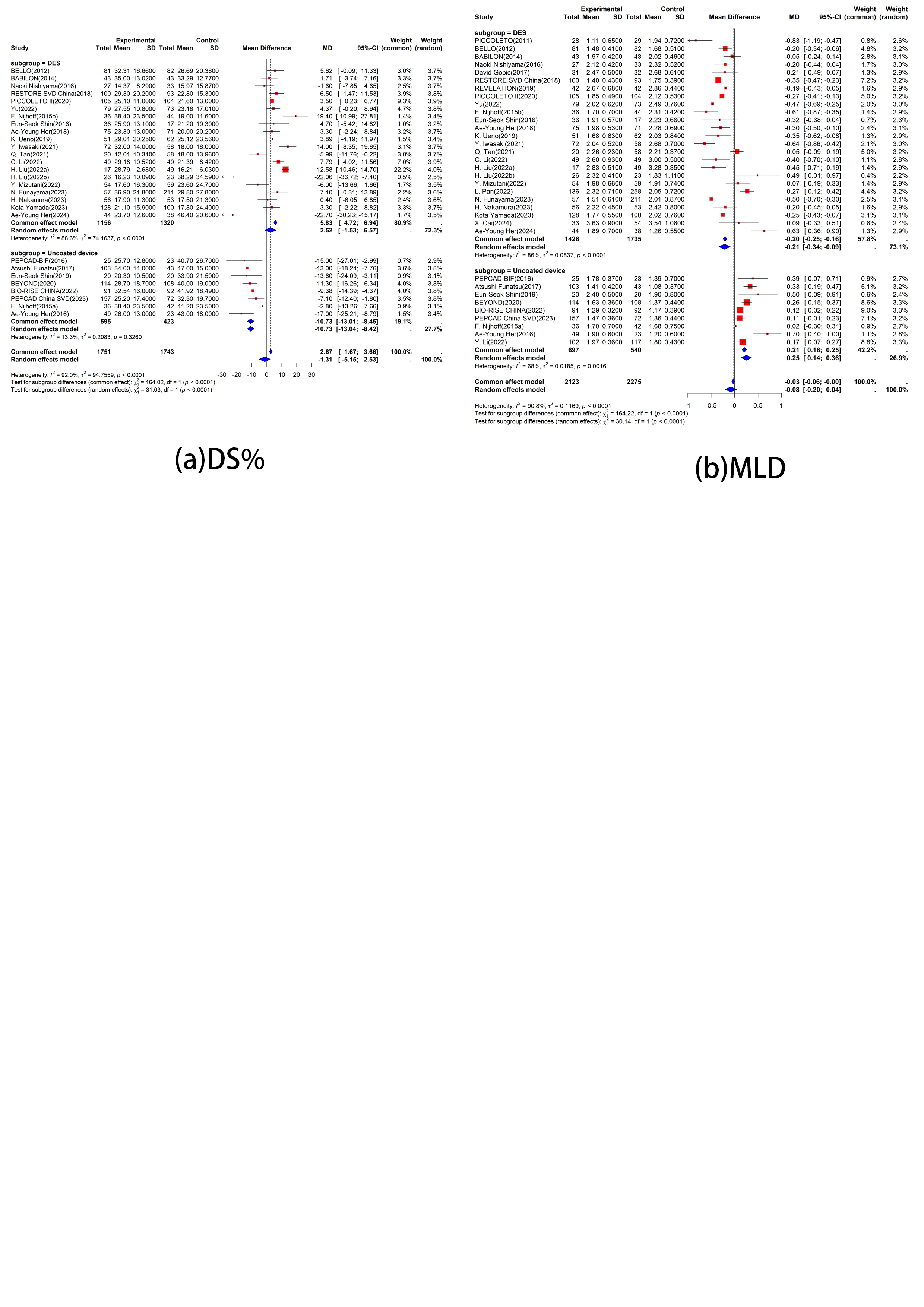
**

Abbreviations: DS%, percentage diameter stenosis; MLD, minimum lumen diameter; CI, confidence interval; MD, mean differences; DCB, drug-coated balloon; DES, drug-eluting stent.

**Supplementary Figure 4. Forest plot of secondary clinical outcomes in RCTs and cohort studies.**

**
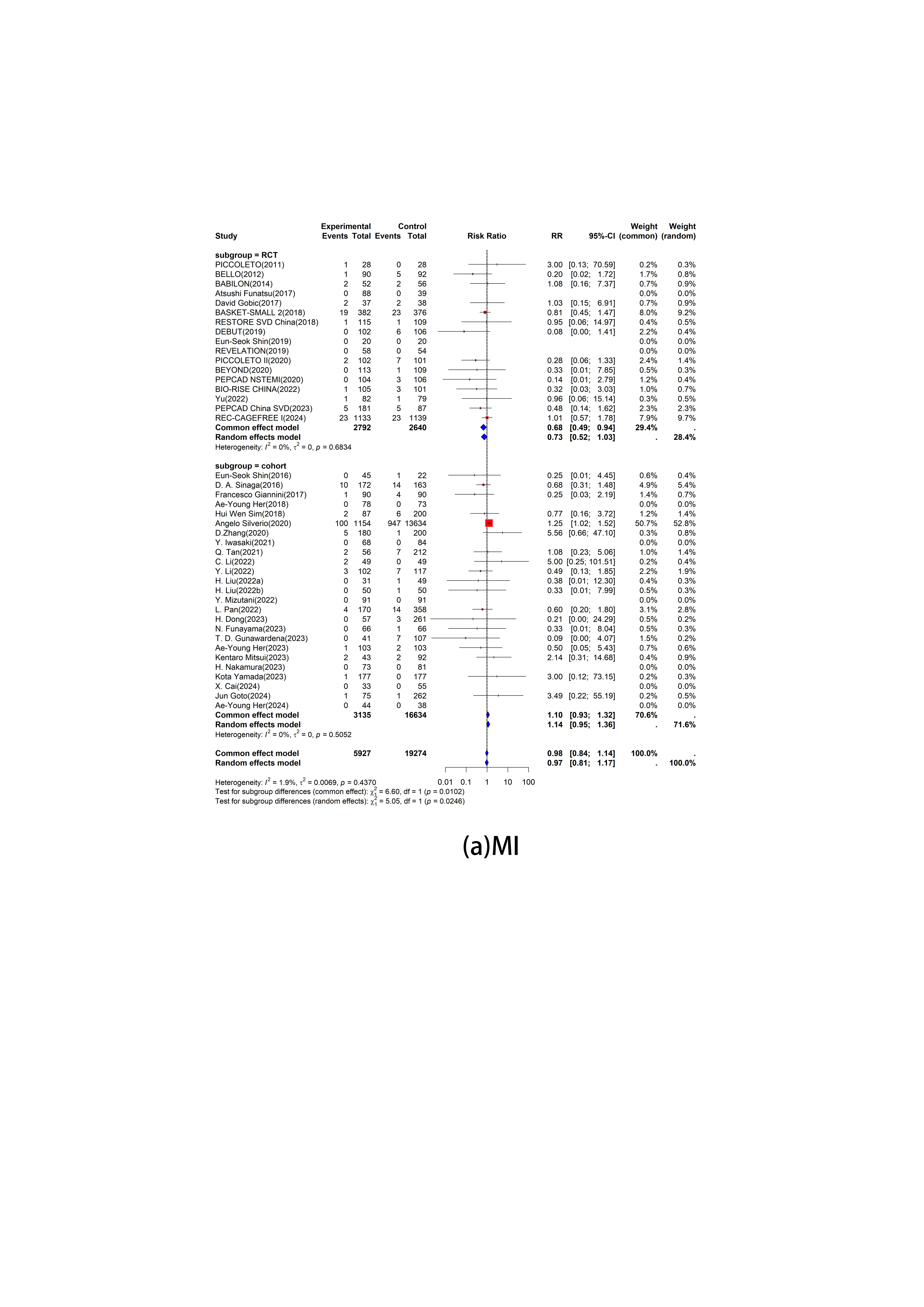
**

Abbreviations: MI, myocardial infarction; RCT, randomized controlled trial; RR, risk ratio; CI, confidence interval.

**Supplementary Figure 5. Forest plot of secondary clinical outcomes in RCTs and cohort studies.**

**
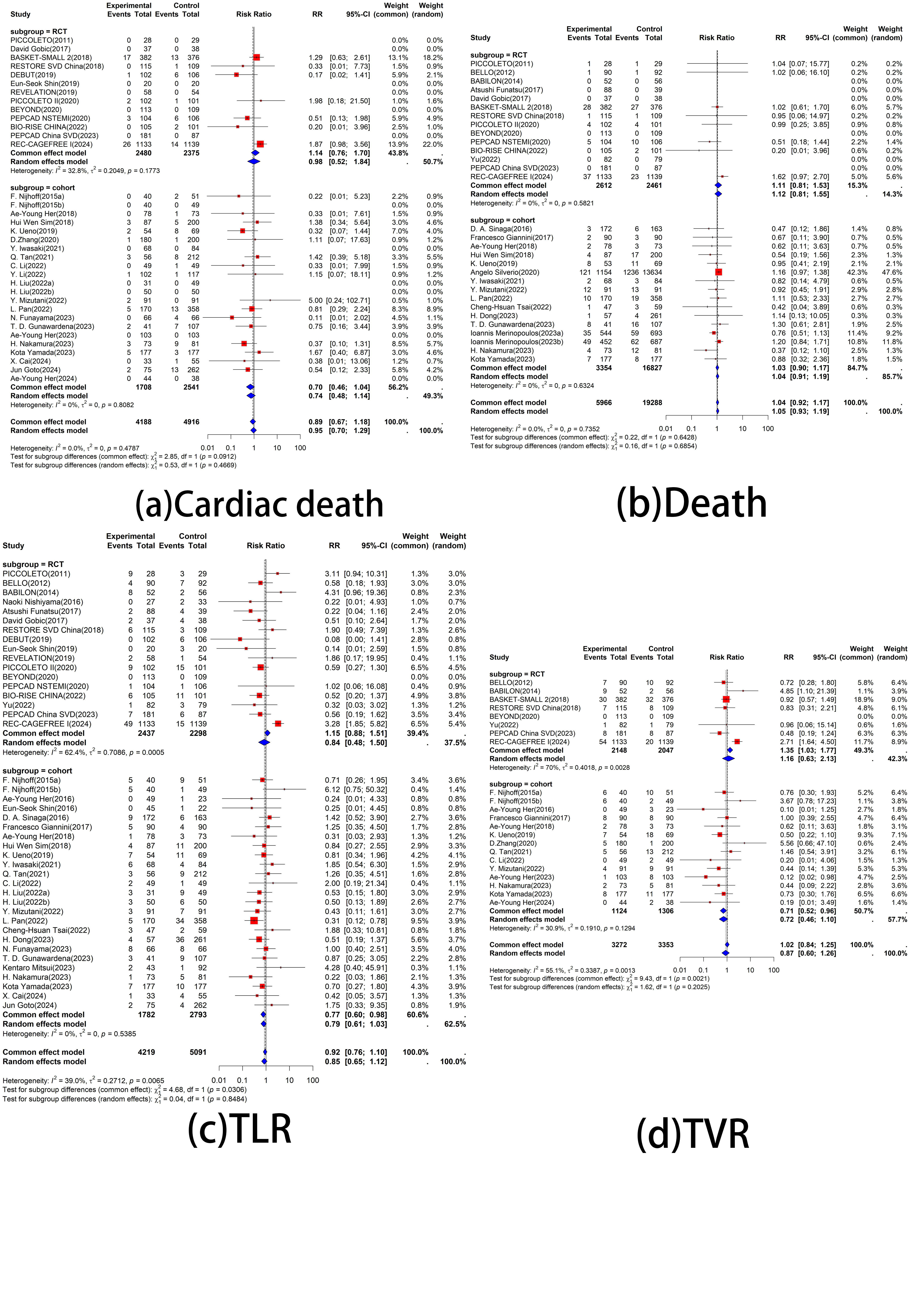
**

Abbreviations: TLR, target lesion revascularization; TVR, target vessel revascularization; RCT, randomized controlled trial; RR, risk ratio; CI, confidence interval.

**Supplementary Figure 6. Forest plot of secondary imaging outcomes in RCTs and cohort studies.**


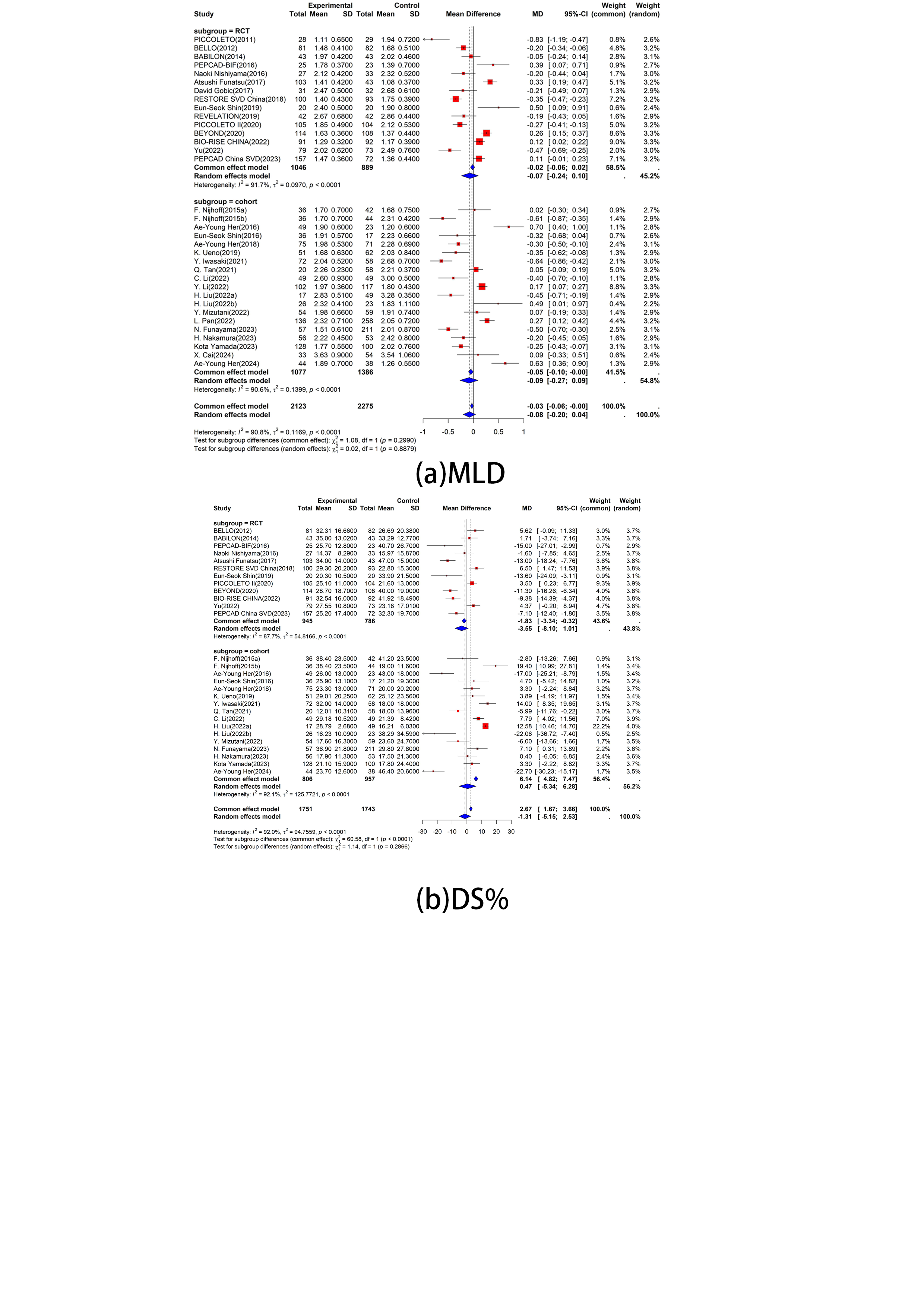


Abbreviations: RCT, randomized controlled trial; DS%, percentage diameter stenosis; MLD, minimum lumen diameter; MD, mean differences; CI, confidence interval.

**Supplementary Figure 7.Forest plot of primary outcomes stratified by vessel diameter**


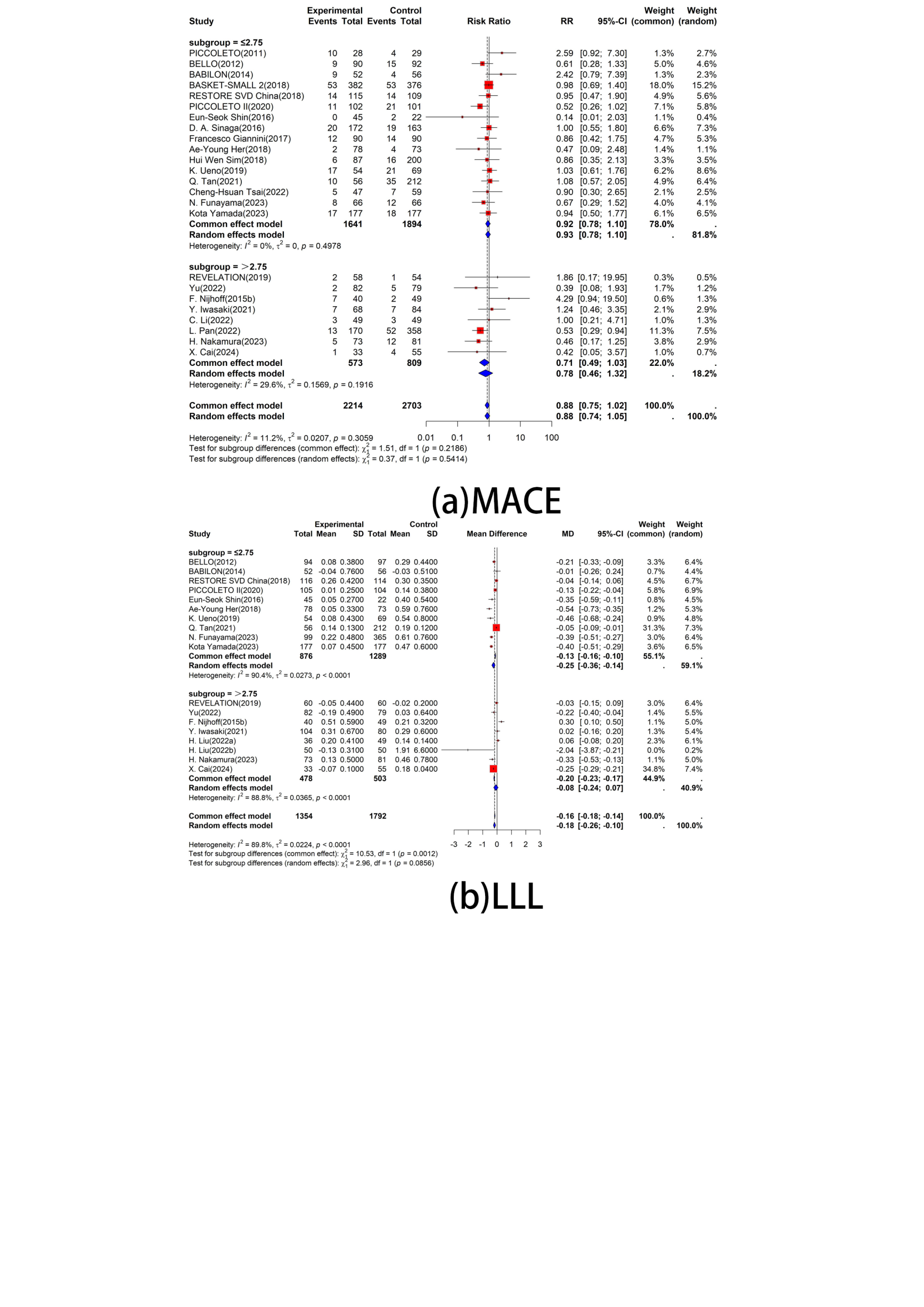


Abbreviations: MACE, major adverse cardiac event; LLL, late lumen loss; RR, risk ratio; MD, mean differences; CI, confidence interval

**Supplementary Figure 8.Forest plot of primary outcomes stratified by DAPT.**


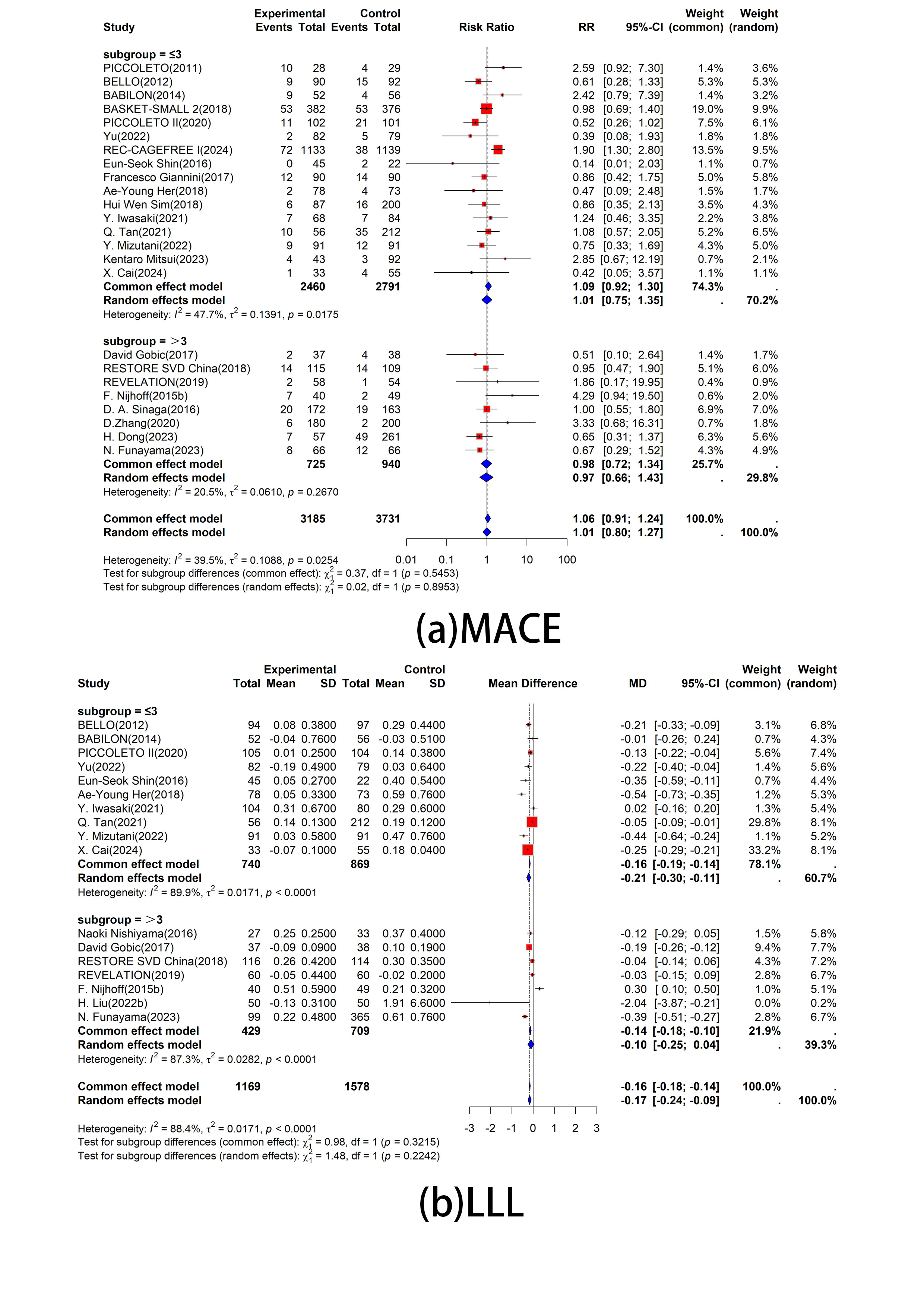


Abbreviations: MACE, major adverse cardiac event; LLL, late lumen loss; RR, risk ratio; MD, mean differences; CI, confidence interval;DAPT, dual antiplatelet therapy

**Supplementary Figure 9. Forest plot of primary outcomes according to the routine use of intravascular imaging techniques.**


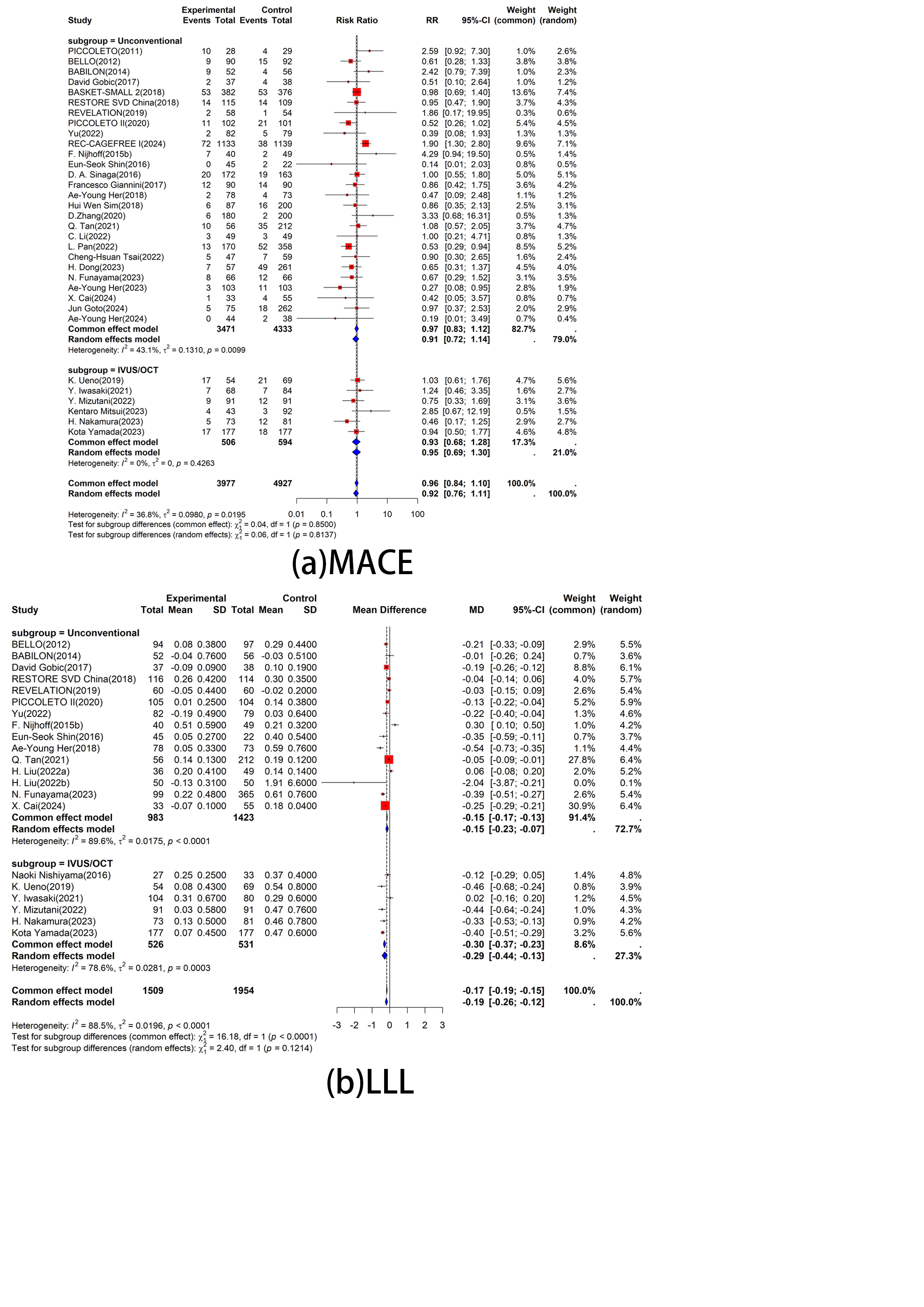


Abbreviations: MACE, major adverse cardiac event; LLL, late lumen loss; RR, risk ratio; MD, mean differences; CI, confidence interva

**Supplementary Figure 10. Forest plot of primary outcomes in patients with ACS.**

**
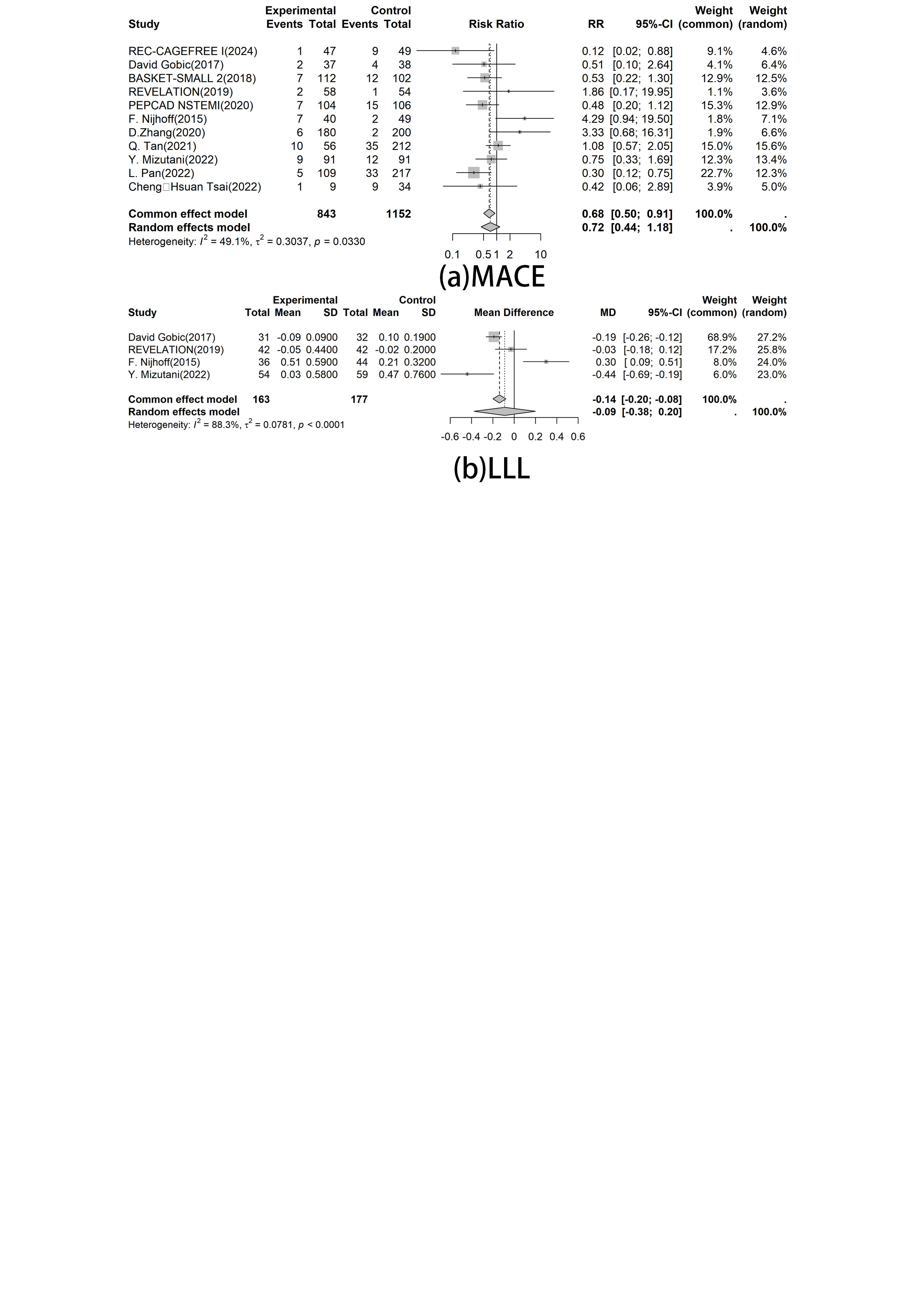
**

Abbreviations:ACS, acute coronary syndromes; RR, risk ratio; MD, mean differences; CI, confidence interval.

**Supplementary Figure 11. Forest plot of MACE in patients with diabetes mellitus.**

**
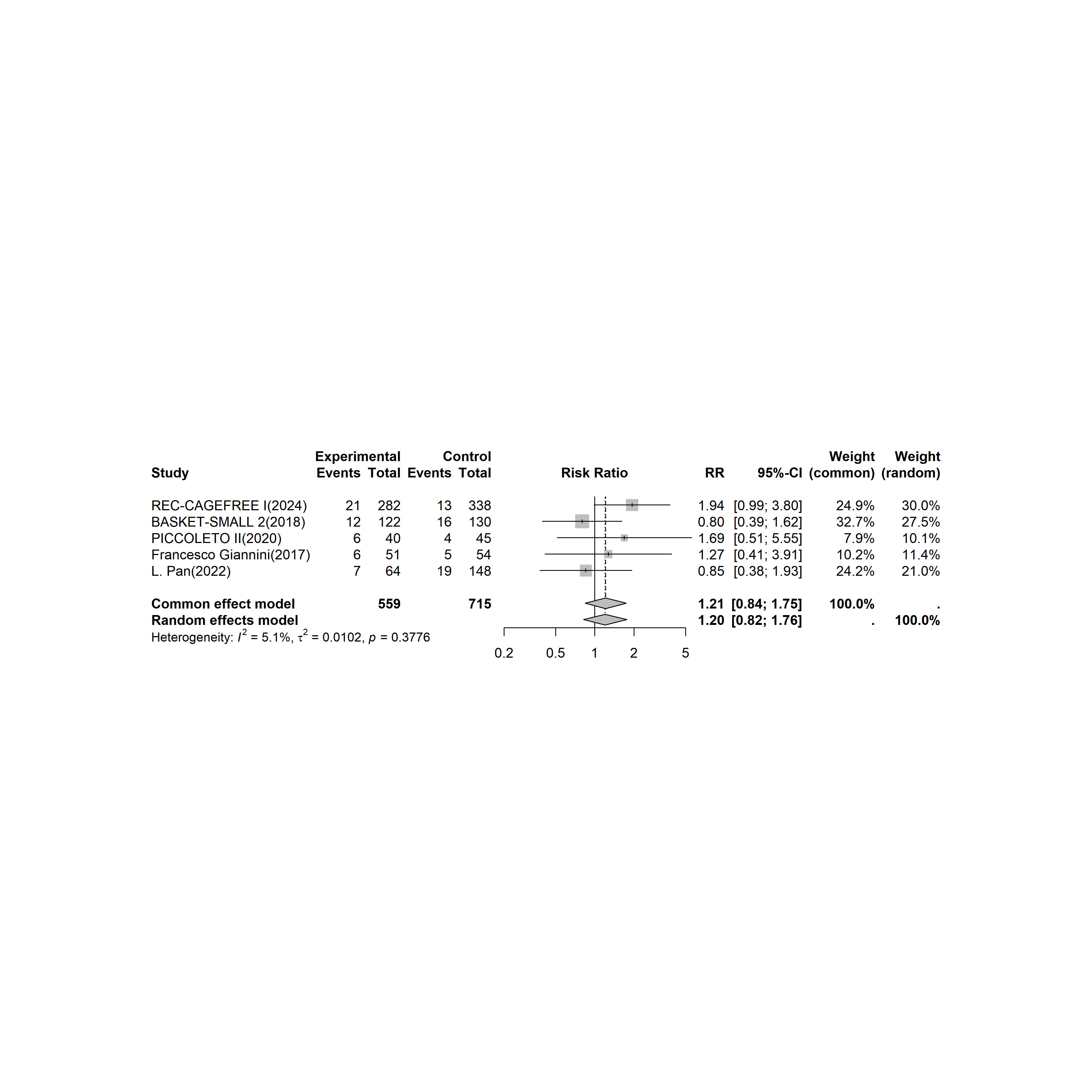
**

Abbreviations:MACE, major adverse cardiac events; RR, risk ratio; CI, confidence interval.

**Supplementary Figure 12. Forest plot of primary outcomes in bifurcation lesions.**

**
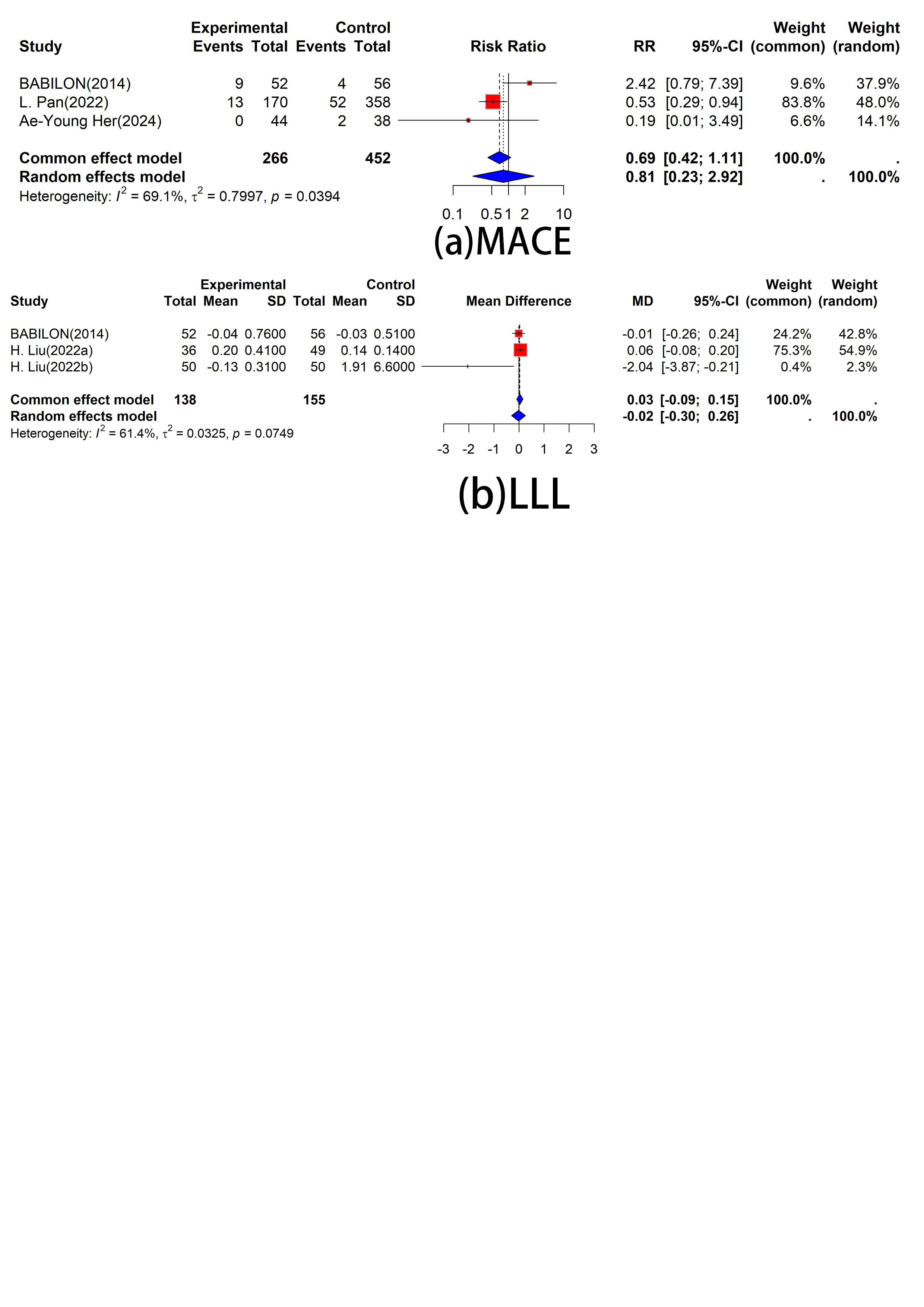
**

Abbreviations:MACE, major adverse cardiac events; RR, risk ratio; MD, mean differences; CI, confidence interval.

**Supplementary Figure 13. Funnel plot for publication bias of studies included in the meta-analysis**

**
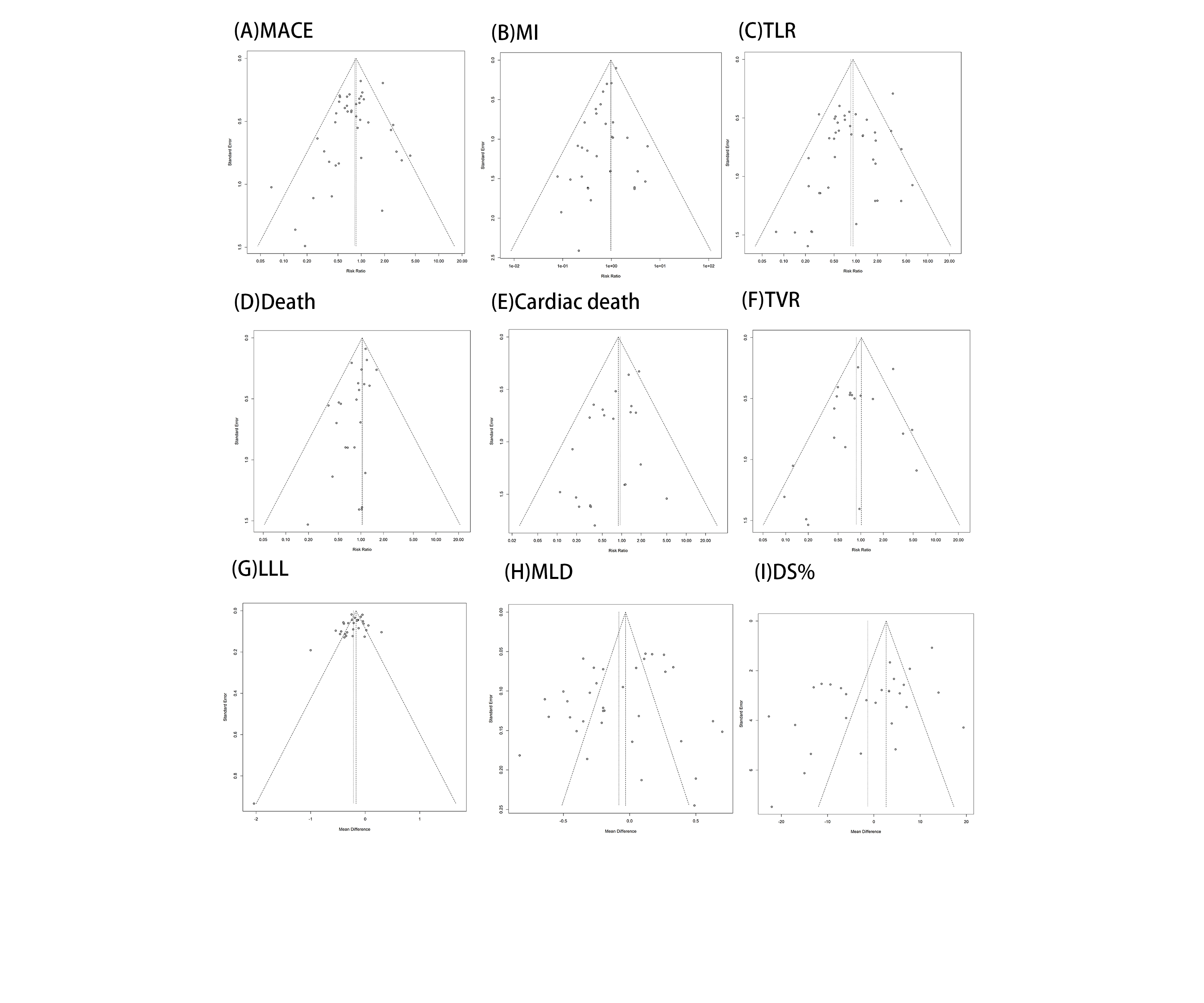
**

Abbreviations: MACE: major adverse cardiovascular events; MI: myocardial infarction; TLR: target lesion revascularization; TVR: target vessel revascularization,LLL: late lumen loss; MLD, minimum lumen diameter; DS%: percentage diameter stenosis.

**Supplementary Figure 14. Sensitivity analysis**

**
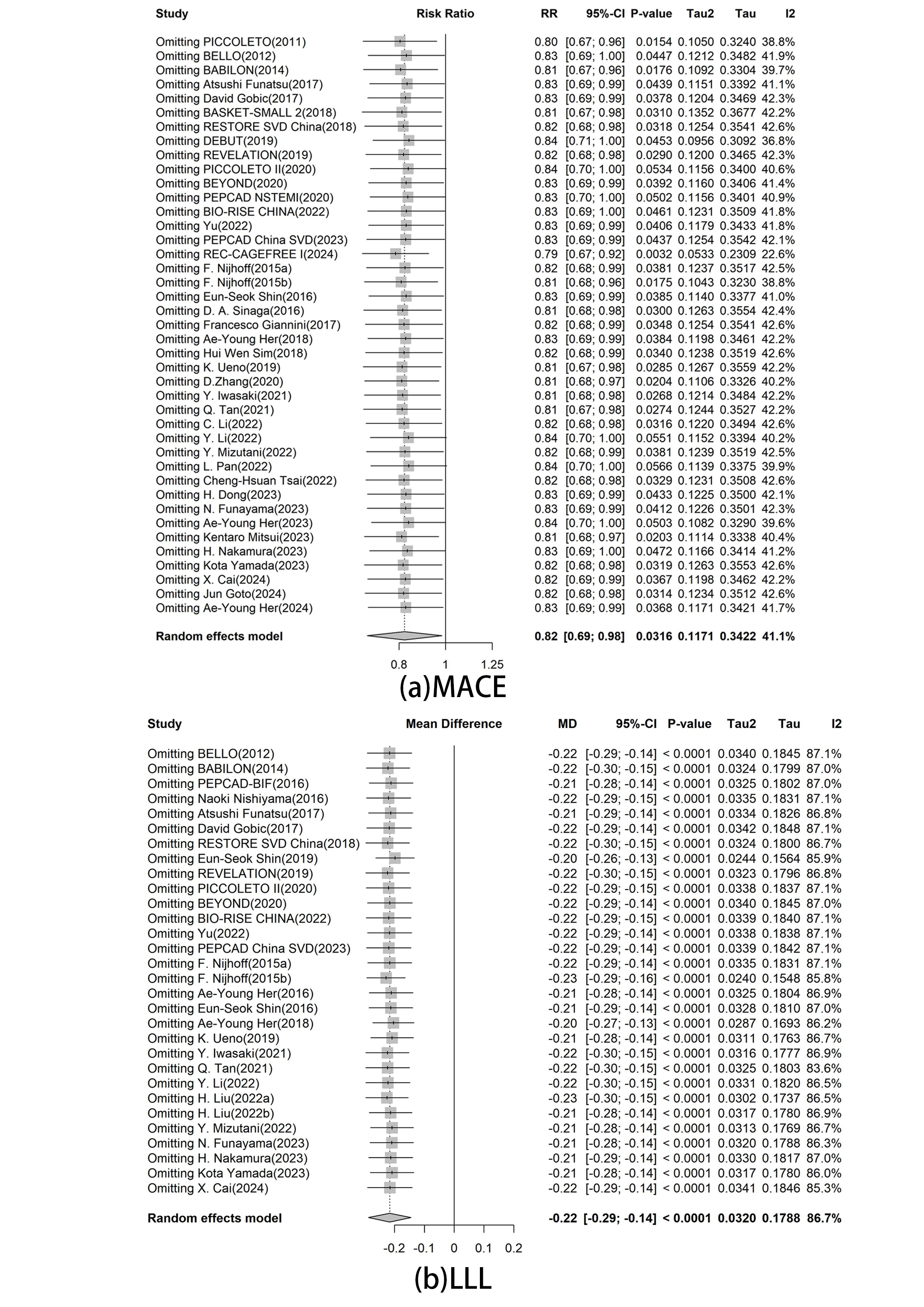
**

Abbreviations:LLL, Late Lumen Loss; MACE, Major Adverse Cardiovascular Event; CI, Confidence Interval.

**Supplementary Figure 15. Forest plot for the sensitivity analysis using standardized MACE stratified by comparator category (DCB vs uncoated device and DCB vs DES).**


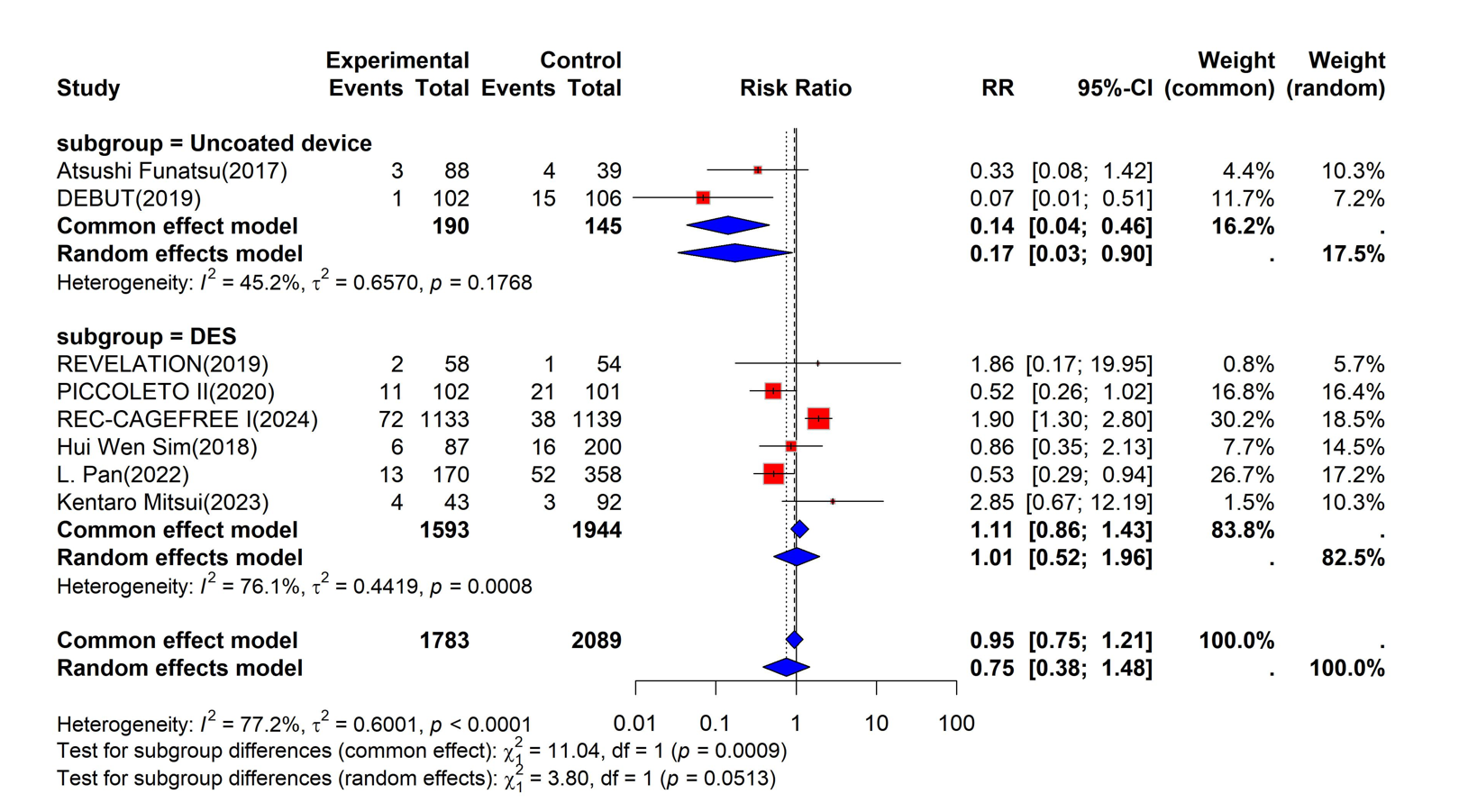


**Supplementary Figure 16. Forest plot for the sensitivity analysis using standardized MACE stratified by study design (RCT vs cohort).**


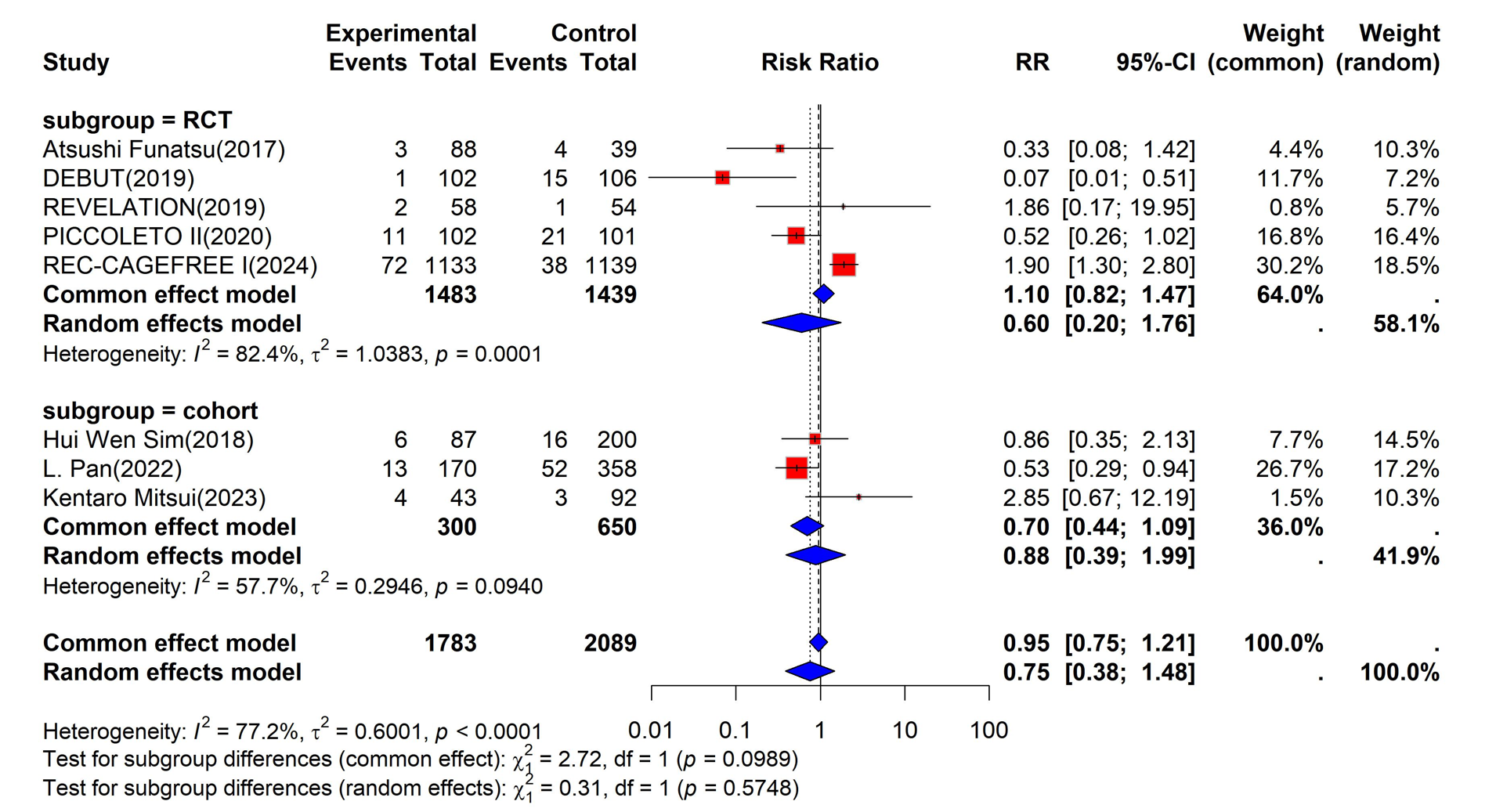


**Supplementary Figure 17.Forest plot for the sensitivity analysis using standardized MACE stratified by region (Asia vs Europe).**


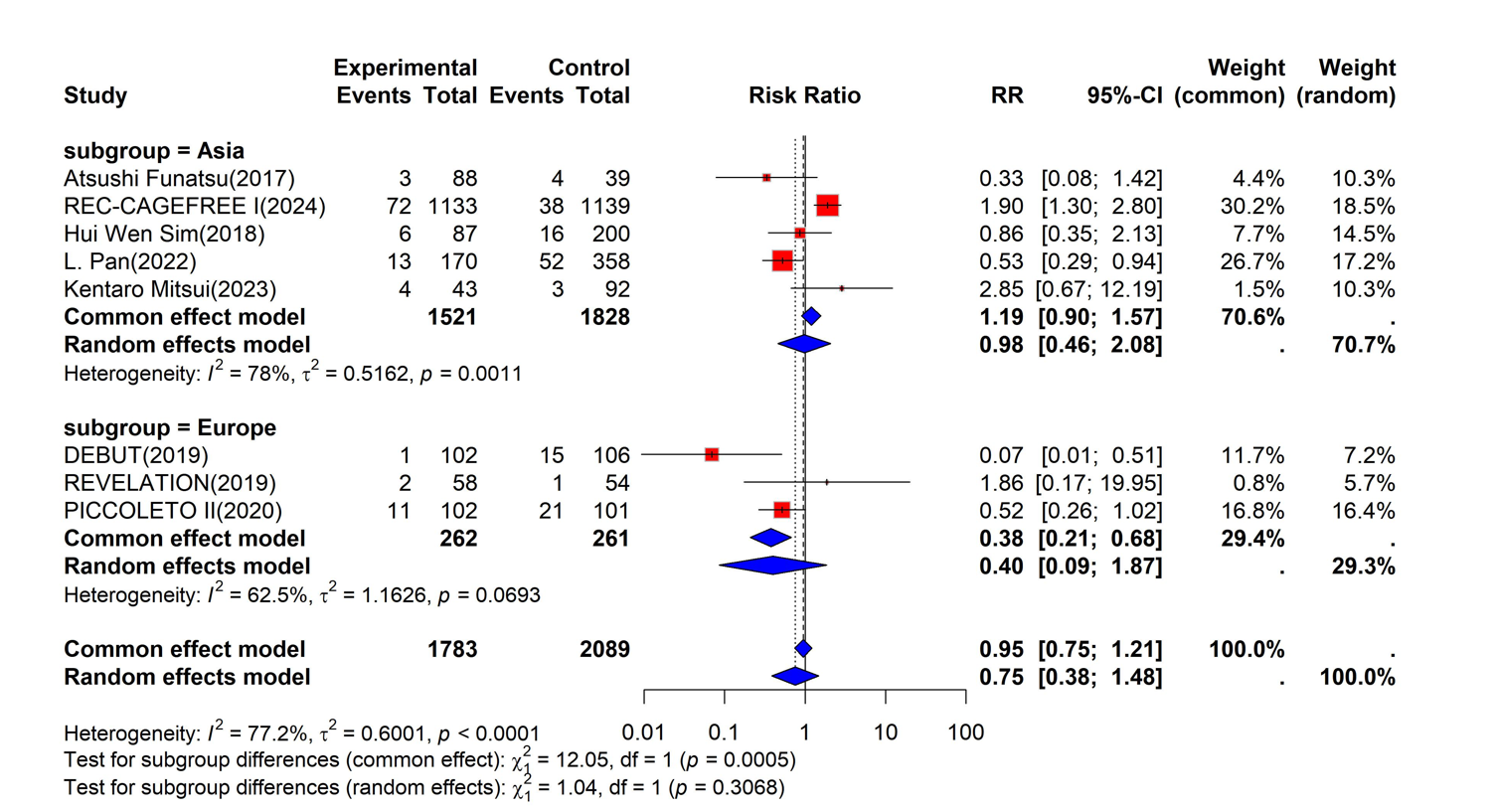

Supplement: Supplementary file 1 — Supporting Information Additional supporting information can be found online in the Supporting Information section. Table S1: Full search strategy and search terms. Table S2: Patient and disease characteristics in eligible studies. Table S3: Target lesion characteristics. Figure S1: Bias assessment of the included RCTs according to the Cochrane Collaboration. Figure S2: Forest plot of risk ratios for secondary clinical outcomes. Figure S3: Forest plot of mean differences (MDs) for secondary imaging outcomes. Figure S4: Forest plot of secondary clinical outcomes in RCTs and cohort studies. Figure S5: Forest plot of secondary clinical outcomes in RCTs and cohort studies. Figure S6: Forest plot of secondary imaging outcomes in RCTs and cohort studies. Figure S7: Forest plot of primary outcomes stratified by vessel diameter. Figure S8: Forest plot of primary outcomes stratified by DAPT. Figure S9: Forest plot of primary outcomes according to the routine use of intravascular imaging techniques. Figure S10: Forest plot of primary outcomes in patients with ACS. Figure S11: Forest plot of MACE in patients with diabetes mellitus. Figure S12: Forest plot of primary outcomes in bifurcation lesions. Figure S13: Funnel plot for publication bias of studies included in the meta‐analysis. Figure S14: Sensitivity analysis. Figure S15: Forest plot for the sensitivity analysis using standardized MACE stratified by comparator category (DCB vs. uncoated device and DCB vs. DES). Figure S16: Forest plot for the sensitivity analysis using standardized MACE stratified by study design (RCT vs. cohort). Figure S17: Forest plot for the sensitivity analysis using standardized MACE stratified by region (Asia vs. Europe). [file CDR-2026-5568664-s001.docx]
